# A quantitative approach to the intersectional study of mental health inequalities during the COVID-19 pandemic in UK young adults

**DOI:** 10.1007/s00127-023-02424-0

**Published:** 2023-01-24

**Authors:** Darío Moreno-Agostino, Charlotte Woodhead, George B. Ploubidis, Jayati Das-Munshi

**Affiliations:** 1 Centre for Longitudinal Studies, UCL Social Research Institute, University College London, 55-59 Gordon Square, London, WC1H 0NU UK; 2 ESRC Centre for Society and Mental Health, King’s College London, Melbourne House, 44-46 Aldwych, London, WC2B 4LL UK; 3 Department of Psychological Medicine, King’s College London, Institute of Psychiatry, Psychology & Neuroscience, 16 De Crespigny Park, London, SE5 8AF UK; 4 South London and Maudsley NHS Trust, London, UK

**Keywords:** Multilevel modelling, Intersectionality, MAIHDA, Multilevel analysis of individual heterogeneity and discriminatory accuracy, Intercategorical complexity

## Abstract

**Purpose:**

Mental health inequalities across social identities/positions during the COVID-19 pandemic have been mostly reported independently from each other or in a limited way (e.g., at the intersection between age and sex or gender). We aim to provide an inclusive socio-demographic mapping of different mental health measures in the population using quantitative methods that are consistent with an intersectional perspective.

**Methods:**

Data included 8,588 participants from two British cohorts (born in 1990 and 2000–2002, respectively), collected in February/March 2021 (during the third UK nationwide lockdown). Measures of anxiety and depressive symptomatology, loneliness, and life satisfaction were analysed using Multilevel Analysis of Individual Heterogeneity and Discriminatory Accuracy (MAIHDA) models.

**Results:**

We found evidence of large mental health inequalities across intersectional strata. Large proportions of those inequalities were accounted for by the additive effects of the variables used to define the intersections, with some of the largest gaps associated with sexual orientation (with sexual minority groups showing substantially worse outcomes). Additional inequalities were found by cohort/generation, birth sex, racial/ethnic groups, and socioeconomic position. Intersectional effects were observed mostly in intersections defined by combinations of privileged and marginalised social identities/positions (e.g., lower-than-expected life satisfaction in South Asian men in their thirties from a sexual minority and a disadvantaged childhood social class).

**Conclusion:**

We found substantial inequalities largely cutting across intersectional strata defined by multiple co-constituting social identities/positions. The large gaps found by sexual orientation extend the existing evidence that sexual minority groups were disproportionately affected by the pandemic. Study implications and limitations are discussed.

**Supplementary Information:**

The online version contains supplementary material available at 10.1007/s00127-023-02424-0.

## Introduction

The quantitative study of health inequalities has often been inadequately underpinned by social theory [1]. Quantitative studies have frequently focused on examining inequalities in relation to broad social categories such as gender, race/ethnicity, and socioeconomic position (SEP), with the social forces driving these inequalities [2–5] often being under-acknowledged. This can contribute to the perpetuation of deficit-based or damage-centred perspectives which locate the “problem” of inequality within the group(s) being examined rather than the underlying structures and processes [6, 7], which serve as the up-stream, fundamental causes of such inequalities [5]. Similarly, the complexity of personal experience, in that people occupy more than one social identity/position which can include a mix of advantaged and disadvantaged identities/positions that are dynamic and context-dependent [8–10], gets frequently under-recognised.

Intersectionality theory [11] supports a move away from some of these issues by highlighting that social identities and positions are “interdependent and mutually constitutive rather than independent and uni-dimensional” [12]. It acknowledges that, due to interlocking systems of oppression, the experiences of a person living at a particular intersection (e.g., Black woman) cannot be understood by independently looking at the experiences associated to each of the identities and positions that define it (in the same example, the experiences associated with being Black and a woman).

Although intersectional research poses challenges for both qualitative and quantitative methodological approaches [12], it has relied mostly on qualitative methods. Quantitative approaches to intersectionality have been criticised for their potential to unintentionally reinforce the idea that the observed inequalities may be natural or intractable [13, 14] and “blunt [the] critical edge and transformative aims” of intersectionality [3] by simply describing those inequalities. Intercategorical approaches to intersectional complexity [15], where analytical categories (e.g., based on gender) are used to explore inequalities, and the focus on identifying significant differences across such categories (a focus that has been named “intersectionality as a testable hypothesis”) [11], have also been criticised. By focusing on the differences *between* groups, these approaches may dismiss the differences *within* those groups, unintentionally reinforcing the idea that they are homogeneous [11]. Furthermore, the use of the most privileged categories (e.g., White, male) as the reference can implicitly maintain the idea of dominant groups being the standard to which the rest of categories should be compared [16]. This can also result in a lack of evidence on intersections defined by combinations of privileged and marginalised identities and positions, which is essential to understand and address health inequalities [17].

Nonetheless, quantitative approaches provide unique opportunities to accurately document population health inequalities [14]. First, many of the above-mentioned critiques are not inherent to quantitative methods [12, 18]. Categories can be provisionally adopted to explore inequalities across intersections [15] and acknowledged as proxies for the interlocking systems of oppression [14, 17]. Furthermore, aspects such as SEP reflect material conditions rather than social constructions. In addition, novel quantitative approaches [18–24] can help overcome some of the critiques. Multilevel Analysis of Individual Heterogeneity and Discriminatory Accuracy (MAIHDA) models [17, 23] constitute a paradigmatic example. Unlike more traditional quantitative intercategorical approaches (e.g., fixed-effects regression models with interaction terms), MAIHDA models open the way to provide evidence at intersections that would otherwise be overlooked [18, 21]. Moreover, they provide estimates of the variability/heterogeneity within those intersections and the proportion of variability that is attributable to differences between them [known as Variance Partition Coefficient (VPC) or Intra-Class Correlation (ICC)]. Such estimates can be interpreted as a measure of the “discriminatory accuracy” of the categories provisionally adopted to define the intersections, and can be relevant to inform public policy, because targeting interventions at specific intersections when very little of the variability is attributable to differences between intersections (i.e., when discriminatory accuracy is low) may lead to ineffective interventions [23].

MAIHDA models focus on the difference between the *expected* levels at particular intersections, operationalised as the sum of the effects of each of the categories that define them (i.e., the “sum of the parts” or the additive effects), and the *observed* levels at those intersections. Such “excess” or residual effect represents what is above and beyond the “sum of the parts”, what is unique to that particular intersection: the “intersectional effect”. Intersectional effects represent the impact of experiences of marginalisation and/or privilege due to interlocking systems of oppression in the outcomes under study [25]. The distinction between intersectional “experiences” and “effects” is crucial: failure to find significant intersectional effects does not preclude the existence of different experiences lived by different intersections [21, 25]. Hence, MAIHDA models provide the opportunity to study intersectional complexity from one angle, which can then be complemented by qualitative, experiential, and other quantitative approaches for a more complete understanding [18, 26]. This angle is descriptive in the sense that it does not engage in the statistical analysis of causal processes driving the inequalities described [13]. However, by explicitly engaging with social theory, they can situate those inequalities in the context of the underpinning social processes causing them, thus “maintain(ing) the critical and transformative edge of intersectionality” [1].

### An applied example: mental health inequalities during the COVID-19 pandemic in the UK

The onset of the COVID-19 pandemic has had unequal implications for different groups within the population [27, 28]. Evidence suggests disproportional mental health effects among disadvantaged population groups including adolescents and young adults, women, racialised and ethnically minoritised groups, sexual and gender minority groups, and those in more disadvantaged SEP [29]. UK-based research replicates these findings in outcomes such as anxiety and depressive symptomatology, psychological distress, loneliness, and life satisfaction [30–43]. In most cases, however, mental health inequalities by different social identities and positions have been reported independently from each other. Hence, the mutual co-constitution of those broader social categories has been left unacknowledged (or has been acknowledged in a very limited way, such as at the intersection between age and sex or gender) [12, 18].

Using MAIHDA models, this study aims to provide evidence within the UK on mental health across multiple intersectional positions defined by categories closely tied to social power such as age, sex, race/ethnicity, sexual orientation, and SEP. This will first provide a “socio-demographic mapping” of the levels of different mental health measures within the population [14], which in turn will support the development of hypotheses for further research and suggest avenues for public health resource allocation.

## Methods

### Sample

This study focused on the most recent assessment of two British cohorts: Next Steps (NS) [44] and Millennium Cohort Study (MCS) [45], with participants born in 1990 and 2000–2002, respectively. This assessment took place in February/March 2021, during the third nationwide lockdown [46], as part of the third wave of the ‘COVID-19 Survey’ [47]. Both cohorts implemented oversampling methods to ensure representation from marginalised populations [44, 45]. We focused on participants who were alive and still residing in the UK during the third wave of the COVID-19 Survey (February/March 2021). Due to the use of web and telephone interviews, the largest response rates within the target population were achieved in this wave of the COVID-19 Survey: 26.4% (NS) and 23.0% (MCS). Overall, 8588 participants (4167 from NS, 4421 from MCS) were included. All participants provided informed consent. Further details on the sample and procedure are available elsewhere [47].

### Measures

#### Outcomes

Measures of anxiety symptomatology, depressive symptomatology, loneliness, and life satisfaction were collected using the same assessment tools across the two cohorts. Anxiety and depressive symptomatology were measured using the 2-item versions of the Generalised Anxiety Disorder (GAD-2) [48] and Patient Health Questionnaire (PHQ-2) [49]. These questionnaires enquire about how frequently the respondent has been bothered by core experiences of anxiety or depression, respectively, with scores ranging from 0 (lowest anxiety/depression) to 6 (highest anxiety/depression). Loneliness was measured with the University of California Los Angeles 3-item loneliness scale (UCLA-3) [50], which enquires about the extent to which the respondent has felt lack of companionship, left out, or isolated from others, and with scores ranging from 3 (lowest loneliness) to 9 (highest loneliness). Life satisfaction was measured with the Office for National Statistics (ONS) single question [51], with scores ranging from 0 (lowest life satisfaction) to 10 (highest life satisfaction).

#### Indicators/proxies for social identities/positions

Cohort/generation was assigned from the cohort of provenance. NS participants were in their early 30s at the time of the interview, whereas MCS participants were in their late teens/early 20s.

Information on birth sex as a binary variable (female or male) was obtained from the parents in the earlier waves.

Information on race/ethnicity corresponded to the most recent self-designated racial/ethnic group, complemented by the parents’ report wherever the former was not available. Responses were obtained following the ONS criteria [52] and, due to the small number of participants in some of the individual groups, grouped into White (including all White groups), Mixed (including all Mixed groups), South Asian (including Indian, Pakistani, and Bangladeshi groups), Black (including Black African, Black Caribbean, and Black British groups), and Other (including all ethnicities not included in the previous groups).

Self-reported information on sexual orientation was obtained from participants. Due to the small number of cases in some of the minority categories, we grouped participants into heterosexual versus sexual minority (including bisexual, gay/lesbian, and other) for analyses.

The residential Index of Multiple Deprivation (IMD) was used as an indicator of the current household SEP. A binary variable indicating whether the person lived in an area above (less deprived) or below (more deprived) the within-country median IMD rank was derived (the methodology used to generate IMDs varies across UK countries [53]). Self-reported information on housing tenure, collected during the COVID-19 Survey and grouped into Owners (including part owners) and Not owners, was used as an alternative indicator of the current household SEP. Finally, harmonised data on parental social class at age 11/14 years were used as an indicator of the household SEP during childhood [54], grouped into Non-manual/advantaged (including Professional, Managerial and Technical, and Skilled non-manual groups) and Manual/disadvantaged (including Skilled manual, Partly skilled, and Unskilled). Residential IMD was prioritised as SEP indicator due to the smaller number of missing data.

Intersectional strata were first generated not including socioeconomic indicators, resulting in 2 (cohorts/generations) * 2 (birth sex) * 5 (ethnicity groups) * 2 (sexual orientation) = 40 intersectional strata (stratification 40). Strata including indicators of SEP were then generated using either residential IMD rank (stratification 80a), current housing tenure (stratification 80b), or harmonised childhood social class (stratification 80c), resulting in up to 80 strata reflecting the intersection with different aspects of the SEP.

### Statistical analysis

We used MAIHDA models [17, 23] to obtain estimates of the residual/intersectional effects (i.e., what is beyond what would be *expected* based on the fixed/main/additive effects, conceptually similar to interaction effects) and predicted effects (including both the *expected* and residual/intersectional effects) at the different intersectional strata in each outcome. We first estimated intercepts-only (or “null” [17, 25]) models with no predictors to obtain estimates of the degree of clustering or correlation within the strata (or, analogously, the proportion of the variance explained by differences across strata) (*VPC*_intercepts-only_). Then, main models were estimated including the variables adopted to define the intersectional strata as predictors. The fixed effects of each of those predictors (cohort/generation, birth sex, racial/ethnic group, sexual orientation, and the appropriate SEP indicator depending on the stratification used) represent the main/additive effects of the specific category across all intersections (non-intersectional effects). The VPC from the main models (VPC_main_) returns information on the degree of clustering or correlation *within* intersectional strata (or, analogously, the proportion of the variance explained by differences across strata) after accounting for the fixed (or main, or additive) effects of each of the variables used to define these (the “sum of the parts”) [17]. The percentage of between-strata variance accounted for by the inclusion of those main/additive effects, or Proportional Change in Variance (PCV), was obtained as

$$PCV=\left(1- \frac{{VPC}_{main}}{{VPC}_{intercepts-only}}\right) \times 100.$$

Models were estimated using the four above-mentioned stratifications (40, 80a, 80b, and 80c). Following the procedure and code laid out by Dr Claire Evans [21], models were first estimated using Bayesian Markov Chain Monte Carlo (MCMC) procedures [55] with diffuse priors, initialisation values obtained from analogous models estimated with quasi-likelihood methods, and 50,000 iterations with a burn-in period of 5000 iterations and thinning every 50 iterations. Stratum-specific residual values (the intersectional effects [25]) and predicted values (including both the stratum-specific residuals and the fixed effects of each of the social identities/positions defining the stratum) were obtained from the main models, and 95% credible intervals (CI) were constructed using the 2.5 and 97.5 percentiles of those values across the MCMC iterations.

Initial checks (Supplementary Appendix S1) suggested that survey non-response was introducing bias. Based on these results, Bayesian MCMC MAIHDA models may be adequate to provide a socio-demographic mapping of the mental health levels among the survey respondents. Weighted analyses to account for the survey design and non-response are not yet implemented in Bayesian MCMC MAIHDA models. We estimated an identical set of models with maximum-likelihood (ML) estimation using weights to account for survey design and non-response, thus increasing the generalisability of the results beyond the survey respondents to each survey’s target population. One caveat is that ML estimation does not provide confidence intervals for the stratum-specific residuals (the intersectional effects).

Fixed-effects multiple regression models including the interaction across all the variables adopted to define the intersections were estimated for comparison purposes. Details on the rationale for these additional analyses are available in Supplementary Appendix S2.

MCMC MAIHDA models were estimated in MLwiN version 3.01 [56], using the *runmlwin* function [57] in Stata/MP 17.0 [58]. ML MAIHDA models and multivariable regression models were estimated in Stata/MP 17.0.

## Results

Most participants across both cohorts were female, White, and heterosexual (Supplementary Table S1). Sample sizes varied across models due to different missingness in the outcomes and SEP indicators. When accounting for the SEP indicators, some strata corresponding to intersections with racial/ethnic and sexual minority groups had no observations (Supplementary Table S2). There was a large variability in the number of observations by stratum, ranging from 1 to 1669, and the percentage of strata with 20 or more observations ranged from 45.0% to 62.5% (Supplementary Table S3).

As shown in Table 1, the degree of clustering into the intersectional strata (or, analogously, the proportion of variance explained by differences across strata) before including the fixed effects of the variables used to define them (the VPC_intercepts-only_) was generally larger for anxiety and depressive symptomatology than for loneliness and life satisfaction. This suggests that the discriminatory accuracy of the variables defining the intersections was generally larger for anxiety and depressive symptomatology. The discriminatory accuracy varied across outcomes when using different SEP indicators, being largest for anxiety and depressive symptomatology when using IMD rank, housing tenure for loneliness, and childhood social class for life satisfaction. PCVs under MCMC (unweighted) were large in all cases (> 90.0%), indicating that the main/additive effects accounted for most of the variability *between* clusters. PCVs were generally smaller under ML (weighted) due to larger proportions of residual variance between strata (VPC_main_), suggesting larger intersectional effects.

**Table 1:** Number of cases and measures of clustering within strata and percentage of between-strata variance accounted for by the main effects of the variables defining the strata of each outcome * intersectional strata combination

			Markov Chain Monte Carlo estimation, unweighted			Maximum-likelihood estimation, weighted		
Outcome	Stratification	*N* obs	VPC_intercepts-only_	VPC_main_	PCV	VPC_intercepts-only_	VPC_main_	PCV
GAD-2 (anxiety symptomatology)	40	7963	0.114 (0.067, 0.187)	0.003 (< 0.001, 0.011)	97.2%	0.155 (0.084, 0.268)	0.007 (0.001, 0.056)	95.4%
	80a	7896	0.108 (0.071, 0.163)	0.003 (< 0.001, 0.011)	97.0%	0.155 (0.101, 0.229)	0.031 (0.010, 0.092)	80.2%
	80b	7325	0.103 (0.066, 0.151)	0.003 (0.001, 0.010)	96.7%	0.146 (0.091, 0.227)	0.016 (0.003, 0.072)	89.3%
	80c	7065	0.104 (0.065, 0.153)	0.004 (< 0.001, 0.013)	96.5%	0.116 (0.075, 0.175)	0.004 (< 0.001, 0.033)	96.5%
PHQ-2 (depressive symptomatology)	40	7962	0.092 (0.052, 0.154)	0.004 (< 0.001, 0.014)	95.5%	0.128 (0.065, 0.235)	0.018 (0.003, 0.104)	86.0%
	80a	7895	0.098 (0.062, 0.147)	0.004 (< 0.001, 0.015)	95.5%	0.151 (0.093, 0.235)	0.060 (0.021, 0.157)	60.5%
	80b	7322	0.093 (0.056, 0.141)	0.005 (0.001, 0.013)	94.5%	0.138 (0.085, 0.215)	0.049 (0.021, 0.110)	64.7%
	80c	7061	0.090 (0.054, 0.137)	0.006 (0.001, 0.018)	93.7%	0.078 (0.048, 0.124)	0.007 (0.001, 0.043)	91.4%
UCLA-3 (feelings of loneliness)	40	7949	0.056 (0.031, 0.099)	0.002 (< 0.001, 0.009)	95.9%	0.064 (0.033, 0.119)	0.004 (< 0.001, 0.724)	93.9%
	80a	7882	0.060 (0.036, 0.095)	0.004 (< 0.001, 0.011)	94.2%	0.073 (0.047, 0.113)	0.016 (0.004, 0.066)	78.0%
	80b	7312	0.062 (0.039, 0.096)	0.006 (0.002, 0.014)	90.3%	0.108 (0.069, 0.165)	0.054 (0.025, 0.113)	50.4%
	80c	7053	0.060 (0.034, 0.099)	0.003 (< 0.001, 0.011)	94.2%	0.052 (0.029, 0.093)	0.003 (< 0.001, 0.060)	94.1%
ONS life satisfaction question	40	8005	0.049 (0.024, 0.085)	0.001 (< 0.001, 0.005)	97.7%	0.075 (0.041, 0.133)	< 0.001 (< 0.001, < 0.001)	> 99.9%
	80a	7938	0.053 (0.030, 0.087)	0.002 (< 0.001, 0.007)	96.8%	0.093 (0.052, 0.162)	0.024 (0.005, 0.113)	74.1%
	80b	7359	0.053 (0.029, 0.084)	0.004 (0.001, 0.011)	93.0%	0.119 (0.080, 0.172)	0.048 (0.023, 0.099)	59.5%
	80c	7097	0.062 (0.035, 0.103)	0.001 (< 0.001, 0.006)	98.1%	0.122 (0.064, 0.219)	0.070 (0.022, 0.200)	42.9%

*GAD-2* 2-item Generalised Anxiety Disorder questionnaire, *N obs* number of observations, *ONS* Office for National Statistics, *PCV* proportional change in variance (expressed as a percentage), *PHQ-2* 2-item depression Patient Health Questionnaire, *UCLA-3* 3-item University of California Los Angeles loneliness scale, *VPC* variance partition coefficient. Stratification 40 are defined by cohort * birth sex * racial/ethnic group * sexual orientation; stratification 80a include stratification 40 * within-country index of multiple deprivation rank of the residential area; stratification 80b include stratification 40 * housing tenure; stratification 80c include stratification 40 * parental social class during childhood (age 11/14)

Results from the MCMC (unweighted) models using 40 intersectional strata evidenced large inequalities across strata in the predicted values of all outcomes (Supplementary Figure S1). Although most differences were accounted for by the main/additive effects of the variables defining the strata, and all intersectional effects overlapped with zero (no effect), some strata had higher- or lower-than-expected levels (Supplementary Figure S2). Results from the ML (weighted) models (Supplementary Figures S3-S4) were similar, with most of the differences across strata being accounted for by the main effects as indicated by the high PCVs (Table 1).

**Fig. 1:**
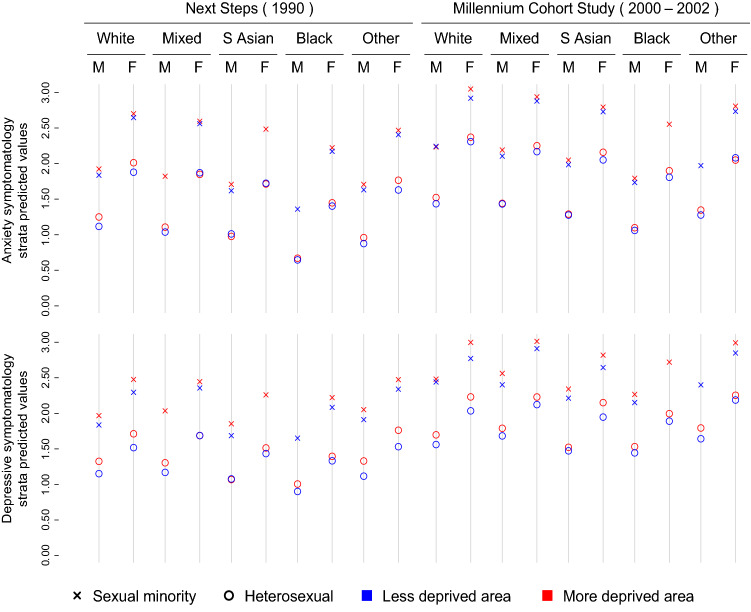
Anxiety and depressive symptomatology predicted values of each intersectional stratum using residential Index of Multiple Deprivation (IMD) rank as the indicator of socioeconomic position. Markov Chain Monte Carlo (MCMC) estimation, unweighted results. *M* male, *F* female. White includes all White groups; South Asian includes Bangladeshi, Indian, and Pakistani groups; Black includes Black African, Black Caribbean, and Black British groups; Other includes all other ethnic group not included in the other categories

**Fig. 2:**
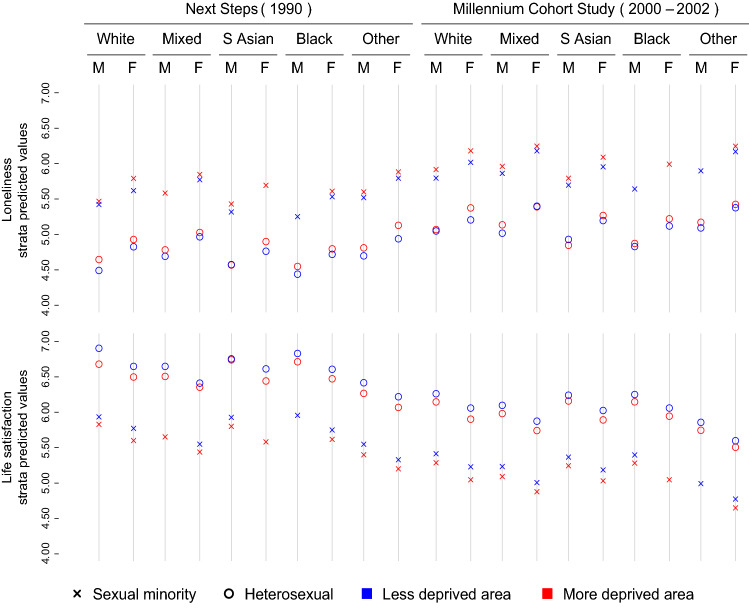
Loneliness and life satisfaction predicted values of each intersectional stratum using residential Index of Multiple Deprivation (IMD) rank as the indicator of socioeconomic position. Markov Chain Monte Carlo (MCMC) estimation, unweighted results. *M* male, *F* female. White includes all White groups; South Asian includes Bangladeshi, Indian, and Pakistani groups; Black includes Black African, Black Caribbean, and Black British groups; Other includes all other ethnic group not included in the other categories

Figure 1 and Fig. 2 provide a ‘socio-demographic mapping’ of the predicted levels in the different mental health outcomes using residential IMD rank as SEP indicator according to the MCMC (unweighted) estimation, evidencing large inequalities across intersectional strata. Fixed (main/additive) and random effects from these MCMC models are included in Supplementary Table S4. Most of the privileged categories (male, heterosexual, socioeconomically advantaged) showed better outcome levels, with large and consistent gaps across sexes, sexual orientations, and cohorts/generations (participants in their 30s showed better results than those a decade younger across all outcomes). Inequalities by IMD rank were comparatively smaller. Black participants generally showed the lowest levels of anxiety and depressive symptomatology and loneliness. This was not the case for life satisfaction, where Black and White participants showed fairly similar results across intersections with other variables, and the lowest levels were observed among those in the “Other” ethnicity group, which were also among the intersections showing the worst mental health outcomes. Using different SEP indicators (Supplementary Figures S5–S6) led to very similar results, although gaps by SEP were typically larger when using housing tenure as indicator. The divide by sexual orientation was consistent across all outcomes, accounting for some of the largest gaps in all outcomes.

The ‘socio-demographic mapping’ of the predicted values at each intersection was more heterogeneous when accounting for survey design and non-response (Supplementary Figures S7-S8). The fixed/main/additive effects from these models (Supplementary Table S5) were, however, largely similar to those from the unweighted models, and sexual orientation was again associated with most of the largest gaps across all stratifications and outcomes. Most differences in fixed effects between weighted and unweighted approaches were found by racial/ethnic group. Being in the “Other” ethnicity group was associated with worse levels in anxiety, whereas those in the Mixed ethnicity group showed worse loneliness and life satisfaction outcomes.

**Fig. 3:**
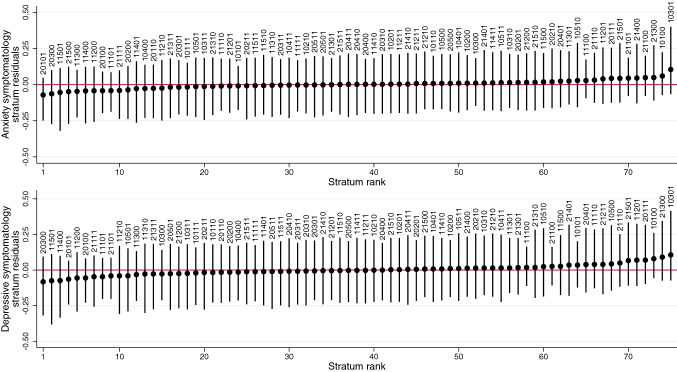
Anxiety and depressive symptomatology residual values (intersectional effects) and 95% credible intervals of each intersectional stratum using residential Index of Multiple Deprivation (IMD) rank as the indicator of socioeconomic position. Markov Chain Monte Carlo (MCMC) estimation, unweighted results. Strata defined by generation/cohort (first digit: 1 Next Steps/1990, 2 Millennium Cohort Study/2000–2002), birth sex (second digit: 0 Male, 1 Female), ethnicity (third digit: 1 White, 2 Mixed, 3 South Asian, 4 Black, 5 Other), sexual orientation (fourth digit: 0 Heterosexual, 1 Sexual minority), residential IMD rank (fifth digit: 0 More deprived, 1 Less deprived)

**Fig. 4:**
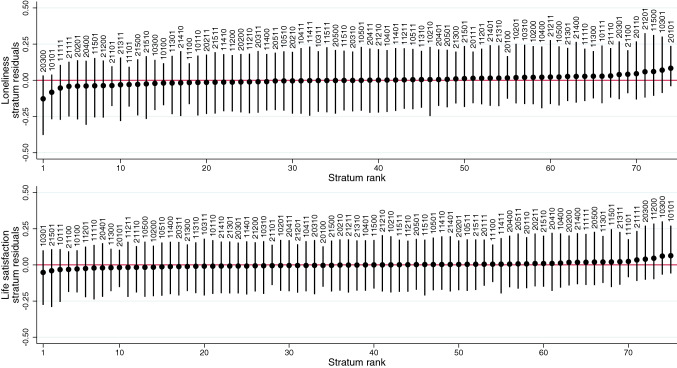
Loneliness and life satisfaction residual values (intersectional effects) and 95% credible intervals of each intersectional stratum using residential Index of Multiple Deprivation (IMD) rank as the indicator of socioeconomic position. Markov Chain Monte Carlo (MCMC) estimation, unweighted results. Strata defined by generation/cohort (first digit: 1 Next Steps/1990, 2 Millennium Cohort Study/2000–2002), birth sex (second digit: 0 Male, 1 Female), ethnicity (third digit: 1 White, 2 Mixed, 3 South Asian, 4 Black, 5 Other), sexual orientation (fourth digit: 0 Heterosexual, 1 Sexual minority), residential IMD rank (fifth digit: 0 More deprived, 1 Less deprived)

Figure 3 and Fig. 4 show the residual values (intersectional effects) of each intersectional stratum using residential IMD as SEP indicator according to the MCMC (unweighted) estimation (similar plots using the alternative SEP indicators are available in Supplementary Figures S9–S12). All intersectional effects’ CIs overlapped with or were very close to zero (no effect). The only significant intersectional effect corresponded to the loneliness levels of the stratum including White heterosexual males in their 30s owning/part owning a house, which were *M*_*residual*_ = − 0.19 (95% CI − 0.39, − 0.005) lower-than-expected. Some strata at the intersection between privileged and marginalised social identities/positions tended to have worse-than-expected (e.g., South Asian heterosexual males in their 30s living in less deprived areas) or better-than-expected (e.g., South Asian heterosexual males in their teens/20s living in more deprived areas) outcomes.

Larger residual values (intersectional effects) at some intersections were found in the weighted analyses (ML estimation, Supplementary Figures S13–18). Most of the largest intersectional effects corresponded to strata at the intersection of privileged and marginalised social identities/positions. For instance, the largest worse-than-expected levels were found for anxiety among South Asian heterosexual men in their thirties living in less deprived areas (*M*_*residual*_ = 0.52); for depression among heterosexual men in their 30s from the “Other” ethnicity group living in more deprived areas (*M*_*residual*_ = 1.10) (Supplementary Figure S13); for loneliness among South Asian heterosexual women in their teens/20s owning a house (*M*_*residual*_ = 1.05) (Supplementary Figure S16); and for life satisfaction among South Asian men in their thirties from a sexual minority and a disadvantaged social class at childhood (*M*_*residual*_ = − 1.52) (Supplementary Figure S18).

### Comparison with fixed-effects multiple regression approach

Results from the fixed-effects multiple regression approach are included in Supplementary Tables S6-S9. Several interaction terms were statistically significant. In line with the differences across the MCMC unweighted and ML weighted MAIHDA models, the unweighted and weighted regression models’ results varied in some cases, with some interaction terms becoming statistically significant after accounting for the survey and non-response weights, often involving sexual and ethnic minorities. Many of the significant interaction terms found under both approaches were based on very few (down to two) observations and using a specific intersection (White heterosexual males in their 30s in a disadvantaged SEP) as reference.

## Discussion

We aimed to provide a “socio-demographic mapping” [14] of the mental health inequalities within the UK population during the COVID-19 pandemic from an intersectional perspective, using MAIHDA models. We documented levels of anxiety, depression, loneliness, and life satisfaction across multiple intersecting social identities/positions tied to social power and explored whether there were intersectional effects observable above and beyond the effects associated with any identity/position in isolation. In our first approach, similar to previous MAIHDA applications [21, 25, 59, 60], we found that, among the study participants, most of the differences across intersectional strata were accounted for by the additive effects of the social identities/positions used to define those intersections. Our second approach aimed to account for the biasing effect of differential non-response to make the results generalisable beyond the study participants. Using this approach, we found even larger inequalities across strata and different-than-expected outcome levels in some intersectional strata, defined in most cases by combinations of privileged and marginalised social identities/positions. Both approaches evidenced the existence of large inequalities in all outcomes. Some of the largest inequalities were observed by sexual orientation, followed by birth sex and cohort/generation, with sexual minorities, females, and younger people (in their teens/20s) showing worse levels. These findings exemplify the multifaceted way in which mental (ill) health inequalities are socially patterned [5].

From a methodological standpoint, our study showcases some of the desirable features of MAIHDA models to the quantitative analysis of inequalities from an intersectional perspective. All intersections (multiply advantaged and disadvantaged, as well as all combinations in between) were included and voiced in the “socio-demographic mapping” [14], which prevented reinforcing the idea of reference categories as the “standard” [16, 17]. Combinations of privileged and marginalised identities were among those with the largest positive and negative intersectional effects in the two MAIHDA modelling strategies used, highlighting how inequalities are not limited to groups with multiply advantaged or disadvantaged positions, and that they may also be contextually contingent [8, 9]. Importantly, the lack of evidence of significant or large intersectional effects, regardless of the quantitative approach used, does not rule out the existence of different intersectional lived experiences [25], as they may not necessarily reflect upon differences in the outcomes under study. Using MAIHDA models also helped us to further embrace intersectional complexity by acknowledging the existence of heterogeneity not only between but also within intersections [11]. Discriminatory accuracy levels were similar or larger than those found in most applications of MAIHDA (where VPC_intercepts-only_ or ICC tend to be < 0.05 [22]), and generally larger for anxiety and depressive symptomatology than for loneliness and life satisfaction. These varied across stratifications using different SEP indicators, suggesting that the experiences attached to these SEP indicators may have different impacts across different outcomes.

From a substantive standpoint, our study covers a gap in the knowledge about population mental health inequalities during the pandemic from an intersectional perspective [29], and particularly among young adults who, according to previous evidence [35, 37, 38, 41, 42], have been most adversely affected by the pandemic. Women, young adults, and those in more disadvantaged socioeconomic positions had worse mental health at the time of data collection (February/March 2021, during the third UK nationwide lockdown). These results exemplify the structural, up-stream, fundamental causes (e.g., sexism, classism, heteronormativity) of inequality, leading to differential exposures to experiences such as discrimination and stigma [2, 5]. The mental health inequalities by sexual orientation observed in our study are a grim example of this, extending recent evidence from earlier data collection time points in MCS [61–63] and showing that these inequalities are large and, in most cases, cut across different mental health outcomes, cohorts/generations, sexes, racial/ethnic groups, and socioeconomic levels. Inequalities by sexual orientation may be explained by the differential exposure to experiences such as reduced peer support availability and increased exposure to discrimination or familial rejection (e.g., increased time spent in family contexts that may have been unsupportive), as well as poorer pre-pandemic health and mental health [64–67]. Although disproportionate COVID-19 infection and mortality rates in minoritised racial/ethnic groups have been documented [68], we did not find consistent evidence of mental health inequalities by racial/ethnic groups. The weighted results suggested that some racial/ethnic groups (particularly the Mixed and “Other” ethnicity groups) had worse levels in multiple outcomes. This goes in line with previous research suggesting larger distress levels during the pandemic in the UK general adult population using similar groups [42], and adds to the mixed evidence on loneliness, where coarser ethnicity/racial groups (White vs non-White) have been used [30, 69]. Estimates of the additive/main effects associated with different racial/ethnic groups were the most variable across the two MAIHDA modelling approaches used (unweighted vs weighted), suggesting a larger bias of non-response in these estimates.

### Limitations and future directions

This is, to our knowledge, the first study to document population inequalities in different mental health outcomes during the pandemic using MAIHDA models, with the already mentioned advantages of doing so relative to other more traditional approaches. These results must be interpreted considering several limitations. Despite the diversity in the cohorts, the number of participants from racially/ethnically minoritised and sexual minority groups was small. This had multiple implications for our study. We had to group some of the least frequent categories (e.g., sexual minorities and ethnic groups), lumping together people with different experiences, perspectives, histories, cultures, and complexity in relation to experiences of marginalisation and oppression, thus obscuring (and increasing) the sources of heterogeneity within intersections. Even after grouping those categories, some of the intersections had none or very few observations, which prevented us from mapping the missing intersections and likely limited our ability to detect intersectional effects at some of these intersections which may be at risk. The small sample size at some intersections may also explain some of the differences across the MAIHDA models and the multiple regression fixed-effects models: MAIHDA models introduce a correction (shrinkage) to adjust the estimates of intersections by their precision (based on their size) [22]. This has been documented to result in smaller number of statistically significant intersectional effects compared to fixed-effects approaches [21, 25], which do not include this correction thus potentially resulting in significant interaction effects based on very few observations. Surveys designed to ensure sufficient sizes at all intersections to be studied are needed to overcome these limitations [12, 18].

Second, the small number of indicators in our outcome measures contributed towards measurement error, thus artificially increasing their heterogeneity. This also prevented us from exploring the equivalence of the measures across the intersections under study. Future research using longer versions of these and other instruments may result in more reliable/accurate outcome measurements, while also enabling testing measurement equivalence using suitable methods [70].

Third, due to differential non-response across groups [47], the results from the MCMC analyses, which permit assessing the statistical significance of the intersectional effects, may only be generalisable to the study participants. Since weighted analyses have not yet been implemented for MCMC MAIHDA models, we tried to overcome this limitation by re-estimating the MAIHDA models with ML using survey and non-response weights, at the cost of not obtaining confidence intervals for the intersectional effects. Both approaches resulted in remarkably similar main/additive effects, but both discriminatory accuracy and intersectional effects were generally larger in the weighted results. Aside the obvious need for implementation of weighted analysis in standard MAIHDA models, boostrapping conditioned on clusters defined by intersections may be a potential solution to obtain confidence intervals for the intersectional effects when using weighted ML, but methodological work beyond the scope of this paper, including formal simulations, is needed to test this approach.

Fourth, the “socio-demographic mapping” provided is only applicable to the social identities/positions under study: we were, for instance, unable to examine mental health of transgender and gender diverse groups despite evidence suggesting they were also disproportionately adversely affected by the pandemic [71, 72].

Finally, the cross-sectional design provides a snapshot of the inequalities at one time-point, coinciding with a lockdown period. This may not be generalisable to other pandemic periods, as longitudinal UK-based evidence shows that levels of different mental health measures changed over the pandemic course [30, 31, 35–43]. Future studies may cover this gap by extending the MAIHDA modelling approach to longitudinal designs.

## Conclusions

We have illustrated how quantitative methods can be used to study population intersectional mental health inequalities. Our study evidences large mental health inequalities across (and within) intersectional strata in the population. Large proportions of these inequalities can be accounted for by the main/additive effects of the variables used to define those intersections (cohort/generation, birth sex, racial/ethnic group, sexual orientation, and SEP), with particularly large inequalities by sexual orientation across all studied outcomes. Our analyses also suggest that some of those inequalities were not strictly equivalent across all intersections and support the notion (and the importance of acknowledging) that inequalities are not limited to groups with multiply advantaged or disadvantaged identities/positions. The large gaps found by sexual orientation support and extend existing evidence that sexual minority groups were disproportionately affected by the pandemic. Interventions to provide support, along with further research aimed at understanding intersectional experiences of discrimination across different racial/ethnic groups and socioeconomic levels, are crucial.

## Supplementary Information

Below is the link to the electronic supplementary material.

Supplementary file1 (PDF 2046 KB)

## Data Availability

Deidentified data and documentation on Next Steps [SN 2000030] and Millennium Cohort Study [SN 2000031] are available from the UK Data Service: https://ukdataservice.ac.uk/.

## References

[1] Evans CR (2019). Modeling the intersectionality of processes in the social production of health inequalities. Soc Sci Med.

[2] Hatzenbuehler ML, Phelan JC, Link BG (2013). Stigma as a fundamental cause of population health inequalities. Am J Public Health.

[3] May VM (2015). Pursuing intersectionality unsettling dominant imaginaries. Contemporary sociological perspectives.

[4] Link BG, Garcia SJ (2021). Diversions: how the underrepresentation of research on advantaged groups leaves explanations for health inequalities incomplete. J Health Soc Behav.

[5] Link BG, Phelan J (1995). Social conditions as fundamental causes of disease. J Health Soc Behav.

[6] Tuck E (2009). Suspending damage: a letter to communities. Harv Educ Rev.

[7] Butler J (2016). Rethinking vulnerability and resistance. Vulnerability in resistance.

[8] McLeod JD (2015). Why and how inequality matters. J Health Soc Behav.

[9] Rhead RD, Woodhead C, Ahmad G, Das-Munshi J, McManus S, Hatch SL (2022). A comparison of single and intersectional social identities associated with discrimination and mental health service use: data from the 2014 adult psychiatric morbidity survey in England. Soc Psychiatr Psychiatr Epidemiol.

[10] McLeod JD, Lively KJ, Avison WR, McLeod JD, Pescosolido BA (2007). Social psychology and stress research. Mental health, social mirror.

[11] Hancock A-M (2013). Empirical intersectionality: a tale of two approaches. UC Irvine Law Review.

[12] Bowleg L (2008). When Black + Lesbian + Woman ≠ Black Lesbian Woman: the methodological challenges of qualitative and quantitative intersectionality research. Sex Roles.

[13] Bauer GR, Scheim AI (2019). Methods for analytic intercategorical intersectionality in quantitative research: discrimination as a mediator of health inequalities. Soc Sci Med.

[14] Bauer GR (2014). Incorporating intersectionality theory into population health research methodology: challenges and the potential to advance health equity. Soc Sci Med.

[15] McCall L (2005). The complexity of intersectionality. Signs.

[16] Choo HY, Ferree MM (2010). Practicing intersectionality in sociological research: a critical analysis of inclusions, interactions, and institutions in the study of inequalities. Sociol Theory.

[17] Evans CR, Williams DR, Onnela JP, Subramanian SV (2018). A multilevel approach to modeling health inequalities at the intersection of multiple social identities. Soc Sci Med.

[18] Bowleg L, Bauer G (2016). Invited reflection: quantifying intersectionality. Psychol Women Q.

[19] Bauer GR, Churchill SM, Mahendran M, Walwyn C, Lizotte D, Villa-Rueda AA (2021). Intersectionality in quantitative research: a systematic review of its emergence and applications of theory and methods. SSM Popul Health.

[20] Bauer GR, Scheim AI (2019). Advancing quantitative intersectionality research methods: Intracategorical and intercategorical approaches to shared and differential constructs. Soc Sci Med.

[21] Evans CR (2019). Adding interactions to models of intersectional health inequalities: comparing multilevel and conventional methods. Soc Sci Med.

[22] Evans CR, Leckie G, Merlo J (2020). Multilevel versus single-level regression for the analysis of multilevel information: the case of quantitative intersectional analysis. Soc Sci Med.

[23] Merlo J (2018). Multilevel analysis of individual heterogeneity and discriminatory accuracy (MAIHDA) within an intersectional framework. Soc Sci Med.

[24] Bauer GR, Mahendran M, Walwyn C, Shokoohi M (2022). Latent variable and clustering methods in intersectionality research: systematic review of methods applications. Soc Psychiatr Psychiatr Epidemiol.

[25] Evans CR, Erickson N (2019). Intersectionality and depression in adolescence and early adulthood: a MAIHDA analysis of the national longitudinal study of adolescent to adult health, 1995–2008. Soc Sci Med.

[26] Glymour MM, Rudolph KE (2016). Causal inference challenges in social epidemiology: Bias, specificity, and imagination. Soc Sci Med.

[27] Marmot M, Allen J, Goldblatt P, Herd E, Morrison J (2020). Build back fairer: the COVID-19 Marmot review the pandemic, socioeconomic and health inequalities in England.

[28] Bambra C, Riordan R, Ford J, Matthews F (2020). The COVID-19 pandemic and health inequalities. J Epidemiol Commun Health.

[29] Gibson B, Schneider J, Talamonti D, Forshaw M (2021). The Impact of inequality on mental health outcomes during the COVID-19 pandemic: a systematic review. Can Psychol-Psychologie Canadienne.

[30] Bu F, Steptoe A, Fancourt D (2020). Who is lonely in lockdown? cross-cohort analyses of predictors of loneliness before and during the COVID-19 pandemic. Public Health.

[31] Creese B, Khan Z, Henley W, O’Dwyer S, Corbett A, Vasconcelos Da Silva M, Mills K, Wright N, Testad I, Aarsland D, Ballard C (2021). Loneliness, physical activity, and mental health during COVID-19: a longitudinal analysis of depression and anxiety in adults over the age of 50 between 2015 and 2020. Int Psychogeriatr.

[32] Creswell C, Shum A, Pearcey S, Skripkauskaite S, Patalay P, Waite P (2021). Young people’s mental health during the COVID-19 pandemic. Lancet Child Adolesc Health.

[33] Ellwardt L, Prag P (2021). Heterogeneous mental health development during the COVID-19 pandemic in the United Kingdom. Sci Rep.

[34] Fancourt D, Steptoe A, Bu F (2021). Trajectories of anxiety and depressive symptoms during enforced isolation due to COVID-19 in England: a longitudinal observational study. Lancet Psychiatr.

[35] Kwong ASF, Pearson RM, Adams MJ, Northstone K, Tilling K, Smith D, Fawns-Ritchie C, Bould H, Warne N, Zammit S, Gunnell DJ, Moran PA, Micali N, Reichenberg A, Hickman M, Rai D, Haworth S, Campbell A, Altschul D, Flaig R, McIntosh AM, Lawlor DA, Porteous D, Timpson NJ (2021). Mental health before and during the COVID-19 pandemic in two longitudinal UK population cohorts. Br J Psychiatr.

[36] Moreno-Agostino D, Fisher HL, Goodman A, Hatch SL, Morgan C, Richards M, Das-Munshi J, Ploubidis GB. Long-term psychological distress trajectories and the COVID-19 pandemic in three British birth cohorts: a multi-cohort study. PLOS Medicine. 10.1371/journal.pmed.1004145**(in press)** 10.1371/journal.pmed.1004145 PMC10072377 37014820

[37] Moreno-Agostino D, Fisher HL, Hatch SL, Morgan C, Ploubidis GB, Das-Munshi J (2022). Generational, sex, and socioeconomic inequalities in mental and social wellbeing during the COVID-19 pandemic: prospective longitudinal observational study of five UK cohorts. Psychological Medicine.

[38] Niedzwiedz CL, Green MJ, Benzeval M, Campbell D, Craig P, Demou E, Leyland A, Pearce A, Thomson R, Whitley E, Katikireddi SV (2021). Mental health and health behaviours before and during the initial phase of the COVID-19 lockdown: longitudinal analyses of the UK household longitudinal study. J Epidemiol Commun Health.

[39] O'Connor RC, Wetherall K, Cleare S, McClelland H, Melson AJ, Niedzwiedz CL, O'Carroll RE, O’Connor DB, Platt S, Scowcroft E, Watson B, Zortea T, Ferguson E, Robb KA (2021). Mental health and well-being during the COVID-19 pandemic: longitudinal analyses of adults in the UK COVID-19 mental health & wellbeing study. Br J Psychiatr.

[40] Patel K, Robertson E, Kwong ASF, Griffith GJ, Willan K, Green MJ, Di Gessa G, Huggins CF, McElroy E, Thompson EJ, Maddock J, Niedzwiedz CL, Henderson M, Richards M, Steptoe A, Ploubidis GB, Moltrecht B, Booth C, Fitzsimons E, Silverwood R, Patalay P, Porteous D, Katikireddi SV (2022). Psychological distress before and during the COVID-19 pandemic among adults in the United Kingdom based on coordinated analyses of 11 longitudinal studies. JAMA Netw Open.

[41] Pierce M, Hope H, Ford T, Hatch S, Hotopf M, John A, Kontopantelis E, Webb R, Wessely S, McManus S, Abel KM (2020). Mental health before and during the COVID-19 pandemic: a longitudinal probability sample survey of the UK population. Lancet Psychiatr.

[42] Pierce M, McManus S, Hope H, Hotopf M, Ford T, Hatch SL, John A, Kontopantelis E, Webb RT, Wessely S, Abel KM (2021). Mental health responses to the COVID-19 pandemic: a latent class trajectory analysis using longitudinal UK data. Lancet Psychiatr.

[43] Zaninotto P, Iob E, Demakakos P, Steptoe A (2021). Immediate and longer-term changes in the mental health and well-being of older adults in England during the COVID-19 pandemic. JAMA Psychiat.

[44] Calderwood L, Sánchez C (2016). Next steps (formerly known as the longitudinal study of young people in England). Open Health Data.

[45] Connelly R, Platt L (2014). Cohort profile: UK millennium cohort study (MCS). Int J Epidemiol.

[46] Institute for Government (2021) Timeline of UK government coronavirus lockdowns. https://www.instituteforgovernment.org.uk/charts/uk-government-coronavirus-lockdowns. Accessed 4 Nov 2021

[47] Brown M, Goodman A, Peters A, Ploubidis GB, Sanchez A, Silverwood R, Smith K (2021). COVID-19 survey in five national longitudinal studies: Waves 1, 2 and 3 User Guide (Version 3).

[48] Kroenke K, Spitzer RL, Williams JB, Monahan PO, Lowe B (2007). Anxiety disorders in primary care: prevalence, impairment, comorbidity, and detection. Ann Intern Med.

[49] Kroenke K, Spitzer RL, Williams JB (2003). The patient health questionnaire-2: validity of a two-item depression screener. Med Care.

[50] Hughes ME, Waite LJ, Hawkley LC, Cacioppo JT (2004). A short scale for measuring loneliness in large surveys: results from two population-based studies. Res Aging.

[51] Office for National Statistics (2018) Personal well-being user guidance. https://www.ons.gov.uk/peoplepopulationandcommunity/wellbeing/methodologies/personalwellbeingsurveyuserguide. Accessed 28 Sep 2021

[52] Office for National Statistics (2022) Ethnic group, national identity and religion. https://www.ons.gov.uk/methodology/classificationsandstandards/measuringequality/ethnicgroupnationalidentityandreligion. Accessed 02 Feb 2022

[53] Noble S, McLennan D, Noble M, Plunkett E, Gutacker N, Silk M, Wright G (2019). The English indices of deprivation 2019. Research report.

[54] Dodgeon B, Morris T, Crawford C, Parsons S, Vignoles A, Oldfield Z, O’Neill D (2011). CLOSER work package 2: Harmonised socio-economic measures user guide (revised).

[55] Browne WJ (2012). MCMC estimation in MLwiN, v2.26. Centre for multilevel modelling.

[56] Rasbash J, Charlton C, Browne WJ, Healy M, Cameron B (2009). MLwiN Version 2.1. Centre for multilevel modelling.

[57] Leckie G, Charlton C (2013). runmlwin: a program to run the MLwiN multilevel modeling software from within stata. J Stat Softw.

[58] StataCorp (2021). Stata statistical software: release 17.

[59] Axelsson Fisk S, Mulinari S, Wemrell M, Leckie G, Perez Vicente R, Merlo J (2018). Chronic obstructive pulmonary disease in Sweden: an intersectional multilevel analysis of individual heterogeneity and discriminatory accuracy. SSM Popul Health.

[60] Persmark A, Wemrell M, Zettermark S, Leckie G, Subramanian SV, Merlo J (2019). Precision public health: Mapping socioeconomic disparities in opioid dispensations at Swedish pharmacies by multilevel analysis of individual heterogeneity and discriminatory accuracy (MAIHDA). PLoS ONE.

[61] Becares L, Kneale D (2022). Inequalities in mental health, self-rated health, and social support among sexual minority young adults during the COVID-19 pandemic: analyses from the UK millennium cohort study. Soc Psychiatr Psychiatr Epidemiol.

[62] Amos R, Manalastas EJ, White R, Bos H, Patalay P (2020). Mental health, social adversity, and health-related outcomes in sexual minority adolescents: a contemporary national cohort study. Lancet Child Adolesc Health.

[63] Patalay P, Fitzsimons E (2021). Psychological distress, self-harm and attempted suicide in UK 17-year olds: prevalence and sociodemographic inequalities. Br J Psychiatry.

[64] Ormiston CK, Williams F (2022). LGBTQ youth mental health during COVID-19: unmet needs in public health and policy. Lancet.

[65] Salerno JP, Devadas J, Pease M, Nketia B, Fish JN (2020). Sexual and gender minority stress amid the COVID-19 pandemic: implications for LGBTQ young persons’ mental health and well-being. Public Health Rep.

[66] Salerno JP, Doan L, Sayer LC, Drotning KJ, Rinderknecht RG, Fish JN (2021). Changes in mental health and well-being are associated with living arrangements with parents during COVID-19 among sexual minority young persons in the US. Psychol Sex Orientat Gender Divers.

[67] Fish JN, McInroy LB, Paceley MS, Williams ND, Henderson S, Levine DS, Edsall RN (2020). “I’m kinda stuck at home with unsupportive parents right now”: LGBTQ youths’ experiences with COVID-19 and the importance of online support. J Adolesc Health.

[68] Katikireddi SV, Hainey KJ, Beale S (2021). The impact of COVID-19 on different population subgroups: ethnic, gender and age-related disadvantage. J R Coll Physicians Edinb.

[69] Bu F, Steptoe A, Fancourt D (2020). Loneliness during a strict lockdown: Trajectories and predictors during the COVID-19 pandemic in 38,217 United Kingdom adults. Soc Sci Med.

[70] Muthen B, Asparouhov T (2018). Recent methods for the study of measurement invariance with many groups: alignment and random effects. Sociol Methods Res.

[71] Hawke LD, Hayes E, Darnay K, Henderson J (2021). Mental health among transgender and gender diverse youth: an exploration of effects during the COVID-19 pandemic. Psychol Sex Orientat Gend Divers.

[72] Wang Y, Pan B, Liu Y, Wilson A, Ou J, Chen R (2020). Health care and mental health challenges for transgender individuals during the COVID-19 pandemic. Lancet Diabetes Endocrinol.

