# Supplementary material for: A quantitative approach to the intersectional study of mental health inequalities during the COVID-19 pandemic in UK young adults
Source: Soc Psychiatry Psychiatr Epidemiol. 2023 Jan 24;59(3):417–29. doi: 10.1007/s00127-023-02424-0 (PMC9872068; doi:10.1007/s00127-023-02424-0)
Supplement: Supplementary file 1 — Supplementary file1 (PDF 2046 KB) [file 127_2023_2424_MOESM1_ESM.pdf]

A quantitative approach to the intersectional study of mental health inequalities during the COVID-19 pandemic in UK young adults

Supplementary Material

Table of Contents

Appendix S1. Assessment of mismatch/bias between Bayesian Markov Chain Monte Carlo (MCMC) and maximum likelihood (ML) MAIHDA models 3

Appendix S2. Additional details on sensitivity checks and comparison with fixed-effects approach. 9

Table S1. Distribution of variables adopted to define the intersectional strata in the overall sample and by cohort. 10

Table S2. Number of strata with at least one observation and missing strata under the different stratifications. 11

Table S3. Number of observations by intersectional strata. 12

Table S4. Fixed- and random-effects from the main models. Markov Chain Monte Carlo (MCMC) estimation, unweighted results. 15

Table S5. Fixed- and random-effects from the main models. Maximum likelihood estimation, weighted (survey design and non-response) results. 18

Table S6. Results from the fixed-effects multiple regression approach using stratification 40 (cohort * birth sex * ethnic group * sexual orientation). 21

Table S7. Results from the fixed-effects multiple regression approach using stratification 80a (cohort * birth sex * ethnic group * sexual orientation * residential Index of Multiple Deprivation rank). 24

Table S8. Results from the fixed-effects multiple regression approach using stratification 80b (cohort * birth sex * ethnic group * sexual orientation * housing tenure). 29

Table S9. Results from the fixed-effects multiple regression approach using stratification 80c (cohort * birth sex * ethnic group * sexual orientation * childhood social class). 34

Figure S1. Stratum-specific residual values and 95% credible intervals for each outcome using 40 strata. Markov Chain Monte Carlo (MCMC) estimation, unweighted results. 39

Figure S2. Stratum-specific predicted values and 95% credible intervals for each outcome using 40 strata. Markov Chain Monte Carlo (MCMC) estimation, unweighted results. 40

Figure S3. Stratum-specific residual values for each outcome using 40 strata. Maximum likelihood estimation, weighted (survey design and non-response) results. 41

Figure S4. Stratum-specific predicted values for each outcome using 40 strata. Maximum likelihood estimation, weighted (survey design and non-response) results. 42

Figure S5. Anxiety and depressive symptomatology predicted values of each intersectional strata. Markov Chain Monte Carlo (MCMC) estimation, unweighted results. 43

Figure S6. Loneliness and life satisfaction predicted values of each intersectional strata. Markov Chain Monte Carlo (MCMC) estimation, unweighted results. 44

Figure S7. Anxiety and depressive symptomatology predicted values of each intersectional strata. Maximum likelihood estimation, weighted (survey design and non-response) results. 45

Figure S8. Loneliness and life satisfaction predicted values of each intersectional strata. Maximum likelihood estimation, weighted (survey design and non-response) results. 46

Figure S9. Anxiety and depressive symptomatology residual values (intersectional effects) and 95% credible intervals of each intersectional stratum using housing tenure as the indicator of socioeconomic position. Markov Chain Monte Carlo (MCMC) estimation, unweighted results. 47

Figure S10. Loneliness and life satisfaction residual values (intersectional effects) and 95% credible intervals of each intersectional stratum using housing tenure as the indicator of socioeconomic position. Markov Chain Monte Carlo (MCMC) estimation, unweighted results. 48

Figure S11. Anxiety and depressive symptomatology residual values (intersectional effects) and 95% credible intervals of each intersectional stratum using parental social class during childhood as the indicator of socioeconomic position. Markov Chain Monte Carlo (MCMC) estimation, unweighted results. 49

Figure S12. Loneliness and life satisfaction residual values (intersectional effects) and 95% credible intervals of each intersectional stratum using parental social class during childhood as the indicator of socioeconomic position. Markov Chain Monte Carlo (MCMC) estimation, unweighted results. 50

Figure S13. Anxiety and depressive symptomatology residual values (intersectional effects) using residential Index of Multiple Deprivation (IMD) rank as the indicator of socioeconomic position. Maximum likelihood estimation, weighted (survey design and non-response) results. 51

Figure S14. Loneliness and life satisfaction residual values (intersectional effects) using residential Index of Multiple Deprivation (IMD) rank as the indicator of socioeconomic position. Maximum likelihood estimation, weighted (survey design and non-response) results. 52

Figure S15. Anxiety and depressive symptomatology residual values (intersectional effects) using housing tenure as the indicator of socioeconomic position. Maximum likelihood estimation, weighted (survey design and non-response) results. 53

Figure S16. Loneliness and life satisfaction residual values (intersectional effects) using housing tenure as the indicator of socioeconomic position. Maximum likelihood estimation, weighted (survey design and non-response) results. 54

Figure S17. Anxiety and depressive symptomatology residual values (intersectional effects) using parental social class during childhood as the indicator of socioeconomic position. Maximum likelihood estimation, weighted (survey design and non-response) results. 55

Figure S18. Loneliness and life satisfaction residual values (intersectional effects) using parental social class during childhood as the indicator of socioeconomic position. Maximum likelihood estimation, weighted (survey design and non-response) results. 56

Supplementary references 57

Appendix S1. Assessment of mismatch/bias between Bayesian Markov Chain Monte Carlo (MCMC) and maximum likelihood (ML) MAIHDA models

Due to the complexity of the designs of Next Steps and Millennium Cohort Study, and the differential non-response to the COVID-19 Survey used in this study [1], we aimed to conduct the analyses using the corresponding survey and non-response weights. Non-response weights were derived for all participants in the COVID-19 Survey following the Centre for Longitudinal Studies (CLS) Missing Data Strategy [2], and have been found to be effective at restoring sample representativeness [1,3].

However, the use of weights under the 'standard' analytical approach to estimating MAIHDA models (i.e., Bayesian Markov Chain Monte Carlo (MCMC) estimation, see [4-7]) has not yet been implemented. Therefore, in order to understand the extent of potential bias to which not accounting for the survey design and survey non-response may lead, we conducted an additional set of MAIHDA models using Maximum Likelihood (ML) estimation, which in turn does not provide (an approximation to) significance testing for the intersectional effects.

We first explored the degree of mismatch across the levels predicted at each intersectional stratum between unweighted MCMC and ML MAIHDA approaches across stratifications (40, 80a, 80b, 80c) and outcomes (anxiety symptomatology, depressive symptomatology, loneliness, and life satisfaction). The degree of mismatch across these two approaches was negligible, as shown evidenced in **Appendix S1.1** (note the narrow X-axis, with most cases being very close to zero, which signifies no difference across approaches).

We then explored the degree of mismatch across ML MAIHDA approaches using no weights, using survey weights, and using survey and non-response weights combined. Plots showing the mismatch across the different stratifications are available in **Appendix S1.2** (stratification 40), **Appendix S1.3** (stratification 80a), **Appendix S1.4** (stratification 80b), and **Appendix S1.5** (stratification 80c). Note that the X-axes in these graphs have been expanded (further under stratification 80c) to account for the larger differences/mismatch in some cases.

The exploration suggested that survey non-response was introducing bias in the estimation, thus hindering generalisability beyond the survey respondents of the results from models not accounting for differential non-response.

Appendix S1.1. Mismatch in stratum-specific predicted levels across unweighted Markov Chain Monte Carlo (MCMC) and Maximum Likelihood (ML) estimations.

GAD-2: 2-item Generalised Anxiety Disorder questionnaire; ONS LS: Office for National Statistics life satisfaction question; PHQ-2: 2-item Patient Health Questionnaire depression questionnaire; UCLA-3: 3-item University of California Los Angeles loneliness scale.

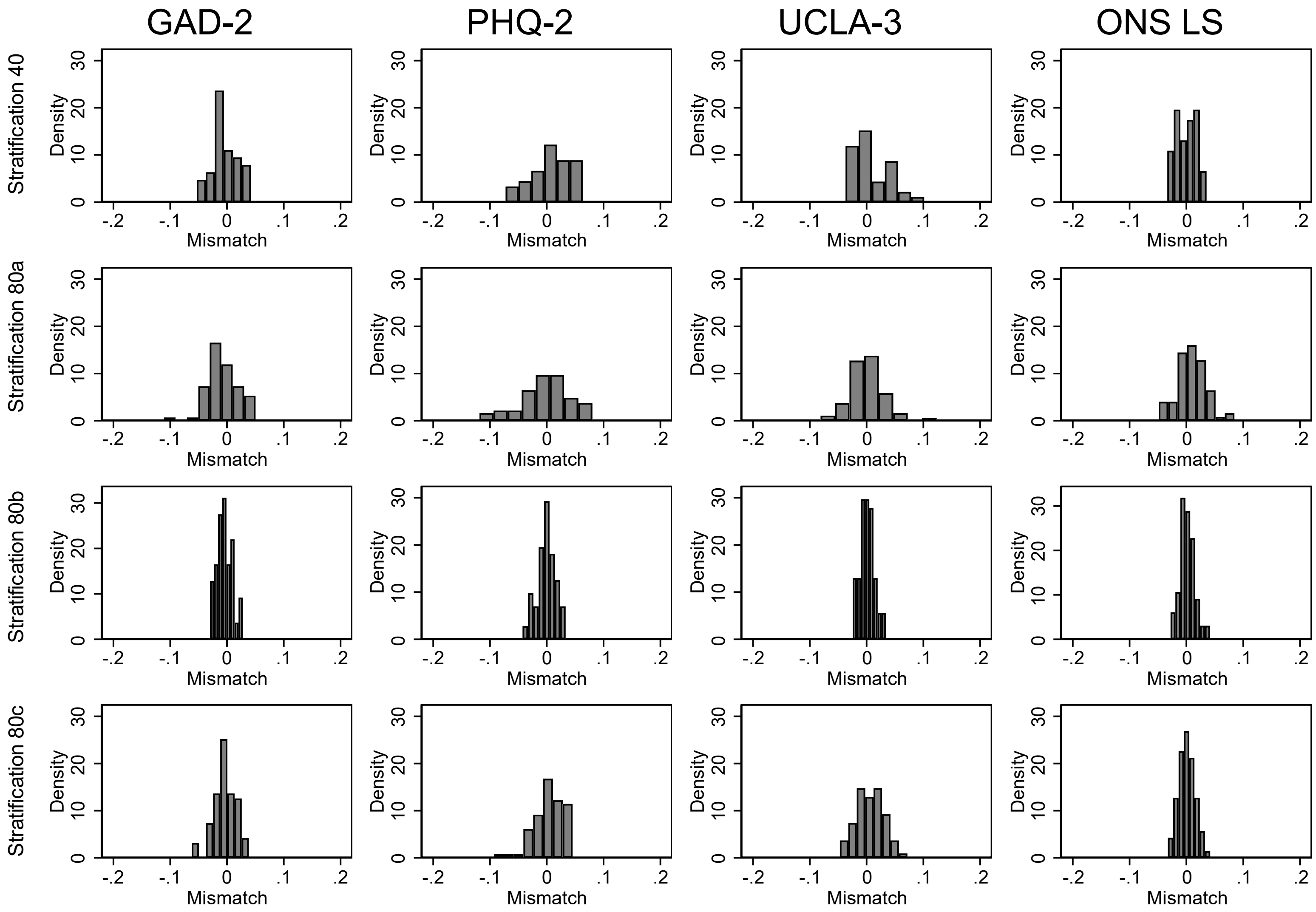

Appendix S1.2. Mismatch in stratum-specific predicted levels across Maximum Likelihood (ML) estimation approaches using stratification 40.

GAD-2: 2-item Generalised Anxiety Disorder questionnaire; ONS LS: Office for National Statistics life satisfaction question; PHQ-2: 2-item Patient Health Questionnaire depression questionnaire; UCLA-3: 3-item University of California Los Angeles loneliness scale.

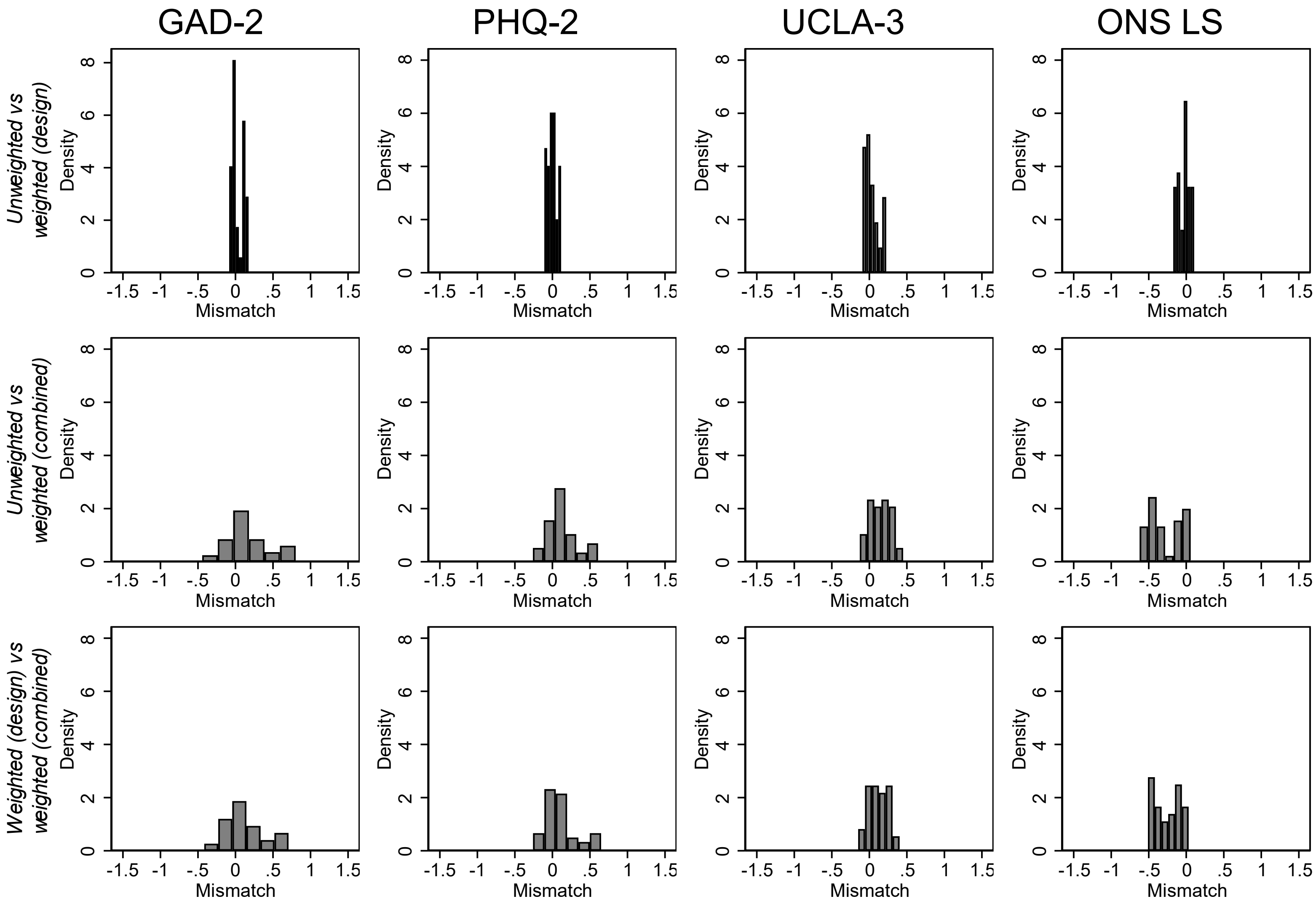

Appendix S1.3. Mismatch in stratum-specific predicted levels across Maximum Likelihood (ML) estimation approaches using stratification 80a.

GAD-2: 2-item Generalised Anxiety Disorder questionnaire; ONS LS: Office for National Statistics life satisfaction question; PHQ-2: 2-item Patient Health Questionnaire depression questionnaire; UCLA-3: 3-item University of California Los Angeles loneliness scale.

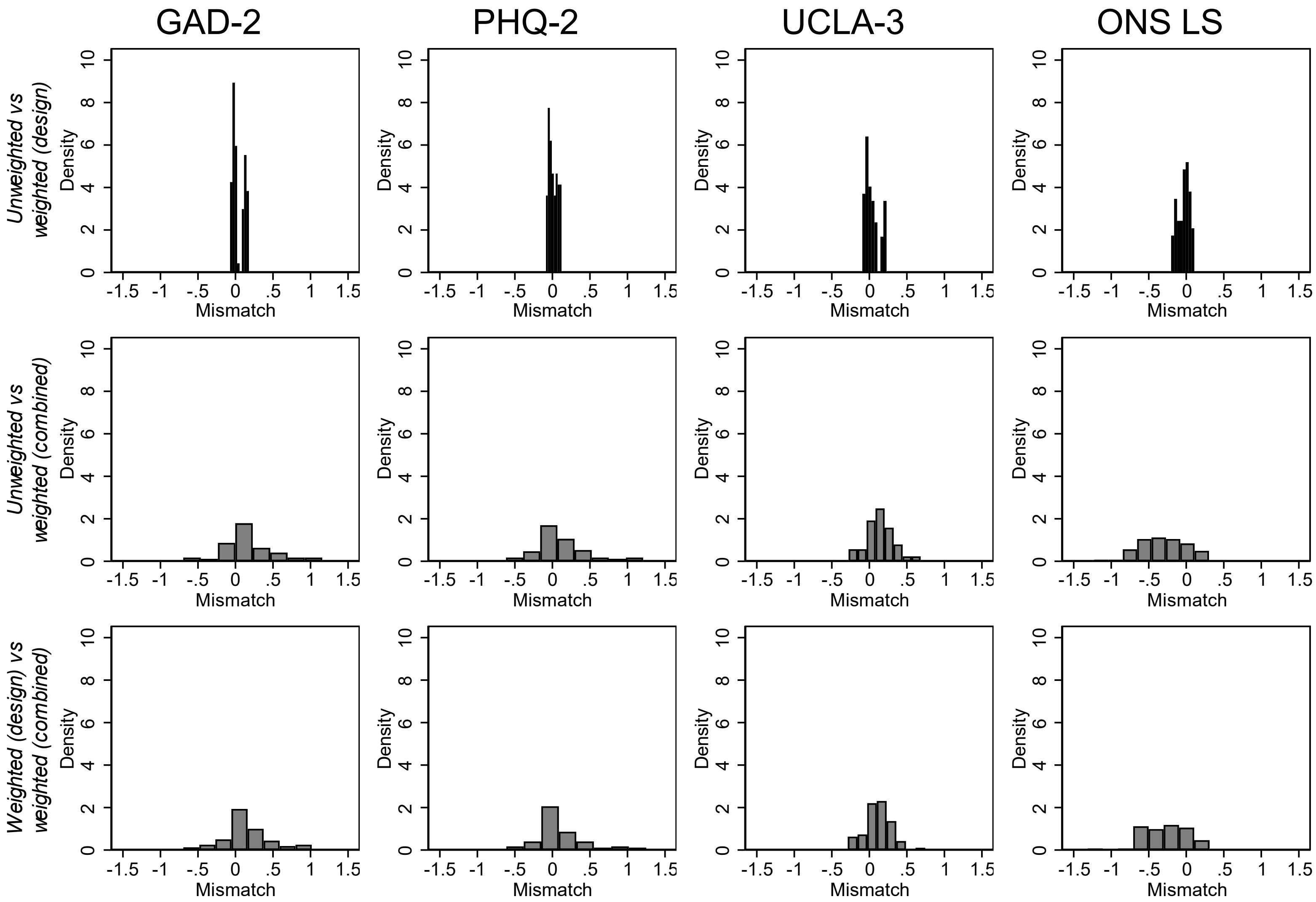

Appendix S1.4. Mismatch in stratum-specific predicted levels across Maximum Likelihood (ML) estimation approaches using stratification 80b.

GAD-2: 2-item Generalised Anxiety Disorder questionnaire; ONS LS: Office for National Statistics life satisfaction question; PHQ-2: 2-item Patient Health Questionnaire depression questionnaire; UCLA-3: 3-item University of California Los Angeles loneliness scale.

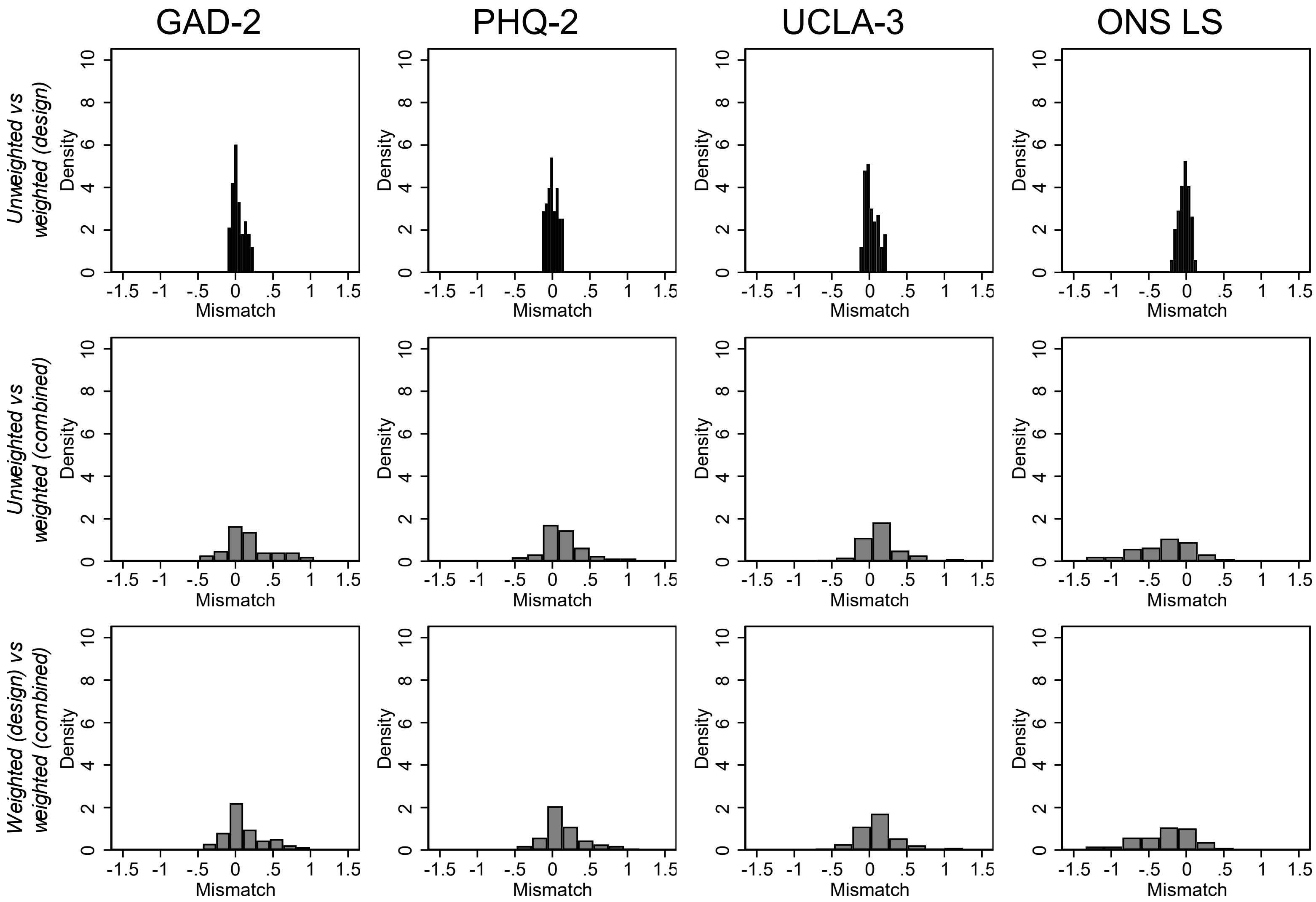

Appendix S1.5. Mismatch in stratum-specific predicted levels across Maximum Likelihood (ML) estimation approaches using stratification 80c.

GAD-2: 2-item Generalised Anxiety Disorder questionnaire; ONS LS: Office for National Statistics life satisfaction question; PHQ-2: 2-item Patient Health Questionnaire depression questionnaire; UCLA-3: 3-item University of California Los Angeles loneliness scale.

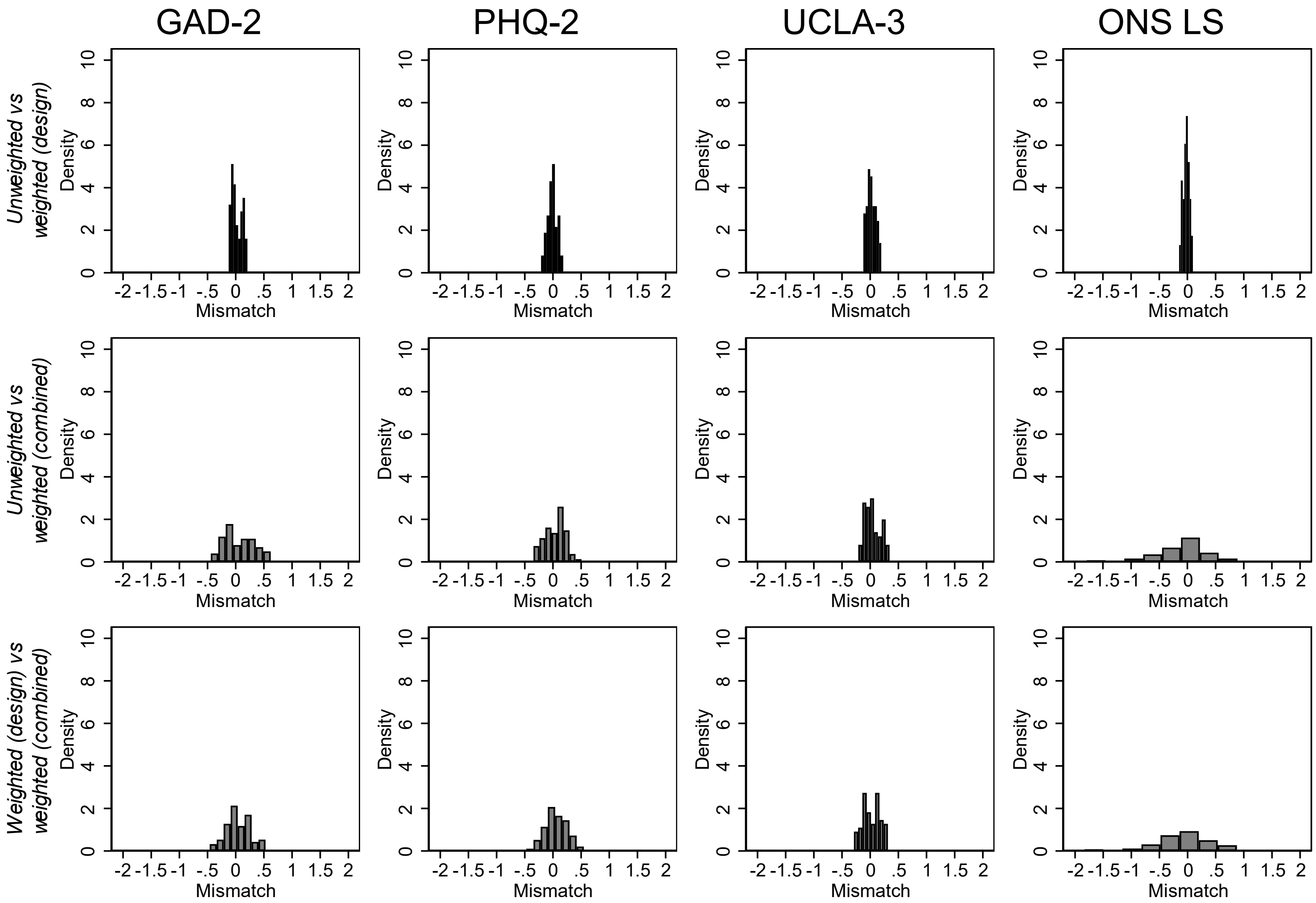

Appendix S2. Additional details on sensitivity checks and comparison with fixed-effects approach.

We used a fixed-effects multiple regression approach including interaction effects across all the variables adopted to define the intersections. This approach has several limitations compared to MAIHDA models when it comes to studying inequalities from an intersectional perspective, including the necessary use of reference categories and scalability and interpretability issues when multiple intersecting social identities/positions are included [4,8]. However, it remains a very widely used approach [9], so we performed this additional set of analysis for comparison purposes.

Table S1. Distribution of variables adopted to define the intersectional strata in the overall sample and by cohort.

	**Next Steps (1990)**	**MCS (2000/2002)**	**Total**
	N=4,167	N=4,421	N=8,588
**Birth sex**			
Female	2,599 (62.4%)	2,674 (60.5%)	5,273 (61.4%)
Male	1,568 (37.6%)	1,747 (39.5%)	3,315 (38.6%)
**Ethnicity**			
White (all)	3,062 (73.5%)	3,588 (81.2%)	6,650 (77.4%)
Mixed	183 (4.4%)	200 (4.5%)	383 (4.5%)
South Asian (Bangladeshi / Indian / Pakistani)	640 (15.4%)	413 (9.3%)	1,053 (12.3%)
Black African / Black Caribbean / Black British	178 (4.3%)	115 (2.6%)	293 (3.4%)
Other	104 (2.5%)	101 (2.3%)	205 (2.4%)
Missing	*0 (0.0%)*	*4 (0.1%)*	*4 (0.0%)*
**Sexual orientation**			
Heterosexual	3,774 (90.6%)	3,691 (83.5%)	7,465 (86.9%)
Sexual minority (homosexual, bisexual, other)	263 (6.3%)	559 (12.6%)	822 (9.6%)
Missing	*130 (3.1%)*	*171 (3.9%)*	*301 (3.5%)*
**Residential IMD rank**			
Higher 50% within-country IMD rank (more deprived)	2,164 (51.9%)	2,069 (46.8%)	4,233 (49.3%)
Lower 50% within-country IMD rank (less deprived)	1,948 (46.7%)	2,332 (52.7%)	4,280 (49.8%)
Missing	*55 (1.3%)*	*20 (0.5%)*	*75 (0.9%)*
**Housing tenure**			
Not homeowner/part owner	1,758 (42.2%)	2,293 (51.9%)	4,051 (47.2%)
Homeowner/part owner	2,182 (52.4%)	1,578 (35.7%)	3,760 (43.8%)
Missing	*227 (5.4%)*	*550 (12.4%)*	*777 (9.0%)*
**Parental social class during childhood (age 11/14)**			
Disadvantaged parental social class	1,396 (33.5%)	788 (17.8%)	2,184 (25.4%)
Advantaged parental social class	2,473 (59.3%)	2,915 (65.9%)	5,388 (62.7%)
Missing	*298 (7.2%)*	*718 (16.2%)*	*1,016 (11.8%)*

Note. Unweighted results. IMD: within-country Index of Multiple Deprivation; MCS: Millennium Cohort Study. “Other” ethnicity corresponds to all reported ethnicities not included in the other categories.

Table S2. Number of strata with at least one observation and missing strata under the different stratifications.

Outcome	Stratification	Valid strata	Missing strata
**GAD-2 (anxiety symptomatology)**	40	40	None
	80a	75	10211, 10410, 11311, 20510, 21411
	80b	76	11511, 20411, 20510, 21411
	80c	76	10210, 20411, 20510, 21410
**PHQ-2 (depressive symptomatology)**	40	40	None
	80a	75	10211, 10410, 11311, 20510, 21411
	80b	76	11511, 20411, 20510, 21411
	80c	76	10210, 20411, 20510, 21410
**UCLA-3 (feelings of loneliness)**	40	40	None
	80a	74	10211, 10410, 11311, *20410*, 20510, 21411
	80b	74	*10410*, 11511, *20410*, 20411, 20510, 21411
	80c	73	10210, *10410*, *11310*, *20410*, 20411, 20510, 21410
**ONS life satisfaction question**	40	40	None
	80a	75	10211, 10410, 11311, 20510, 21411
	80b	76	11511, 20411, 20510, 21411
	80c	76	10210, 20411, 20510, 21410

Note. GAD-2: 2-item Generalised Anxiety Disorder questionnaire; ONS: Office for National Statistics; PHQ-2: 2-item depression Patient Health Questionnaire; UCLA-3: 3-item University of California Los Angeles loneliness scale. Stratification 40 is defined by cohort * birth sex * racial/ethnic group * sexual orientation; strata 80a include stratification 40 * within-country index of multiple deprivation rank of the residential area; strata 80b include stratification 40 * housing tenure; strata 80c include stratification 40 * parental social class during childhood (age 11/14). Missing strata in each stratification are common across all outcomes except for loneliness (UCLA-3), which has additional missing strata that have been highlighted in italics.
Intersectional strata are defined by generation/cohort (first digit: 1 Next Steps / 1990, 2 Millennium Cohort Study / 2000-2002), birth sex (second digit: 0 Male, 1 Female), racial/ethnic group (third digit: 1 White, 2 Mixed, 3 South Asian, 4 Black, 5 Other), sexual orientation (fourth digit: 0 Heterosexual, 1 Sexual minority). The fifth digit corresponds to within-country Index of Multiple Deprivation rank in the stratification 80a (0 Below the median/more deprived area, 1 Above the median/less deprived area); housing tenure in stratification 80b (0 Not homeowner, 1 Homeowner / part owner); and parental social class during childhood in stratification 80c (0 Disadvantaged, 1 Advantaged).

Table S3. Number of observations by intersectional strata.

	Strata 40				Strata 80a				Strata 80b				Strata 80c			
	**GAD-2**	**PHQ-2**	**UCLA-3**	**ONS LS**	**GAD-2**	**PHQ-2**	**UCLA-3**	**ONS LS**	**GAD-2**	**PHQ-2**	**UCLA-3**	**ONS LS**	**GAD-2**	**PHQ-2**	**UCLA-3**	**ONS LS**
**10100**	1,026	1,027	1,024	1,030	467	468	466	469	417	417	416	418	307	308	308	308
**10101**					539	539	538	541	556	557	554	558	687	687	685	690
**10110**	94	94	94	94	53	53	53	53	49	49	49	49	28	28	28	28
**10111**					36	36	36	36	42	42	42	42	63	63	63	63
**10200**	50	51	51	51	26	26	26	26	24	25	25	25	16	16	16	16
**10201**					24	25	25	25	23	23	23	23	31	32	32	32
**10210**	2	2	2	2	2	2	2	2	1	1	1	1	0	0	0	0
**10211**					0	0	0	0	1	1	1	1	2	2	2	2
**10300**	219	218	220	222	158	157	160	160	76	77	77	78	116	115	116	117
**10301**					61	61	60	62	133	131	133	135	75	75	75	76
**10310**	14	14	14	14	7	7	7	7	6	6	6	6	9	9	9	9
**10311**					6	6	6	6	8	8	8	8	3	3	3	3
**10400**	46	46	44	46	33	33	32	33	25	25	24	25	12	12	11	12
**10401**					12	12	11	12	20	20	19	20	27	27	27	27
**10410**	2	2	1	2	0	0	0	0	1	1	0	1	1	1	0	1
**10411**					2	2	1	2	1	1	1	1	1	1	1	1
**10500**	24	24	24	24	13	13	13	13	12	12	12	12	11	11	11	11
**10501**					11	11	11	11	11	11	11	11	8	8	8	8
**10510**	6	6	6	6	3	3	3	3	4	4	4	4	2	2	2	2
**10511**					3	3	3	3	2	2	2	2	4	4	4	4
**11100**	1,664	1,664	1,667	1,669	751	751	753	755	638	637	639	640	497	497	497	497
**11101**					897	897	898	898	949	950	949	951	1,120	1,120	1,121	1,124
**11110**	118	118	118	118	53	53	53	53	66	66	66	66	45	45	45	45
**11111**					63	63	63	63	47	47	47	47	69	69	69	69
**11200**	114	114	114	115	66	66	66	67	64	64	64	64	34	34	34	34
**11201**					47	47	47	47	46	46	46	47	69	69	69	69
**11210**	9	9	9	9	6	6	6	6	4	4	4	4	1	1	1	1
**11211**					3	3	3	3	4	4	4	4	7	7	7	7
**11300**	346	345	346	348	244	244	244	245	151	152	151	151	158	156	157	158
**11301**					100	99	100	101	182	180	182	183	91	92	91	92
**11310**	6	7	6	7	6	7	6	7	3	4	3	4	1	1	0	1
**11311**					0	0	0	0	3	3	3	3	1	1	1	1
**11400**	102	102	102	103	79	79	79	80	54	54	54	54	30	30	30	30
**11401**					22	22	22	22	40	40	40	40	59	59	59	60
**11410**	5	5	5	5	4	4	4	4	4	4	4	4	1	1	1	1
**11411**					1	1	1	1	1	1	1	1	2	2	2	2
**11500**	63	63	63	63	39	39	39	39	37	37	37	37	24	24	24	24
**11501**					24	24	24	24	22	22	22	22	29	29	29	29

**11510**	4	4	4	4	3	3	3	3	4	4	4	4	1	1	1	1
**11511**					1	1	1	1	0	0	0	0	2	2	2	2
**20100**	1,185	1,186	1,185	1,197	459	460	457	465	609	608	608	613	178	179	180	181
**20101**					722	722	724	728	460	461	463	467	868	866	866	874
**20110**	121	121	122	123	55	55	55	55	80	80	80	80	17	17	17	17
**20111**					66	66	67	68	34	34	35	36	82	82	83	84
**20200**	61	61	62	62	36	36	36	36	26	26	27	27	6	6	6	6
**20201**					25	25	26	26	26	26	26	26	45	45	46	46
**20210**	6	6	6	6	3	3	3	3	3	3	3	3	1	1	1	1
**20211**					3	3	3	3	3	3	3	3	5	5	5	5
**20300**	144	144	144	147	109	109	108	111	53	53	55	55	40	40	38	40
**20301**					35	35	36	36	66	66	65	66	69	69	70	72
**20310**	5	5	5	5	1	1	1	1	2	2	2	2	2	2	2	2
**20311**					4	4	4	4	3	3	3	3	3	3	3	3
**20400**	43	43	43	43	34	34	34	34	27	27	27	27	8	8	8	8
**20401**					9	9	9	9	12	12	12	12	18	18	18	18
**20410**	2	2	1	2	1	1	0	1	1	1	0	1	1	1	0	1
**20411**					1	1	1	1	0	0	0	0	0	0	0	0
**20500**	31	31	29	31	20	20	18	20	11	11	10	11	7	7	7	7
**20501**					11	11	11	11	11	11	10	11	15	15	14	15
**20510**	2	2	2	2	0	0	0	0	0	0	0	0	0	0	0	0
**20511**					2	2	2	2	2	2	2	2	1	1	1	1
**21100**	1,647	1,643	1,637	1,650	678	674	672	680	868	866	857	868	269	267	264	269
**21101**					959	959	955	960	601	599	603	603	1,187	1,185	1,184	1,189
**21110**	349	349	350	349	166	166	166	165	219	219	219	218	54	54	54	54
**21111**					179	179	180	180	101	101	102	102	229	229	229	229
**21200**	90	91	91	91	52	52	52	52	55	56	56	56	21	21	21	21
**21201**					38	39	39	39	21	21	21	21	47	48	48	48
**21210**	21	21	21	21	12	12	12	12	11	11	11	11	3	3	3	3
**21211**					9	9	9	9	9	9	9	9	11	11	11	11
**21300**	200	201	195	202	149	149	143	150	76	76	74	76	62	62	62	63
**21301**					51	52	52	52	86	86	83	86	82	82	81	82
**21310**	19	19	19	19	11	11	11	11	8	8	8	8	5	5	5	5
**21311**					8	8	8	8	9	9	9	9	9	9	9	9
**21400**	57	56	57	57	42	41	42	42	44	43	44	44	10	10	10	10
**21401**					15	15	15	15	4	4	4	4	22	22	22	22
**21410**	2	2	2	2	2	2	2	2	2	2	2	2	0	0	0	0
**21411**					0	0	0	0	0	0	0	0	1	1	1	1
**21500**	57	57	57	57	37	37	37	37	30	30	30	30	17	17	17	17
**21501**					20	20	20	20	15	15	15	15	22	22	22	22
**21510**	7	7	7	7	5	5	5	5	5	5	5	5	2	2	2	2
**21511**					2	2	2	2	1	1	1	1	2	2	2	2

Note. GAD-2: 2-item Generalised Anxiety Disorder questionnaire; ONS LS: Office for National Statistics' question on life satisfaction; PHQ-2: 2-item depression Patient Health Questionnaire; UCLA-3: 3-item University of California Los Angeles loneliness scale. Stratification 40 is defined by cohort * birth sex * racial/ethnic group * sexual orientation; strata 80a include stratification 40 * within-country index of multiple deprivation rank of the residential area; strata 80b include stratification 40 * housing tenure; strata 80c include stratification 40 * parental social class during childhood (age 11/14).

Intersectional strata are defined by generation/cohort (first digit: 1 Next Steps / 1990, 2 Millennium Cohort Study / 2000-2002), birth sex (second digit: 0 Male, 1 Female), racial/ethnic group (third digit: 1 White, 2 Mixed, 3 South Asian, 4 Black, 5 Other), sexual orientation (fourth digit: 0 Heterosexual, 1 Sexual minority). The fifth digit corresponds to within-country Index of Multiple Deprivation rank in the stratification 80a (0 Below the median/more deprived area, 1 Above the median/less deprived area); housing tenure in stratification 80b (0 Not homeowner, 1 Homeowner / part owner); and parental social class during childhood in stratification 80c (0 Disadvantaged, 1 Advantaged). This fifth digit does not apply to stratification 40, and therefore cells of strata differing only in that last digit are merged under that stratification.

Table S4. Fixed- and random-effects from the main models. Markov Chain Monte Carlo (MCMC) estimation, unweighted results.

	Anxiety symptomatology (GAD-2)		Depressive symptomatology (PHQ-2)		Loneliness (UCLA-3)		Life satisfaction (ONS question)	
**Stratification 40**	**Coefficient (95% CI)**	***p***	**Coefficient (95% CI)**	***p***	**Coefficient (95% CI)**	***p***	**Coefficient (95% CI)**	***p***
**Fixed effects**								
Intercept	0.69 (0.43, 0.91)	<0.001	0.64 (0.37, 0.87)	<0.001	4.26 (4.04, 4.51)	<0.001	7.30 (7.06, 7.51)	<0.001
Cohort/generation (ref. Next Steps / 1990)								
Millennium Cohort Study / 2000-2002	0.46 (0.32, 0.60)	<0.001	0.52 (0.38, 0.68)	<0.001	0.37 (0.23, 0.49)	<0.001	-0.55 (-0.67, -0.40)	<0.001
Birth sex (ref. Male)								
Female	0.77 (0.60, 0.90)	<0.001	0.44 (0.29, 0.57)	<0.001	0.28 (0.14, 0.40)	<0.001	-0.22 (-0.34, -0.09)	0.003
Racial/ethnic group (ref. White)								
Mixed	-0.08 (-0.29, 0.16)	0.247	0.06 (-0.17, 0.29)	0.294	0.10 (-0.11, 0.32)	0.179	-0.20 (-0.43, 0.06)	0.052
South Asian	-0.21 (-0.39, -0.05)	0.008	-0.13 (-0.33, 0.04)	0.064	-0.06 (-0.23, 0.09)	0.223	-0.06 (-0.25, 0.11)	0.210
Black	-0.43 (-0.70, -0.16)	<0.001	-0.17 (-0.43, 0.10)	0.096	-0.11 (-0.36, 0.15)	0.194	-0.05 (-0.33, 0.23)	0.357
Other	-0.21 (-0.49, 0.06)	0.066	0.06 (-0.22, 0.33)	0.340	0.13 (-0.15, 0.41)	0.172	-0.43 (-0.75, -0.12)	0.005
Sexual orientation (ref. Heterosexual)								
Sexual minority	0.71 (0.53, 0.89)	<0.001	0.71 (0.52, 0.88)	<0.001	0.80 (0.62, 0.96)	<0.001	-0.84 (-1.03, -0.67)	<0.001
**Random effects**								
Between-stratum variance	0.01 (0.00, 0.04)		0.01 (0.00, 0.04)		0.01 (0.00, 0.03)		0.01 (0.00, 0.02)	
Within-stratum variance	3.04 (2.95, 3.14)		2.77 (2.69, 2.86)		3.01 (2.92, 3.11)		4.60 (4.45, 4.74)	
**Stratification 80a**	**Coefficient (95% CI)**	***p***	**Coefficient (95% CI)**	***p***	**Coefficient (95% CI)**	***p***	**Coefficient (95% CI)**	***p***
**Fixed effects**								
Intercept	0.75 (0.54, 0.95)	<0.001	0.74 (0.51, 0.95)	<0.001	4.31 (4.11, 4.52)	<0.001	7.21 (6.96, 7.45)	<0.001
Cohort/generation (ref. Next Steps / 1990)								
Millennium Cohort Study / 2000-2002	0.44 (0.33, 0.56)	<0.001	0.50 (0.38, 0.63)	<0.001	0.38 (0.25, 0.49)	<0.001	-0.54 (-0.68, -0.41)	<0.001
Birth sex (ref. Male)								
Female	0.77 (0.65, 0.88)	<0.001	0.44 (0.32, 0.55)	<0.001	0.27 (0.16, 0.39)	<0.001	-0.21 (-0.32, -0.08)	0.004

Racial/ethnic group (ref. White)								
Mixed	-0.09 (-0.30, 0.13)	0.210	0.05 (-0.16, 0.26)	0.319	0.10 (-0.11, 0.29)	0.185	-0.18 (-0.43, 0.06)	0.065
South Asian	-0.22 (-0.36, -0.06)	0.003	-0.14 (-0.30, 0.01)	0.033	-0.07 (-0.22, 0.09)	0.163	-0.03 (-0.20, 0.14)	0.353
Black	-0.47 (-0.71, -0.22)	0.001	-0.21 (-0.45, 0.05)	0.043	-0.13 (-0.38, 0.10)	0.128	-0.01 (-0.32, 0.29)	0.492
Other	-0.23 (-0.49, 0.04)	0.048	0.04 (-0.22, 0.30)	0.364	0.12 (-0.16, 0.41)	0.199	-0.41 (-0.71, -0.10)	0.002
Sexual orientation (ref. Heterosexual)								
Sexual minority	0.71 (0.55, 0.87)	<0.001	0.72 (0.56, 0.88)	<0.001	0.80 (0.63, 0.96)	<0.001	-0.84 (-1.03, -0.66)	<0.001
Residential IMD rank (ref. More deprived)								
Less deprived	-0.06 (-0.18, 0.07)	0.155	-0.12 (-0.24, 0.00)	0.031	-0.09 (-0.20, 0.03)	0.071	0.12 (-0.01, 0.24)	0.033
**Random effects**								
Between-stratum variance	0.01 (0.00, 0.03)		0.01 (0.00, 0.04)		0.01 (0.00, 0.03)		0.01 (0.00, 0.03)	
Within-stratum variance	3.04 (2.95, 3.13)		2.77 (2.68, 2.86)		3.00 (2.91, 3.09)		4.60 (4.45, 4.75)	
**Stratification 80b**	**Coefficient (95% CI)**	***p***	**Coefficient (95% CI)**	***p***	**Coefficient (95% CI)**	***p***	**Coefficient (95% CI)**	***p***
**Fixed effects**								
Intercept	0.84 (0.59, 1.05)	<0.001	0.81 (0.54, 1.03)	<0.001	4.39 (4.16, 4.65)	<0.001	7.18 (6.87, 7.45)	<0.001
Cohort/generation (ref. Next Steps / 1990)								
Millennium Cohort Study / 2000-2002	0.44 (0.32, 0.57)	<0.001	0.48 (0.36, 0.63)	<0.001	0.38 (0.25, 0.50)	<0.001	-0.56 (-0.70, -0.40)	<0.001
Birth sex (ref. Male)								
Female	0.75 (0.62, 0.87)	<0.001	0.43 (0.29, 0.55)	<0.001	0.28 (0.14, 0.42)	<0.001	-0.20 (-0.36, -0.05)	0.006
Racial/ethnic group (ref. White)								
Mixed	-0.05 (-0.28, 0.18)	0.347	0.12 (-0.11, 0.35)	0.124	0.10 (-0.13, 0.33)	0.209	-0.21 (-0.49, 0.07)	0.070
South Asian	-0.17 (-0.32, -0.01)	0.021	-0.09 (-0.25, 0.07)	0.135	-0.03 (-0.21, 0.14)	0.347	-0.14 (-0.33, 0.05)	0.073
Black	-0.48 (-0.74, -0.23)	<0.001	-0.25 (-0.51, -0.01)	0.023	-0.15 (-0.41, 0.11)	0.138	-0.06 (-0.38, 0.25)	0.346
Other	-0.23 (-0.51, 0.04)	0.056	0.02 (-0.25, 0.28)	0.447	0.20 (-0.09, 0.50)	0.095	-0.54 (-0.88, -0.21)	0.001
Sexual orientation (ref. Heterosexual)								
Sexual minority	0.68 (0.54, 0.83)	<0.001	0.71 (0.57, 0.87)	<0.001	0.77 (0.59, 0.94)	<0.001	-0.81 (-0.99, -0.62)	<0.001
Housing tenure (ref. Not homeowner)								

Homeowner / part owner	-0.22 (-0.33, -0.10)	0.001	-0.23 (-0.35, -0.10)	0.001	-0.24 (-0.38, -0.10)	0.001	0.25 (0.10, 0.39)	0.001
**Random effects**								
Between-stratum variance	0.01 (0.00, 0.03)		0.01 (0.00, 0.04)		0.02 (0.01, 0.04)		0.02 (0.00, 0.05)	
Within-stratum variance	2.99 (2.89, 3.08)		2.71 (2.62, 2.80)		2.96 (2.86, 3.06)		4.51 (4.36, 4.65)	
**Stratification 80c**	**Coefficient (95% CI)**	***p***	**Coefficient (95% CI)**	***p***	**Coefficient (95% CI)**	***p***	**Coefficient (95% CI)**	***p***
**Fixed effects**								
Intercept	0.75 (0.50, 0.97)	<0.001	0.80 (0.54, 1.03)	<0.001	4.36 (4.16, 4.58)	<0.001	7.26 (7.02, 7.47)	<0.001
Cohort/generation (ref. Next Steps / 1990)								
Millennium Cohort Study / 2000-2002	0.45 (0.33, 0.60)	<0.001	0.48 (0.35, 0.64)	<0.001	0.40 (0.27, 0.51)	<0.001	-0.58 (-0.70, -0.44)	<0.001
Birth sex (ref. Male)								
Female	0.77 (0.63, 0.90)	<0.001	0.43 (0.29, 0.58)	<0.001	0.26 (0.13, 0.39)	0.001	-0.21 (-0.34, -0.09)	<0.001
Racial/ethnic group (ref. White)								
Mixed	-0.12 (-0.33, 0.12)	0.169	-0.02 (-0.24, 0.22)	0.488	0.05 (-0.17, 0.26)	0.331	-0.17 (-0.41, 0.10)	0.120
South Asian	-0.23 (-0.40, -0.06)	0.004	-0.18 (-0.36, 0.00)	0.024	-0.14 (-0.30, 0.02)	0.056	0.03 (-0.15, 0.22)	0.384
Black	-0.45 (-0.74, -0.18)	0.001	-0.31 (-0.60, -0.04)	0.013	-0.20 (-0.46, 0.08)	0.062	0.01 (-0.32, 0.32)	0.500
Other	-0.23 (-0.52, 0.06)	0.074	0.04 (-0.24, 0.33)	0.380	0.17 (-0.13, 0.47)	0.110	-0.60 (-0.94, -0.25)	<0.001
Sexual orientation (ref. Heterosexual)								
Sexual minority	0.68 (0.52, 0.85)	<0.001	0.69 (0.53, 0.86)	<0.001	0.73 (0.55, 0.91)	<0.001	-0.83 (-1.01, -0.65)	<0.001
Childhood social class (ref. Disadvantaged)								
Advantaged	-0.06 (-0.18, 0.05)	0.172	-0.14 (-0.26, -0.01)	0.023	-0.17 (-0.29, -0.03)	0.009	0.13 (0.01, 0.25)	0.022
**Random effects**								
Between-stratum variance	0.01 (0.00, 0.04)		0.02 (0.00, 0.05)		0.01 (0.00, 0.03)		0.01 (0.00, 0.03)	
Within-stratum variance	3.00 (2.90, 3.09)		2.72 (2.62, 2.80)		2.95 (2.85, 3.05)		4.48 (4.33, 4.62)	

Note. CI: credible interval; GAD-2: 2-item Generalised Anxiety Disorder questionnaire; ONS: Office for National Statistics; *p*: one-sided Bayesian p-value (proportion of the Monte Carlo Markov Chain that is of the opposite sign to the point estimate; PHQ-2: 2-item depression Patient Health Questionnaire; UCLA-3: 3-item University of California Los Angeles loneliness scale.
Stratification 40 is defined by cohort * birth sex * racial/ethnic group * sexual orientation; strata 80a include stratification 40 * within-country index of multiple deprivation rank of the residential area; strata 80b include stratification 40 * housing tenure; strata 80c include stratification 40 * parental social class during childhood (age 11/14).
White includes all White groups; South Asian includes Bangladeshi, Indian, and Pakistani groups; Black includes Black African, Black Caribbean, and Black British groups; Other includes all other ethnic group not included in the other categories.

Table S5. Fixed- and random-effects from the main models. Maximum likelihood estimation, weighted (survey design and non-response) results.

	Anxiety symptomatology (GAD-2)		Depressive symptomatology (PHQ-2)		Loneliness (UCLA-3)		Life satisfaction (ONS question)	
**Stratification 40**	**Coefficient (95% CI)**	***p***	**Coefficient (95% CI)**	***p***	**Coefficient (95% CI)**	***p***	**Coefficient (95% CI)**	***p***
**Fixed effects**								
Intercept	0.52 (0.20, 0.84)	0.001	0.59 (0.19, 0.98)	0.004	4.27 (3.99, 4.55)	<0.001	7.38 (7.21, 7.54)	<0.001
Cohort/generation (ref. Next Steps / 1990)								
Millennium Cohort Study / 2000-2002	0.50 (0.32, 0.68)	<0.001	0.54 (0.32, 0.76)	<0.001	0.36 (0.18, 0.53)	<0.001	-0.63 (-0.72, -0.54)	<0.001
Birth sex (ref. Male)								
Female	0.81 (0.64, 0.99)	<0.001	0.43 (0.22, 0.64)	<0.001	0.28 (0.13, 0.43)	<0.001	-0.14 (-0.23, -0.06)	0.001
Racial/ethnic group (ref. White)								
Mixed	0.21 (-0.01, 0.42)	0.062	0.44 (0.08, 0.80)	0.016	0.38 (0.21, 0.55)	<0.001	-0.60 (-0.80, -0.41)	<0.001
South Asian	-0.05 (-0.30, 0.21)	0.727	-0.03 (-0.35, 0.30)	0.868	0.12 (-0.15, 0.39)	0.381	-0.07 (-0.31, 0.18)	0.584
Black	-0.19 (-0.44, 0.06)	0.141	0.01 (-0.20, 0.21)	0.942	0.08 (-0.22, 0.37)	0.614	-0.54 (-0.96, -0.13)	0.010
Other	0.59 (0.14, 1.05)	0.011	0.37 (-0.18, 0.91)	0.184	0.52 (0.07, 0.97)	0.024	-0.78 (-1.25, -0.30)	0.001
Sexual orientation (ref. Heterosexual)								
Sexual minority	0.56 (0.28, 0.84)	<0.001	0.64 (0.34, 0.93)	<0.001	0.74 (0.58, 0.90)	<0.001	-0.88 (-1.08, -0.69)	<0.001
**Random effects**								
Between-stratum variance	0.02 (0.00, 0.17)		0.05 (0.01, 0.32)		0.01 (0.00, 8.52)		0.00 (0.00, 0.00)	
Within-stratum variance	2.97 (2.66, 3.33)		2.75 (2.57, 2.94)		3.18 (2.97, 3.41)		4.94 (4.67, 5.22)	
**Stratification 80a**	**Coefficient (95% CI)**	***p***	**Coefficient (95% CI)**	***p***	**Coefficient (95% CI)**	***p***	**Coefficient (95% CI)**	***p***
**Fixed effects**								
Intercept	0.49 (0.11, 0.88)	0.011	0.63 (0.12, 1.14)	0.016	4.32 (4.02, 4.62)	<0.001	7.21 (6.77, 7.65)	<0.001
Cohort/generation (ref. Next Steps / 1990)								
Millennium Cohort Study / 2000-2002	0.51 (0.29, 0.73)	<0.001	0.54 (0.28, 0.80)	<0.001	0.36 (0.16, 0.55)	<0.001	-0.56 (-0.81, -0.30)	<0.001
Birth sex (ref. Male)								
Female	0.78 (0.57, 1.00)	<0.001	0.43 (0.17, 0.68)	0.001	0.28 (0.10, 0.46)	0.002	0.00 (-0.26, 0.26)	0.994

Racial/ethnic group (ref. White)								
Mixed	0.21 (-0.03, 0.46)	0.084	0.40 (0.04, 0.76)	0.028	0.36 (0.10, 0.62)	0.007	-0.62 (-0.95, -0.29)	<0.001
South Asian	0.01 (-0.27, 0.29)	0.943	-0.02 (-0.36, 0.32)	0.903	0.12 (-0.16, 0.39)	0.407	-0.22 (-0.66, 0.22)	0.323
Black	-0.18 (-0.42, 0.07)	0.161	0.06 (-0.33, 0.45)	0.768	0.04 (-0.30, 0.37)	0.829	-0.46 (-0.95, 0.04)	0.071
Other	0.55 (-0.06, 1.16)	0.078	0.25 (-0.36, 0.86)	0.423	0.48 (0.05, 0.91)	0.027	-0.75 (-1.34, -0.15)	0.014
Sexual orientation (ref. Heterosexual)								
Sexual minority	0.60 (0.30, 0.89)	<0.001	0.63 (0.31, 0.95)	<0.001	0.74 (0.55, 0.93)	<0.001	-0.94 (-1.29, -0.58)	<0.001
Residential IMD rank (ref. More deprived)								
Less deprived	0.04 (-0.18, 0.25)	0.746	-0.06 (-0.33, 0.20)	0.629	-0.12 (-0.30, 0.06)	0.177	0.01 (-0.25, 0.27)	0.935
**Random effects**								
Between-stratum variance	0.09 (0.03, 0.30)		0.17 (0.06, 0.51)		0.05 (0.01, 0.23)		0.12 (0.02, 0.62)	
Within-stratum variance	2.96 (2.71, 3.23)		2.71 (2.54, 2.89)		3.16 (2.96, 3.37)		4.89 (4.54, 5.26)	
**Stratification 80b**	**Coefficient (95% CI)**	***p***	**Coefficient (95% CI)**	***p***	**Coefficient (95% CI)**	***p***	**Coefficient (95% CI)**	***p***
**Fixed effects**								
Intercept	0.62 (0.28, 0.96)	<0.001	0.67 (0.13, 1.22)	0.015	4.39 (3.84, 4.94)	<0.001	7.09 (6.47, 7.70)	<0.001
Cohort/generation (ref. Next Steps / 1990)								
Millennium Cohort Study / 2000-2002	0.50 (0.32, 0.68)	<0.001	0.51 (0.26, 0.77)	<0.001	0.36 (0.08, 0.64)	0.010	-0.63 (-0.96, -0.29)	<0.001
Birth sex (ref. Male)								
Female	0.78 (0.60, 0.96)	<0.001	0.41 (0.16, 0.65)	0.001	0.24 (-0.03, 0.51)	0.081	0.09 (-0.24, 0.42)	0.591
Racial/ethnic group (ref. White)								
Mixed	0.26 (0.06, 0.46)	0.010	0.53 (0.15, 0.92)	0.007	0.41 (0.05, 0.78)	0.026	-0.68 (-1.08, -0.29)	0.001
South Asian	0.00 (-0.24, 0.24)	0.989	0.00 (-0.35, 0.36)	0.986	0.18 (-0.16, 0.53)	0.292	-0.38 (-0.87, 0.12)	0.137
Black	-0.12 (-0.45, 0.21)	0.470	0.05 (-0.27, 0.37)	0.773	0.09 (-0.28, 0.45)	0.633	-0.60 (-1.15, -0.05)	0.033
Other	0.63 (0.11, 1.16)	0.018	0.41 (-0.07, 0.88)	0.092	0.53 (-0.08, 1.13)	0.090	-0.84 (-1.49, -0.19)	0.011
Sexual orientation (ref. Heterosexual)								
Sexual minority	0.54 (0.29, 0.79)	<0.001	0.63 (0.32, 0.95)	<0.001	0.69 (0.39, 0.99)	<0.001	-0.85 (-1.27, -0.43)	<0.001
Housing tenure (ref. Not homeowner)								

Homeowner / part owner	-0.18 (-0.37, 0.00)	0.048	-0.11 (-0.36, 0.14)	0.391	-0.19 (-0.47, 0.09)	0.183	0.39 (0.07, 0.71)	0.017
**Random effects**								
Between-stratum variance	0.05 (0.01, 0.22)		0.14 (0.06, 0.34)		0.18 (0.08, 0.40)		0.24 (0.11, 0.53)	
Within-stratum variance	2.94 (2.67, 3.23)		2.67 (2.44, 2.93)		3.11 (2.86, 3.38)		4.81 (4.55, 5.09)	
**Stratification 80c**	**Coefficient (95% CI)**	***p***	**Coefficient (95% CI)**	***p***	**Coefficient (95% CI)**	***p***	**Coefficient (95% CI)**	***p***
**Fixed effects**								
Intercept	0.60 (0.37, 0.83)	<0.001	0.80 (0.55, 1.06)	<0.001	4.42 (4.22, 4.62)	<0.001	7.01 (6.34, 7.67)	<0.001
Cohort/generation (ref. Next Steps / 1990)								
Millennium Cohort Study / 2000-2002	0.46 (0.32, 0.60)	<0.001	0.41 (0.27, 0.56)	<0.001	0.39 (0.26, 0.52)	<0.001	-0.39 (-0.74, -0.04)	0.028
Birth sex (ref. Male)								
Female	0.81 (0.68, 0.94)	<0.001	0.40 (0.25, 0.54)	<0.001	0.21 (0.07, 0.34)	0.002	-0.07 (-0.44, 0.30)	0.717
Racial/ethnic group (ref. White)								
Mixed	0.09 (-0.11, 0.28)	0.380	0.28 (-0.15, 0.71)	0.203	0.29 (0.18, 0.40)	<0.001	-0.52 (-0.96, -0.08)	0.019
South Asian	-0.16 (-0.37, 0.04)	0.110	-0.19 (-0.34, -0.04)	0.013	-0.12 (-0.25, 0.01)	0.063	-0.16 (-0.78, 0.45)	0.601
Black	-0.04 (-0.22, 0.15)	0.704	0.00 (-0.21, 0.20)	0.984	0.08 (-0.24, 0.40)	0.639	-0.35 (-1.06, 0.36)	0.330
Other	0.40 (0.00, 0.79)	0.047	0.31 (-0.09, 0.71)	0.126	0.21 (-0.24, 0.66)	0.361	-0.59 (-1.32, 0.14)	0.112
Sexual orientation (ref. Heterosexual)								
Sexual minority	0.46 (0.21, 0.71)	<0.001	0.58 (0.41, 0.75)	<0.001	0.63 (0.36, 0.91)	<0.001	-0.84 (-1.43, -0.25)	0.005
Childhood social class (ref. Disadvantaged)								
Advantaged	-0.01 (-0.16, 0.14)	0.906	-0.03 (-0.16, 0.11)	0.690	-0.22 (-0.34, -0.10)	<0.001	0.07 (-0.32, 0.46)	0.715
**Random effects**								
Between-stratum variance	0.01 (0.00, 0.10)		0.02 (0.00, 0.12)		0.01 (0.00, 0.20)		0.35 (0.10, 1.17)	
Within-stratum variance	2.88 (2.62, 3.16)		2.67 (2.54, 2.81)		3.05 (2.88, 3.22)		4.66 (4.36, 4.98)	

Note. CI: confidence interval; GAD-2: 2-item Generalised Anxiety Disorder questionnaire; ONS: Office for National Statistics; PHQ-2: 2-item depression Patient Health Questionnaire; UCLA-3: 3-item University of California Los Angeles loneliness scale. Stratification 40 is defined by cohort * birth sex * racial/ethnic group * sexual orientation; strata 80a include stratification 40 * within-country index of multiple deprivation rank of the residential area; strata 80b include stratification 40 * housing tenure; strata 80c include stratification 40 * parental social class during childhood (age 11/14). White includes all White groups; South Asian includes Bangladeshi, Indian, and Pakistani groups; Black includes Black African, Black Caribbean, and Black British groups; Other includes all other ethnic group not included in the other categories.

Table S6. Results from the fixed-effects multiple regression approach using stratification 40 (cohort * birth sex * ethnic group * sexual orientation).

	GAD-2				PHQ-2			
	Unweighted		Weighted		Unweighted		Weighted	
	Coefficient (95% CI)	*p*	Coefficient (95% CI)	*p*	Coefficient (95% CI)	*p*	Coefficient (95% CI)	*p*
**Cohort (MCS)**	0.27 (0.13, 0.42)	<0.001	0.26 (0.00, 0.51)	0.046	0.29 (0.15, 0.43)	<0.001	0.28 (0.04, 0.51)	0.023
**Birth sex (female)**	0.70 (0.56, 0.83)	<0.001	0.74 (0.50, 0.98)	<0.001	0.30 (0.17, 0.43)	<0.001	0.40 (0.20, 0.61)	<0.001
**Ethnic group**								
Mixed	-0.05 (-0.55, 0.44)	0.835	-0.27 (-0.85, 0.30)	0.351	0.02 (-0.45, 0.48)	0.948	0.00 (-0.55, 0.54)	0.989
South Asian	-0.03 (-0.29, 0.22)	0.791	-0.01 (-0.42, 0.41)	0.972	-0.12 (-0.36, 0.13)	0.342	-0.28 (-0.60, 0.03)	0.079
Black	-0.65 (-1.16, -0.13)	0.014	-0.26 (-0.99, 0.48)	0.492	-0.32 (-0.81, 0.17)	0.199	-0.01 (-0.61, 0.59)	0.977
Other	-0.30 (-1.00, 0.41)	0.411	0.76 (-0.55, 2.07)	0.254	0.01 (-0.66, 0.69)	0.969	1.11 (-0.22, 2.43)	0.102
**Sexual orientation (sexual minority)**	0.56 (0.20, 0.93)	0.003	-0.04 (-0.59, 0.51)	0.879	0.48 (0.13, 0.83)	0.008	0.43 (-0.19, 1.06)	0.172
**Cohort * birth sex**								
MCS * female	0.26 (0.07, 0.45)	0.007	0.23 (-0.09, 0.56)	0.159	0.22 (0.05, 0.40)	0.014	0.12 (-0.20, 0.44)	0.468
**Cohort * ethnic group**								
MCS * Mixed	-0.04 (-0.71, 0.63)	0.904	0.48 (-0.30, 1.27)	0.228	0.12 (-0.51, 0.75)	0.711	1.00 (-0.04, 2.04)	0.060
MCS * South Asian	-0.31 (-0.70, 0.09)	0.128	-0.36 (-0.88, 0.16)	0.179	-0.12 (-0.49, 0.26)	0.540	0.15 (-0.43, 0.73)	0.615
MCS * Black	0.46 (-0.28, 1.20)	0.220	-0.31 (-1.19, 0.57)	0.494	0.45 (-0.26, 1.16)	0.211	0.13 (-0.88, 1.15)	0.799
MCS * Other	0.23 (-0.71, 1.17)	0.634	0.16 (-1.64, 1.97)	0.861	-0.10 (-1.00, 0.80)	0.830	-1.24 (-2.67, 0.19)	0.090
**Birth sex * ethnic group**								
Female * Mixed	-0.04 (-0.64, 0.55)	0.894	0.43 (-0.58, 1.44)	0.404	0.09 (-0.48, 0.65)	0.757	0.28 (-0.72, 1.28)	0.584
Female * South Asian	-0.21 (-0.54, 0.11)	0.200	-0.20 (-0.72, 0.31)	0.432	0.00 (-0.31, 0.31)	0.983	0.01 (-0.38, 0.41)	0.957
Female * Black	-0.04 (-0.66, 0.59)	0.910	0.18 (-0.86, 1.21)	0.734	-0.06 (-0.66, 0.53)	0.840	-0.04 (-0.98, 0.90)	0.936
Female * Other	-0.09 (-0.92, 0.74)	0.834	-0.74 (-2.20, 0.71)	0.317	-0.12 (-0.91, 0.68)	0.774	-1.04 (-2.44, 0.36)	0.145
**Cohort * birth sex * ethnic group**								
MCS * Female * Mixed	0.02 (-0.81, 0.86)	0.955	-0.49 (-1.78, 0.81)	0.461	-0.22 (-1.01, 0.57)	0.584	-0.95 (-2.44, 0.55)	0.213
MCS * Female * South Asian	0.40 (-0.12, 0.91)	0.130	0.76 (-0.17, 1.69)	0.110	0.37 (-0.12, 0.86)	0.140	0.80 (-0.73, 2.32)	0.304
MCS * Female * Black	0.04 (-0.90, 0.98)	0.933	0.19 (-1.22, 1.61)	0.787	0.00 (-0.90, 0.90)	0.997	-0.32 (-1.84, 1.21)	0.684
MCS * Female * Other	-0.16 (-1.30, 0.97)	0.777	0.15 (-1.97, 2.28)	0.888	0.65 (-0.43, 1.73)	0.240	1.79 (0.10, 3.48)	0.038
**Cohort * sexual orientation**								
MCS * sexual minority	0.33 (-0.16, 0.82)	0.190	0.93 (0.12, 1.73)	0.025	0.51 (0.04, 0.98)	0.034	0.40 (-0.36, 1.17)	0.304
**Birth sex * sexual orientation**								
Female * sexual minority	0.10 (-0.39, 0.59)	0.680	0.33 (-0.63, 1.29)	0.504	0.33 (-0.14, 0.80)	0.164	0.20 (-0.66, 1.05)	0.652
**Cohort * birth sex * sexual orientation**								
MCS * Female * sexual minority	-0.36 (-0.98, 0.26)	0.258	-0.72 (-1.95, 0.50)	0.248	-0.57 (-1.17, 0.02)	0.060	-0.41 (-1.48, 0.66)	0.450
**Ethnic group * sexual orientation**								
Mixed * sexual minority	-0.22 (-2.72, 2.27)	0.860	0.34 (-0.54, 1.21)	0.451	0.23 (-2.15, 2.61)	0.850	-0.31 (-1.47, 0.85)	0.603
South Asian * sexual minority	-0.10 (-1.11, 0.91)	0.847	0.84 (-0.14, 1.82)	0.092	-0.21 (-1.17, 0.76)	0.672	0.43 (-0.41, 1.26)	0.314
Black * sexual minority	-0.13 (-2.62, 2.37)	0.919	0.13 (-0.76, 1.03)	0.771	2.07 (-0.32, 4.45)	0.089	1.58 (0.59, 2.58)	0.002
Other * sexual minority	2.19 (0.58, 3.79)	0.008	3.04 (1.37, 4.70)	<0.001	1.40 (-0.13, 2.93)	0.073	0.96 (-1.56, 3.48)	0.456
**Cohort * ethnic group * sexual orientation**								
MCS * Mixed * sexual minority	0.77 (-2.14, 3.68)	0.603	0.52 (-1.19, 2.24)	0.549	-0.59 (-3.36, 2.19)	0.679	-1.10 (-2.90, 0.70)	0.232
MCS * South Asian * sexual minority	0.06 (-1.82, 1.94)	0.950	-0.24 (-1.61, 1.13)	0.732	-0.51 (-2.31, 1.29)	0.579	0.46 (-2.00, 2.92)	0.715

MCS * Black * sexual minority	-0.07 (-3.59, 3.46)	0.971	0.12 (-1.03, 1.28)	0.833	-3.25 (-6.62, 0.12)	0.059	-3.22 (-5.51, -0.92)	0.006
MCS * Other * sexual minority	-3.00 (-5.98, -0.02)	0.049	-6.13 (-8.27, -4.00)	<0.001	-2.87 (-5.71, -0.02)	0.048	-3.10 (-5.71, -0.49)	0.020
**Birth sex * ethnic group * sexual orientation**								
Female * Mixed * sexual minority	-0.26 (-3.04, 2.52)	0.855	-0.71 (-2.22, 0.80)	0.359	-1.28 (-3.93, 1.37)	0.344	-1.31 (-3.15, 0.52)	0.161
Female * South Asian * sexual minority	-0.06 (-1.83, 1.70)	0.944	-2.13 (-3.72, -0.53)	0.009	-0.77 (-2.38, 0.83)	0.345	-1.71 (-2.87, -0.56)	0.004
Female * Black * sexual minority	1.24 (-1.73, 4.20)	0.414	0.26 (-1.57, 2.08)	0.781	-1.67 (-4.50, 1.16)	0.247	-1.68 (-3.35, 0.00)	0.049
Female * Other * sexual minority	-2.88 (-5.28, -0.47)	0.019	-4.05 (-6.46, -1.63)	0.001	-1.93 (-4.23, 0.36)	0.099	-2.14 (-5.08, 0.80)	0.154
**Cohort * birth sex * ethnic group * sexual orientation**								
MCS * Female * Mixed * sexual minority	-0.02 (-3.29, 3.25)	0.991	0.82 (-1.80, 3.45)	0.539	2.31 (-0.81, 5.43)	0.146	4.06 (1.40, 6.72)	0.003
MCS * Female * South Asian * sexual minority	-0.61 (-3.13, 1.91)	0.636	1.41 (-1.13, 3.95)	0.277	0.99 (-1.36, 3.35)	0.407	0.64 (-2.53, 3.81)	0.693
MCS * Female * Black * sexual minority	-0.44 (-5.03, 4.15)	0.851	0.45 (-1.73, 2.64)	0.683	2.45 (-1.94, 6.83)	0.274	3.47 (0.46, 6.48)	0.024
MCS * Female * Other * sexual minority	4.36 (0.62, 8.10)	0.022	7.25 (4.13, 10.38)	<0.001	2.54 (-1.03, 6.12)	0.164	2.92 (-0.35, 6.19)	0.080
**Intercept**	1.21 (1.11, 1.32)	<0.001	1.17 (0.97, 1.36)	<0.001	1.28 (1.18, 1.38)	<0.001	1.25 (1.08, 1.41)	<0.001

	**UCLA-3**				**ONS life satisfaction**			
	**Unweighted**		**Weighted**		**Unweighted**		**Weighted**	
	**Coefficient (95% CI)**	***p***	**Coefficient (95% CI)**	***p***	**Coefficient (95% CI)**	***p***	**Coefficient (95% CI)**	***p***
**Cohort (MCS)**	0.55 (0.41, 0.70)	<0.001	0.48 (0.22, 0.74)	<0.001	-0.61 (-0.78, -0.43)	<0.001	-0.64 (-0.97, -0.30)	<0.001
**Birth sex (female)**	0.33 (0.20, 0.47)	<0.001	0.36 (0.13, 0.59)	0.002	-0.22 (-0.38, -0.05)	0.011	-0.21 (-0.50, 0.09)	0.165
**Ethnic group**								
Mixed	0.40 (-0.09, 0.89)	0.108	0.38 (-0.15, 0.91)	0.156	-0.57 (-1.17, 0.03)	0.064	-0.76 (-1.77, 0.25)	0.141
South Asian	0.12 (-0.13, 0.37)	0.360	0.14 (-0.45, 0.73)	0.641	-0.05 (-0.36, 0.26)	0.764	0.13 (-0.38, 0.64)	0.621
Black	0.14 (-0.38, 0.66)	0.598	0.46 (-0.46, 1.38)	0.325	0.17 (-0.46, 0.80)	0.599	-0.97 (-2.63, 0.70)	0.254
Other	0.50 (-0.20, 1.20)	0.162	1.04 (-0.61, 2.70)	0.216	-1.04 (-1.90, -0.17)	0.019	-1.53 (-2.79, -0.28)	0.017
**Sexual orientation (sexual minority)**	0.93 (0.56, 1.29)	<0.001	0.49 (-0.21, 1.19)	0.170	-1.15 (-1.60, -0.70)	<0.001	-0.78 (-1.88, 0.31)	0.161
**Cohort * birth sex**								
MCS * female	-0.15 (-0.34, 0.04)	0.120	-0.22 (-0.56, 0.11)	0.193	-0.01 (-0.24, 0.22)	0.927	0.03 (-0.41, 0.46)	0.900
**Cohort * ethnic group**								
MCS * Mixed	-0.65 (-1.31, 0.00)	0.052	-0.04 (-0.98, 0.90)	0.933	0.58 (-0.23, 1.40)	0.161	0.28 (-1.12, 1.69)	0.691
MCS * South Asian	-0.58 (-0.97, -0.19)	0.004	-0.51 (-1.21, 0.20)	0.157	0.15 (-0.33, 0.63)	0.536	-0.17 (-0.94, 0.60)	0.667
MCS * Black	-0.61 (-1.35, 0.14)	0.110	-0.98 (-2.21, 0.26)	0.120	-0.42 (-1.33, 0.49)	0.364	0.22 (-1.80, 2.24)	0.833
MCS * Other	-0.42 (-1.37, 0.53)	0.385	0.07 (-1.91, 2.05)	0.944	1.05 (-0.11, 2.21)	0.076	0.66 (-1.53, 2.85)	0.553
**Birth sex * ethnic group**								
Female * Mixed	-0.26 (-0.85, 0.32)	0.380	-0.11 (-1.06, 0.85)	0.828	0.47 (-0.26, 1.20)	0.205	0.37 (-1.01, 1.75)	0.600
Female * South Asian	-0.09 (-0.42, 0.23)	0.567	-0.16 (-0.85, 0.54)	0.661	-0.07 (-0.47, 0.33)	0.722	-0.03 (-0.61, 0.56)	0.928
Female * Black	-0.26 (-0.89, 0.37)	0.420	-0.29 (-1.50, 0.92)	0.640	-0.43 (-1.19, 0.33)	0.269	0.38 (-1.36, 2.11)	0.670
Female * Other	-0.20 (-1.02, 0.63)	0.638	-0.90 (-2.82, 1.01)	0.354	0.78 (-0.24, 1.80)	0.133	1.45 (-0.04, 2.93)	0.056
**Cohort * birth sex * ethnic group**								
MCS * Female * Mixed	0.69 (-0.13, 1.52)	0.099	0.06 (-1.30, 1.41)	0.936	-0.78 (-1.79, 0.24)	0.134	-0.74 (-2.56, 1.08)	0.424
MCS * Female * South Asian	0.57 (0.06, 1.08)	0.028	1.26 (-0.35, 2.87)	0.126	-0.12 (-0.75, 0.50)	0.704	-0.43 (-1.92, 1.06)	0.574
MCS * Female * Black	0.89 (-0.05, 1.83)	0.064	0.74 (-0.93, 2.41)	0.387	0.90 (-0.25, 2.05)	0.125	0.96 (-1.24, 3.16)	0.393
MCS * Female * Other	0.14 (-1.00, 1.28)	0.808	-0.03 (-2.29, 2.23)	0.981	-1.66 (-3.06, -0.27)	0.020	-1.53 (-4.03, 0.98)	0.232
**Cohort * sexual orientation**								
MCS * sexual minority	-0.03 (-0.52, 0.46)	0.910	0.28 (-0.70, 1.25)	0.576	0.38 (-0.22, 0.98)	0.215	-0.21 (-1.44, 1.01)	0.735

**Birth sex * sexual orientation**								
Female * sexual minority	-0.19 (-0.68, 0.30)	0.448	0.29 (-0.67, 1.24)	0.554	0.26 (-0.34, 0.87)	0.393	-0.05 (-1.44, 1.33)	0.939
**Cohort * birth sex * sexual orientation**								
MCS * Female * sexual minority	0.09 (-0.52, 0.71)	0.765	-0.23 (-1.50, 1.03)	0.719	-0.26 (-1.03, 0.50)	0.500	0.31 (-1.23, 1.85)	0.693
**Ethnic group * sexual orientation**								
Mixed * sexual minority	1.13 (-1.35, 3.61)	0.371	2.18 (0.99, 3.37)	<0.001	0.93 (-2.13, 4.00)	0.551	0.77 (-0.69, 2.24)	0.300
South Asian * sexual minority	-0.01 (-1.02, 0.99)	0.977	0.44 (-0.95, 1.83)	0.534	-0.30 (-1.55, 0.94)	0.631	-2.53 (-4.78, -0.28)	0.028
Black * sexual minority	-0.61 (-4.07, 2.85)	0.730	-0.53 (-1.67, 0.61)	0.361	-1.31 (-4.38, 1.76)	0.403	-0.27 (-2.32, 1.78)	0.794
Other * sexual minority	-0.14 (-1.73, 1.46)	0.868	0.08 (-2.98, 3.15)	0.957	0.40 (-1.57, 2.37)	0.692	-0.32 (-3.99, 3.35)	0.865
**Cohort * ethnic group * sexual orientation**								
MCS * Mixed * sexual minority	-1.87 (-4.76, 1.02)	0.205	-3.13 (-4.97, -1.29)	0.001	-0.36 (-3.93, 3.21)	0.844	0.74 (-1.98, 3.47)	0.594
MCS * South Asian * sexual minority	0.08 (-1.79, 1.96)	0.930	-0.07 (-1.77, 1.63)	0.938	-2.01 (-4.33, 0.30)	0.088	1.25 (-1.53, 4.03)	0.377
MCS * Black * sexual minority	1.08 (-3.80, 5.97)	0.664	1.22 (-0.33, 2.77)	0.122	3.14 (-1.19, 7.48)	0.155	4.19 (-0.09, 8.46)	0.055
MCS * Other * sexual minority	-1.44 (-4.41, 1.53)	0.343	-3.89 (-7.19, -0.60)	0.021	1.67 (-1.99, 5.34)	0.371	3.10 (-1.03, 7.23)	0.141
**Birth sex * ethnic group * sexual orientation**								
Female * Mixed * sexual minority	-1.43 (-4.20, 1.33)	0.309	-2.56 (-4.25, -0.86)	0.003	-1.52 (-4.93, 1.90)	0.384	-1.06 (-3.22, 1.10)	0.335
Female * South Asian * sexual minority	0.38 (-1.37, 2.13)	0.670	0.24 (-1.47, 1.94)	0.785	-0.26 (-2.33, 1.81)	0.804	1.67 (-2.13, 5.48)	0.388
Female * Black * sexual minority	0.52 (-3.29, 4.32)	0.790	0.79 (-1.11, 2.68)	0.415	2.28 (-1.36, 5.93)	0.220	1.45 (-1.09, 3.99)	0.264
Female * Other * sexual minority	0.22 (-2.17, 2.61)	0.855	-0.10 (-3.48, 3.28)	0.954	-0.08 (-3.04, 2.88)	0.957	0.77 (-3.32, 4.87)	0.711
**Cohort * birth sex * ethnic group * sexual orientation**								
MCS * Female * Mixed * sexual minority	2.39 (-0.86, 5.64)	0.149	4.51 (1.95, 7.08)	0.001	1.00 (-3.02, 5.02)	0.625	-0.84 (-4.39, 2.72)	0.644
MCS * Female * South Asian * sexual minority	-0.76 (-3.26, 1.75)	0.554	-1.68 (-4.52, 1.15)	0.244	3.22 (0.19, 6.25)	0.037	1.02 (-3.53, 5.58)	0.659
MCS * Female * Black * sexual minority	-3.23 (-8.93, 2.46)	0.266	-3.43 (-5.78, -1.08)	0.004	-5.53 (-11.18, 0.12)	0.055	-7.22 (-11.72, -2.73)	0.002
MCS * Female * Other * sexual minority	0.25 (-3.48, 3.97)	0.897	2.71 (-1.02, 6.45)	0.155	-0.31 (-4.92, 4.29)	0.894	-1.73 (-6.50, 3.05)	0.478
**Intercept**	4.54 (4.43, 4.65)	<0.001	4.58 (4.39, 4.77)	<0.001	6.79 (6.66, 6.92)	<0.001	6.77 (6.52, 7.01)	<0.001

Note. CI: confidence interval; GAD-2: 2-item Generalised Anxiety Disorder questionnaire; MCS: Millennium Cohort Study (born in 2000-2002); NS: Next Steps study (born in 1990); ONS: Office for National Statistics; PHQ-2: 2-item depression Patient Health Questionnaire; UCLA-3: 3-item University of California Los Angeles loneliness scale.

Weighted results are adjusted for survey design characteristics, including survey and non-response weights. White includes all White groups; South Asian includes Bangladeshi, Indian, and Pakistani groups; Black includes Black African, Black Caribbean, and Black British groups; Other includes all other ethnic group not included in the other categories.

Table S7. Results from the fixed-effects multiple regression approach using stratification 80a (cohort * birth sex * ethnic group * sexual orientation * residential Index of Multiple Deprivation rank).

	GAD-2				PHQ-2			
	Unweighted		Weighted		Unweighted		Weighted	
	Coefficient (95% CI)	*p*	Coefficient (95% CI)	*p*	Coefficient (95% CI)	*p*	Coefficient (95% CI)	*p*
**Cohort (MCS)**	0.24 (0.02, 0.47)	0.034	0.20 (-0.16, 0.56)	0.269	0.27 (0.05, 0.48)	0.014	0.22 (-0.16, 0.60)	0.256
**Birth sex (female)**	0.71 (0.51, 0.91)	<0.001	0.77 (0.42, 1.12)	<0.001	0.33 (0.14, 0.53)	0.001	0.45 (0.14, 0.76)	0.005
**Ethnic group**								
Mixed	-0.07 (-0.76, 0.62)	0.844	-0.21 (-1.06, 0.64)	0.634	0.01 (-0.65, 0.67)	0.975	0.13 (-0.68, 0.94)	0.748
South Asian	-0.30 (-0.61, 0.01)	0.062	-0.29 (-0.76, 0.19)	0.235	-0.37 (-0.67, -0.07)	0.016	-0.46 (-0.84, -0.07)	0.020
Black	-0.85 (-1.46, -0.23)	0.007	-0.23 (-1.12, 0.66)	0.610	-0.43 (-1.02, 0.15)	0.147	0.11 (-0.61, 0.82)	0.772
Other	-0.15 (-1.11, 0.81)	0.766	1.26 (-0.34, 2.86)	0.122	0.70 (-0.21, 1.62)	0.132	1.89 (0.33, 3.44)	0.017
**Sexual orientation (sexual minority)**	0.68 (0.19, 1.18)	0.007	-0.02 (-0.80, 0.76)	0.954	0.49 (0.02, 0.97)	0.040	0.65 (-0.08, 1.38)	0.080
**Residential IMD rank (Less deprived)**	-0.18 (-0.39, 0.04)	0.111	-0.11 (-0.53, 0.31)	0.601	-0.19 (-0.39, 0.02)	0.076	-0.07 (-0.42, 0.29)	0.711
**Cohort * birth sex**								
MCS * female	0.24 (-0.05, 0.52)	0.109	0.28 (-0.21, 0.77)	0.262	0.25 (-0.03, 0.52)	0.079	0.32 (-0.18, 0.82)	0.206
**Cohort * ethnic group**								
MCS * Mixed	-0.28 (-1.19, 0.63)	0.547	0.03 (-1.05, 1.11)	0.956	0.04 (-0.82, 0.91)	0.923	0.95 (-0.84, 2.73)	0.298
MCS * South Asian	-0.11 (-0.59, 0.38)	0.668	-0.12 (-0.78, 0.54)	0.720	0.03 (-0.43, 0.49)	0.901	0.13 (-0.53, 0.79)	0.707
MCS * Black	0.48 (-0.38, 1.34)	0.277	-0.48 (-1.51, 0.55)	0.359	0.32 (-0.50, 1.15)	0.443	-0.12 (-1.31, 1.07)	0.847
MCS * Other	0.15 (-1.08, 1.39)	0.808	-0.09 (-2.31, 2.12)	0.934	-0.59 (-1.77, 0.59)	0.324	-1.70 (-3.35, -0.06)	0.043
**Birth sex * ethnic group**								
Female * Mixed	-0.36 (-1.18, 0.45)	0.385	0.34 (-1.04, 1.72)	0.628	-0.29 (-1.07, 0.48)	0.458	0.14 (-1.22, 1.50)	0.840
Female * South Asian	-0.10 (-0.50, 0.31)	0.639	-0.19 (-0.79, 0.42)	0.546	0.11 (-0.27, 0.50)	0.561	0.05 (-0.43, 0.53)	0.839
Female * Black	0.09 (-0.64, 0.83)	0.807	-0.01 (-1.30, 1.28)	0.986	-0.12 (-0.82, 0.58)	0.733	-0.59 (-1.57, 0.39)	0.234
Female * Other	0.04 (-1.08, 1.15)	0.948	-1.02 (-2.78, 0.75)	0.258	-0.51 (-1.57, 0.55)	0.342	-1.68 (-3.32, -0.04)	0.044
**Cohort * birth sex * ethnic group**								
MCS * Female * Mixed	0.69 (-0.43, 1.81)	0.230	-0.01 (-1.65, 1.63)	0.994	0.13 (-0.93, 1.20)	0.805	-0.89 (-3.06, 1.27)	0.417
MCS * Female * South Asian	0.36 (-0.27, 0.98)	0.261	0.84 (-0.36, 2.04)	0.171	0.30 (-0.30, 0.89)	0.328	1.26 (-0.70, 3.23)	0.208
MCS * Female * Black	0.12 (-0.98, 1.22)	0.827	0.39 (-1.35, 2.13)	0.659	0.08 (-0.97, 1.13)	0.875	0.06 (-1.72, 1.83)	0.948
MCS * Female * Other	-0.83 (-2.30, 0.65)	0.272	-1.16 (-3.59, 1.27)	0.351	0.48 (-0.93, 1.89)	0.504	1.15 (-0.62, 2.92)	0.203
**Cohort * sexual orientation**								
MCS * sexual minority	-0.04 (-0.74, 0.65)	0.906	1.19 (0.06, 2.33)	0.039	0.26 (-0.40, 0.93)	0.433	0.25 (-0.86, 1.36)	0.662
**Birth sex * sexual orientation**								
Female * sexual minority	-0.08 (-0.78, 0.61)	0.812	-0.23 (-1.51, 1.04)	0.719	0.44 (-0.22, 1.10)	0.193	-0.43 (-1.40, 0.55)	0.392
**Cohort * birth sex * sexual orientation**								
MCS * Female * sexual minority	0.18 (-0.72, 1.07)	0.700	-0.76 (-2.48, 0.96)	0.386	-0.38 (-1.23, 0.48)	0.386	-0.12 (-1.63, 1.39)	0.879
**Ethnic group * sexual orientation**								
Mixed * sexual minority	-0.41 (-2.97, 2.14)	0.752	0.19 (-1.00, 1.38)	0.750	0.12 (-2.32, 2.56)	0.922	-0.69 (-2.04, 0.66)	0.316
South Asian * sexual minority	0.46 (-0.95, 1.87)	0.521	1.29 (-1.03, 3.61)	0.277	0.64 (-0.70, 1.99)	0.349	0.51 (-1.02, 2.05)	0.512
Black * sexual minority	-2.93 (-7.71, 1.85)	0.229	-0.94 (-3.46, 1.58)	0.465	3.67 (-0.88, 8.23)	0.114	6.25 (3.32, 9.17)	<0.001
Other * sexual minority	2.50 (0.26, 4.74)	0.029	2.80 (0.80, 4.80)	0.006	1.10 (-1.04, 3.23)	0.315	0.05 (-3.49, 3.59)	0.977
**Cohort * ethnic group * sexual orientation**								
MCS * Mixed * sexual minority	2.58 (-0.73, 5.89)	0.127	1.49 (-0.97, 3.95)	0.234	0.76 (-2.40, 3.92)	0.638	0.21 (-2.42, 2.85)	0.873
MCS * South Asian * sexual minority	0.76 (-2.98, 4.50)	0.690	-0.48 (-2.96, 2.01)	0.707	-1.70 (-5.27, 1.86)	0.349	-1.58 (-3.40, 0.25)	0.090
MCS * Black * sexual minority	3.11 (-2.81, 9.04)	0.303	1.05 (-1.63, 3.74)	0.441	-5.96 (-11.61, -0.31)	0.039	-8.63 (-11.81, -5.45)	<0.001
MCS * Other * sexual minority	-1.54 (-7.74, 4.65)	0.626	-4.05 (-7.68, -0.42)	0.029	-1.50 (-7.41, 4.40)	0.618	-3.38 (-6.48, -0.28)	0.033
**Birth sex * ethnic group * sexual orientation**								

Female * Mixed * sexual minority	-0.09 (-3.07, 2.89)	0.951	-0.43 (-2.55, 1.70)	0.693	-1.31 (-4.15, 1.53)	0.366	-0.39 (-2.47, 1.68)	0.710
Female * South Asian * sexual minority	-0.50 (-2.55, 1.55)	0.631	-1.86 (-4.58, 0.86)	0.179	-1.74 (-3.64, 0.15)	0.071	-1.33 (-3.08, 0.42)	0.137
Female * Black * sexual minority	3.58 (-0.84, 8.00)	0.112	1.62 (-0.29, 3.52)	0.096	-3.01 (-7.22, 1.21)	0.162	-5.42 (-7.98, -2.86)	<0.001
Female * Other * sexual minority	-2.99 (-6.07, 0.08)	0.056	-2.51 (-5.06, 0.04)	0.054	-1.59 (-4.53, 1.34)	0.287	0.01 (-3.77, 3.79)	0.997
**Cohort * birth sex * ethnic group * sexual orientation**								
MCS * Female * Mixed * sexual minority	-2.02 (-5.84, 1.81)	0.301	-0.39 (-3.72, 2.94)	0.818	0.75 (-2.90, 4.39)	0.688	1.22 (-2.27, 4.72)	0.492
MCS * Female * South Asian * sexual minority	-1.34 (-5.52, 2.84)	0.530	1.35 (-2.26, 4.96)	0.464	2.78 (-1.17, 6.73)	0.168	2.32 (-0.85, 5.50)	0.151
MCS * Female * Black * sexual minority	-3.33 (-9.49, 2.83)	0.290	-0.40 (-2.88, 2.09)	0.754	4.90 (-0.97, 10.78)	0.102	8.32 (5.16, 11.48)	<0.001
MCS * Female * Other * sexual minority	3.40 (-2.18, 8.99)	0.233	5.85 (3.09, 8.61)	<0.001	1.88 (-3.44, 7.21)	0.488	3.44 (0.69, 6.19)	0.014
**Cohort * residential IMD rank**								
MCS * less deprived	0.09 (-0.21, 0.38)	0.564	0.11 (-0.40, 0.63)	0.666	0.07 (-0.21, 0.35)	0.627	0.12 (-0.39, 0.63)	0.640
**Birth sex * residential IMD rank**								
Female * less deprived	0.00 (-0.27, 0.28)	0.978	-0.06 (-0.58, 0.46)	0.816	-0.03 (-0.29, 0.23)	0.808	-0.06 (-0.50, 0.37)	0.770
**Cohort * birth sex * residential IMD rank**								
MCS * female * less deprived	0.01 (-0.37, 0.39)	0.955	-0.08 (-0.76, 0.61)	0.825	-0.07 (-0.44, 0.29)	0.692	-0.35 (-0.99, 0.29)	0.277
**Ethnic group * residential IMD rank**								
Mixed * Less deprived	0.03 (-0.96, 1.02)	0.956	-0.23 (-1.30, 0.83)	0.666	0.00 (-0.93, 0.94)	0.997	-0.39 (-1.38, 0.61)	0.447
South Asian * Less deprived	0.81 (0.26, 1.37)	0.004	1.01 (0.12, 1.89)	0.025	0.74 (0.20, 1.27)	0.007	0.67 (-0.03, 1.36)	0.060
Black * Less deprived	0.55 (-0.62, 1.73)	0.354	-0.31 (-1.42, 0.79)	0.578	0.25 (-0.87, 1.36)	0.665	-0.66 (-1.62, 0.29)	0.170
Other * Less deprived	-0.34 (-1.76, 1.07)	0.636	-1.70 (-3.43, 0.02)	0.053	-1.53 (-2.88, -0.18)	0.027	-2.58 (-4.19, -0.97)	0.002
**Cohort * ethnic group * residential IMD rank**								
MCS * Mixed * Less deprived	0.55 (-0.80, 1.89)	0.427	0.96 (-0.56, 2.49)	0.215	0.14 (-1.14, 1.42)	0.830	0.22 (-1.84, 2.29)	0.830
MCS * South Asian * Less deprived	-0.69 (-1.58, 0.20)	0.127	-0.90 (-1.97, 0.17)	0.098	-0.50 (-1.35, 0.35)	0.253	-0.10 (-1.26, 1.07)	0.870
MCS * Black * Less deprived	0.14 (-1.61, 1.88)	0.879	1.10 (-0.52, 2.72)	0.183	0.67 (-0.99, 2.34)	0.428	1.50 (-0.17, 3.16)	0.078
MCS * Other * Less deprived	0.06 (-1.86, 1.98)	0.950	0.95 (-1.62, 3.52)	0.469	0.89 (-0.94, 2.73)	0.339	1.66 (-0.07, 3.39)	0.060
**Birth sex * ethnic group * residential IMD rank**								
Female * Mixed * Less deprived	0.72 (-0.48, 1.91)	0.241	0.12 (-1.50, 1.75)	0.881	0.86 (-0.28, 1.99)	0.139	0.24 (-1.34, 1.81)	0.766
Female * South Asian * Less deprived	-0.45 (-1.16, 0.26)	0.210	-0.29 (-1.40, 0.82)	0.609	-0.50 (-1.18, 0.18)	0.150	-0.32 (-1.15, 0.51)	0.448
Female * Black * Less deprived	-0.64 (-2.08, 0.81)	0.387	0.62 (-1.44, 2.69)	0.554	0.05 (-1.33, 1.42)	0.946	2.28 (-0.08, 4.64)	0.058
Female * Other * Less deprived	-0.47 (-2.15, 1.21)	0.585	0.54 (-1.38, 2.46)	0.581	0.64 (-0.96, 2.24)	0.433	1.80 (-0.15, 3.75)	0.071
**Cohort * birth sex * ethnic group * residential IMD rank**								
MCS * Female * Mixed * Less deprived	-1.52 (-3.20, 0.16)	0.076	-0.94 (-3.15, 1.26)	0.403	-0.81 (-2.41, 0.78)	0.317	-0.22 (-2.78, 2.34)	0.868
MCS * Female * South Asian * Less deprived	0.18 (-0.97, 1.33)	0.758	-0.14 (-1.79, 1.51)	0.868	0.20 (-0.89, 1.30)	0.716	-1.39 (-3.67, 0.88)	0.230
MCS * Female * Black * Less deprived	-0.25 (-2.45, 1.95)	0.826	-1.04 (-3.88, 1.80)	0.474	-0.42 (-2.52, 1.68)	0.696	-2.35 (-5.70, 0.99)	0.167
MCS * Female * Other * Less deprived	2.02 (-0.31, 4.35)	0.090	2.71 (-0.24, 5.66)	0.072	0.92 (-1.30, 3.14)	0.418	0.90 (-1.43, 3.22)	0.449
**Sexual orientation * residential IMD rank**								
Sexual minority * Less deprived	-0.17 (-0.94, 0.60)	0.671	-0.05 (-1.15, 1.05)	0.928	-0.04 (-0.78, 0.69)	0.910	-0.59 (-1.81, 0.63)	0.346
**Cohort * sexual orientation * residential IMD rank**								
MCS * sexual minority * Less deprived	0.62 (-0.39, 1.63)	0.230	-0.48 (-2.02, 1.07)	0.543	0.44 (-0.52, 1.40)	0.370	0.47 (-1.09, 2.03)	0.554
**Birth sex * sexual orientation * residential IMD rank**								
Female * sexual minority * Less deprived	0.34 (-0.67, 1.35)	0.513	1.29 (-0.37, 2.96)	0.128	-0.16 (-1.12, 0.81)	0.749	1.51 (-0.12, 3.14)	0.069
**Cohort * birth sex * sexual orientation * residential IMD rank**								
MCS * Female * sexual minority * Less deprived	-1.02 (-2.29, 0.26)	0.117	-0.20 (-2.39, 1.99)	0.859	-0.41 (-1.63, 0.80)	0.506	-0.91 (-2.99, 1.16)	0.389
**Ethnic group * sexual orientation * residential IMD rank**								
Mixed * sexual minority * Less deprived	-3.52 (-7.84, 0.80)	0.110	-2.03 (-6.70, 2.64)	0.395	-2.88 (-7.00, 1.24)	0.171	-4.43 (-8.46, -0.40)	0.031
South Asian * sexual minority * Less deprived	-1.62 (-3.73, 0.50)	0.134	-1.21 (-3.75, 1.33)	0.349	-1.82 (-3.83, 0.20)	0.077	-0.19 (-2.09, 1.72)	0.849
Black * sexual minority * Less deprived	2.58 (-1.38, 6.54)	0.201	1.45 (-0.89, 3.78)	0.225	-1.63 (-5.40, 2.15)	0.399	-3.70 (-6.30, -1.11)	0.005
Other * sexual minority * Less deprived	-0.65 (-3.86, 2.56)	0.692	0.89 (-1.74, 3.53)	0.507	0.76 (-2.31, 3.82)	0.629	2.90 (-0.93, 6.72)	0.137
**Cohort * ethnic group * sexual orientation * residential IMD rank**								
MCS * Mixed * sexual minority * Less deprived	0.25 (-2.86, 3.35)	0.876	0.57 (-3.06, 4.19)	0.760	0.45 (-2.51, 3.42)	0.763	2.85 (-0.48, 6.18)	0.093

MCS * South Asian * sexual minority * Less deprived	-0.12 (-4.58, 4.34)	0.958	1.11 (-1.86, 4.07)	0.464	2.04 (-2.21, 6.30)	0.347	2.13 (-1.18, 5.44)	0.208
MCS * Black * sexual minority * Less deprived	-3.64 (-10.05, 2.77)	0.266	-1.70 (-4.56, 1.15)	0.242	3.42 (-2.69, 9.54)	0.272	5.94 (2.85, 9.02)	<0.001
MCS * Other * sexual minority * Less deprived	-1.08 (-6.18, 4.02)	0.678	-1.99 (-4.77, 0.80)	0.161	-1.50 (-6.37, 3.36)	0.544	-1.07 (-3.68, 1.55)	0.423
**Birth sex * ethnic group * sexual orientation * residential IMD rank**								
Female * Mixed * sexual minority * Less deprived	3.77 (0.32, 7.23)	0.032	1.69 (-2.51, 5.89)	0.430	3.27 (-0.02, 6.57)	0.052	3.41 (0.09, 6.73)	0.044
Female * South Asian * sexual minority * Less deprived	1.61 (-2.69, 5.90)	0.463	-1.15 (-4.53, 2.23)	0.505	-1.24 (-5.34, 2.85)	0.551	-2.72 (-6.70, 1.26)	0.180
Female * Black * sexual minority * Less deprived								
Female * Other * sexual minority * Less deprived	-0.54 (-5.75, 4.66)	0.839	-3.07 (-6.34, 0.20)	0.065	-1.78 (-6.75, 3.18)	0.481	-5.12 (-9.27, -0.98)	0.015
**Intercept**	1.30 (1.14, 1.46)	<0.001	1.22 (0.95, 1.49)	<0.001	1.37 (1.22, 1.52)	<0.001	1.28 (1.03, 1.52)	<0.001

	**UCLA-3**				**ONS life satisfaction**			
	**Unweighted**		**Weighted**		**Unweighted**		**Weighted**	
	**Coefficient (95% CI)**	***p***	**Coefficient (95% CI)**	***p***	**Coefficient (95% CI)**	***p***	**Coefficient (95% CI)**	***p***
**Cohort (MCS)**	0.44 (0.21, 0.66)	<0.001	0.38 (0.03, 0.73)	0.033	-0.46 (-0.74, -0.19)	0.001	-0.51 (-0.97, -0.06)	0.027
**Birth sex (female)**	0.29 (0.09, 0.49)	0.005	0.37 (0.09, 0.66)	0.011	-0.11 (-0.36, 0.13)	0.362	-0.31 (-0.67, 0.05)	0.090
**Ethnic group**								
Mixed	0.35 (-0.33, 1.04)	0.311	0.66 (0.09, 1.23)	0.023	-0.62 (-1.47, 0.22)	0.147	-1.17 (-2.58, 0.24)	0.103
South Asian	-0.11 (-0.43, 0.20)	0.470	0.17 (-0.55, 0.90)	0.635	0.38 (-0.01, 0.76)	0.055	0.32 (-0.27, 0.91)	0.292
Black	0.14 (-0.49, 0.76)	0.669	0.66 (-0.40, 1.71)	0.220	0.29 (-0.46, 1.05)	0.448	-1.36 (-3.24, 0.51)	0.154
Other	0.66 (-0.29, 1.62)	0.175	1.57 (-0.58, 3.73)	0.152	-1.36 (-2.54, -0.18)	0.024	-1.83 (-3.54, -0.12)	0.036
**Sexual orientation (sexual minority)**	0.73 (0.24, 1.22)	0.004	0.72 (-0.07, 1.52)	0.075	-0.85 (-1.46, -0.24)	0.006	-0.69 (-2.26, 0.88)	0.388
**Residential IMD rank (Less deprived)**	-0.20 (-0.42, 0.01)	0.064	0.04 (-0.38, 0.45)	0.867	0.37 (0.10, 0.63)	0.006	0.04 (-0.49, 0.57)	0.884
**Cohort * birth sex**								
MCS * female	0.03 (-0.26, 0.32)	0.849	0.13 (-0.35, 0.60)	0.592	-0.17 (-0.52, 0.19)	0.355	-0.25 (-0.90, 0.40)	0.452
**Cohort * ethnic group**								
MCS * Mixed	-0.44 (-1.34, 0.47)	0.342	0.12 (-1.00, 1.24)	0.835	0.84 (-0.28, 1.95)	0.142	1.14 (-0.36, 2.65)	0.135
MCS * South Asian	-0.53 (-1.01, -0.05)	0.029	-0.73 (-1.58, 0.12)	0.091	-0.12 (-0.71, 0.47)	0.688	-0.09 (-0.99, 0.82)	0.854
MCS * Black	-0.69 (-1.56, 0.18)	0.119	-1.04 (-2.49, 0.42)	0.163	-0.18 (-1.24, 0.88)	0.740	0.70 (-1.69, 3.09)	0.565
MCS * Other	-0.63 (-1.89, 0.62)	0.323	-0.41 (-2.89, 2.06)	0.743	1.58 (0.06, 3.10)	0.041	0.22 (-2.26, 2.71)	0.860
**Birth sex * ethnic group**								
Female * Mixed	-0.30 (-1.11, 0.51)	0.467	-0.14 (-1.26, 0.99)	0.814	0.94 (-0.06, 1.94)	0.064	0.90 (-0.96, 2.75)	0.342
Female * South Asian	0.14 (-0.26, 0.54)	0.488	-0.23 (-1.05, 0.58)	0.576	-0.51 (-1.01, -0.02)	0.041	-0.15 (-0.86, 0.55)	0.668
Female * Black	-0.31 (-1.05, 0.43)	0.414	-0.65 (-2.07, 0.76)	0.365	-0.44 (-1.34, 0.46)	0.340	0.95 (-1.02, 2.92)	0.345
Female * Other	0.02 (-1.08, 1.13)	0.970	-1.32 (-3.81, 1.16)	0.296	0.94 (-0.43, 2.30)	0.180	1.96 (0.02, 3.91)	0.048
**Cohort * birth sex * ethnic group**								
MCS * Female * Mixed	0.16 (-0.95, 1.28)	0.778	-0.81 (-2.45, 0.83)	0.330	-1.38 (-2.76, -0.01)	0.049	-1.77 (-3.91, 0.36)	0.103
MCS * Female * South Asian	0.39 (-0.24, 1.01)	0.222	1.68 (-0.35, 3.71)	0.105	0.25 (-0.51, 1.01)	0.522	-0.53 (-2.35, 1.30)	0.572
MCS * Female * Black	0.89 (-0.20, 1.99)	0.111	0.71 (-1.32, 2.73)	0.494	0.72 (-0.62, 2.07)	0.293	0.51 (-2.19, 3.20)	0.712
MCS * Female * Other	-0.28 (-1.77, 1.21)	0.708	-0.31 (-3.17, 2.56)	0.833	-1.52 (-3.33, 0.30)	0.101	0.02 (-2.79, 2.83)	0.990
**Cohort * sexual orientation**								
MCS * sexual minority	0.33 (-0.36, 1.02)	0.348	0.49 (-0.60, 1.58)	0.380	0.09 (-0.76, 0.94)	0.833	-0.51 (-2.36, 1.34)	0.590
**Birth sex * sexual orientation**								
Female * sexual minority	0.28 (-0.41, 0.97)	0.427	0.04 (-1.17, 1.26)	0.946	-0.30 (-1.15, 0.55)	0.489	-0.09 (-2.04, 1.86)	0.926
**Cohort * birth sex * sexual orientation**								
MCS * Female * sexual minority	-0.52 (-1.42, 0.37)	0.251	-0.75 (-2.43, 0.94)	0.384	0.22 (-0.88, 1.33)	0.690	0.81 (-1.45, 3.06)	0.484
**Ethnic group * sexual orientation**								
Mixed * sexual minority	1.27 (-1.27, 3.81)	0.328	1.70 (0.45, 2.94)	0.007	0.89 (-2.25, 4.03)	0.579	1.11 (-0.97, 3.20)	0.296
South Asian * sexual minority	1.02 (-0.38, 2.42)	0.152	0.58 (-1.69, 2.84)	0.617	-0.97 (-2.70, 0.76)	0.272	-0.64 (-3.38, 2.09)	0.644
Black * sexual minority	-1.82 (-7.15, 3.52)	0.504	1.86 (-0.99, 4.70)	0.201	-2.59 (-8.46, 3.28)	0.387	-4.09 (-7.34, -0.85)	0.013

Other * sexual minority	-0.37 (-2.60, 1.86)	0.743	-0.54 (-4.94, 3.85)	0.809	-1.05 (-3.80, 1.71)	0.456	-1.22 (-6.14, 3.69)	0.625
**Cohort * ethnic group * sexual orientation**								
MCS * Mixed * sexual minority	-1.66 (-4.96, 1.63)	0.322	-2.69 (-5.15, -0.23)	0.032	-1.46 (-5.53, 2.61)	0.481	-1.61 (-4.77, 1.56)	0.320
MCS * South Asian * sexual minority	0.48 (-3.24, 4.20)	0.801	0.84 (-1.58, 3.25)	0.496	-4.65 (-9.25, -0.05)	0.047	-4.63 (-7.59, -1.67)	0.002
MCS * Black * sexual minority	0.80 (-4.29, 5.90)	0.758	0.34 (-1.74, 2.42)	0.751	7.11 (-0.16, 14.39)	0.055	9.72 (6.04, 13.40)	<0.001
MCS * Other * sexual minority	1.03 (-5.13, 7.19)	0.743	-2.23 (-6.46, 1.99)	0.300	-2.12 (-9.73, 5.49)	0.585	-2.25 (-6.06, 1.55)	0.246
**Birth sex * ethnic group * sexual orientation**								
Female * Mixed * sexual minority	-1.93 (-4.90, 1.03)	0.202	-2.60 (-4.71, -0.50)	0.015	-0.70 (-4.36, 2.96)	0.709	-0.64 (-3.85, 2.56)	0.694
Female * South Asian * sexual minority	-0.99 (-3.03, 1.05)	0.340	0.17 (-2.40, 2.73)	0.899	0.79 (-1.65, 3.22)	0.528	-0.21 (-4.41, 4.00)	0.923
Female * Black * sexual minority	1.30 (-3.72, 6.32)	0.613	-1.39 (-3.57, 0.79)	0.212	3.42 (-2.01, 8.85)	0.218	4.93 (2.22, 7.65)	<0.001
Female * Other * sexual minority	-0.25 (-3.31, 2.80)	0.871	0.30 (-4.55, 5.15)	0.903	1.48 (-2.30, 5.26)	0.443	0.71 (-4.71, 6.13)	0.798
**Cohort * birth sex * ethnic group * sexual orientation**								
MCS * Female * Mixed * sexual minority	2.50 (-1.30, 6.30)	0.197	4.44 (1.05, 7.83)	0.010	1.24 (-3.46, 5.94)	0.605	1.20 (-3.38, 5.78)	0.607
MCS * Female * South Asian * sexual minority	-0.06 (-4.22, 4.09)	0.976	-2.45 (-6.35, 1.45)	0.218	4.75 (-0.35, 9.84)	0.068	6.78 (1.82, 11.74)	0.007
MCS * Female * Black * sexual minority	-2.53 (-8.47, 3.42)	0.404	-2.42 (-5.49, 0.64)	0.121	-9.34 (-16.91, -1.77)	0.016	-12.61 (-16.03, -9.19)	<0.001
MCS * Female * Other * sexual minority	-1.19 (-6.74, 4.37)	0.676	2.28 (-1.33, 5.88)	0.215	2.44 (-4.43, 9.30)	0.486	2.59 (-0.54, 5.72)	0.105
**Cohort * residential IMD rank**								
MCS * less deprived	0.23 (-0.07, 0.52)	0.130	0.19 (-0.33, 0.71)	0.476	-0.28 (-0.64, 0.08)	0.133	-0.24 (-0.91, 0.44)	0.490
**Birth sex * residential IMD rank**								
Female * less deprived	0.10 (-0.17, 0.37)	0.473	-0.03 (-0.52, 0.46)	0.904	-0.21 (-0.54, 0.13)	0.230	0.20 (-0.41, 0.81)	0.521
**Cohort * birth sex * residential IMD rank**								
MCS * female * less deprived	-0.34 (-0.72, 0.04)	0.083	-0.59 (-1.25, 0.07)	0.080	0.30 (-0.17, 0.77)	0.207	0.44 (-0.42, 1.30)	0.313
**Ethnic group * residential IMD rank**								
Mixed * Less deprived	0.08 (-0.89, 1.06)	0.867	-0.72 (-1.83, 0.39)	0.202	0.15 (-1.06, 1.35)	0.808	1.15 (-0.63, 2.93)	0.205
South Asian * Less deprived	0.67 (0.11, 1.23)	0.018	-0.06 (-1.18, 1.06)	0.913	-1.17 (-1.85, -0.49)	0.001	-0.74 (-1.92, 0.44)	0.219
Black * Less deprived	-0.21 (-1.42, 0.99)	0.728	-1.15 (-2.60, 0.29)	0.117	-0.16 (-1.60, 1.27)	0.822	2.04 (-0.48, 4.55)	0.113
Other * Less deprived	-0.38 (-1.79, 1.03)	0.600	-1.68 (-4.00, 0.65)	0.158	0.76 (-0.98, 2.50)	0.389	0.99 (-0.87, 2.86)	0.298
**Cohort * ethnic group * residential IMD rank**								
MCS * Mixed * Less deprived	-0.49 (-1.82, 0.83)	0.466	-0.08 (-1.87, 1.71)	0.931	-0.57 (-2.21, 1.06)	0.493	-1.98 (-4.46, 0.51)	0.119
MCS * South Asian * Less deprived	0.09 (-0.79, 0.97)	0.841	0.70 (-0.65, 2.04)	0.312	0.70 (-0.38, 1.79)	0.204	-0.11 (-1.59, 1.36)	0.879
MCS * Black * Less deprived	0.66 (-1.11, 2.43)	0.464	0.86 (-1.20, 2.93)	0.413	-1.38 (-3.53, 0.76)	0.207	-2.90 (-6.88, 1.07)	0.152
MCS * Other * Less deprived	0.51 (-1.41, 2.44)	0.601	1.67 (-1.43, 4.77)	0.290	-1.29 (-3.65, 1.06)	0.282	1.13 (-1.61, 3.87)	0.419
**Birth sex * ethnic group * residential IMD rank**								
Female * Mixed * Less deprived	0.10 (-1.08, 1.28)	0.869	-0.24 (-1.85, 1.36)	0.766	-1.10 (-2.56, 0.36)	0.139	-1.37 (-3.81, 1.07)	0.269
Female * South Asian * Less deprived	-0.81 (-1.52, -0.10)	0.025	0.22 (-1.15, 1.58)	0.756	1.39 (0.52, 2.25)	0.002	0.67 (-0.66, 2.00)	0.325
Female * Black * Less deprived	0.29 (-1.18, 1.75)	0.703	2.00 (-0.23, 4.22)	0.078	-0.10 (-1.87, 1.67)	0.915	-2.66 (-5.35, 0.03)	0.053
Female * Other * Less deprived	-0.68 (-2.35, 0.99)	0.427	1.25 (-1.55, 4.05)	0.380	-0.23 (-2.29, 1.83)	0.828	-1.66 (-4.11, 0.78)	0.183
**Cohort * birth sex * ethnic group * residential IMD rank**								
MCS * Female * Mixed * Less deprived	1.17 (-0.49, 2.83)	0.167	1.95 (-0.40, 4.30)	0.104	1.44 (-0.60, 3.49)	0.167	2.53 (-0.72, 5.78)	0.127
MCS * Female * South Asian * Less deprived	0.30 (-0.84, 1.45)	0.601	-1.58 (-4.25, 1.09)	0.245	-0.97 (-2.38, 0.43)	0.173	0.38 (-2.25, 3.01)	0.776
MCS * Female * Black * Less deprived	-0.48 (-2.69, 1.73)	0.670	-1.26 (-4.32, 1.81)	0.422	1.22 (-1.48, 3.92)	0.376	3.34 (-1.26, 7.94)	0.154
MCS * Female * Other * Less deprived	1.14 (-1.19, 3.47)	0.338	-0.08 (-3.66, 3.50)	0.964	-0.56 (-3.43, 2.30)	0.700	-2.89 (-6.28, 0.51)	0.096
**Sexual orientation * residential IMD rank**								
Sexual minority * Less deprived	0.47 (-0.30, 1.23)	0.233	-0.58 (-2.03, 0.87)	0.433	-0.77 (-1.72, 0.17)	0.109	-0.34 (-2.52, 1.84)	0.761
**Cohort * sexual orientation * residential IMD rank**								
MCS * sexual minority * Less deprived	-0.77 (-1.77, 0.23)	0.133	-0.22 (-2.10, 1.66)	0.817	0.77 (-0.46, 2.01)	0.221	0.71 (-1.78, 3.21)	0.574
**Birth sex * sexual orientation * residential IMD rank**								
Female * sexual minority * Less deprived	-0.94 (-1.95, 0.07)	0.067	0.66 (-1.27, 2.58)	0.504	1.24 (0.00, 2.48)	0.051	0.23 (-2.50, 2.97)	0.867
**Cohort * birth sex * sexual orientation * residential IMD rank**								
MCS * Female * sexual minority * Less deprived	1.16 (-0.10, 2.43)	0.072	0.67 (-1.78, 3.12)	0.591	-1.05 (-2.61, 0.51)	0.187	-1.03 (-4.11, 2.04)	0.510

**Ethnic group * sexual orientation * residential IMD rank**								
Mixed * sexual minority * Less deprived	-0.07 (-4.37, 4.22)	0.973	1.42 (-2.58, 5.42)	0.487	-0.02 (-5.33, 5.28)	0.993	3.10 (-2.39, 8.58)	0.268
South Asian * sexual minority * Less deprived	-2.05 (-4.16, 0.05)	0.056	0.13 (-2.79, 3.05)	0.931	1.10 (-1.50, 3.69)	0.407	-2.40 (-5.75, 0.94)	0.159
Black * sexual minority * Less deprived	1.26 (-2.68, 5.20)	0.532	-1.09 (-3.53, 1.35)	0.381	1.63 (-3.24, 6.50)	0.512	2.40 (0.13, 4.68)	0.039
Other * sexual minority * Less deprived	0.45 (-2.75, 3.64)	0.783	1.92 (-2.81, 6.64)	0.426	2.97 (-0.98, 6.92)	0.140	2.54 (-2.65, 7.73)	0.337
**Cohort * ethnic group * sexual orientation * residential IMD rank**								
MCS * Mixed * sexual minority * Less deprived	-0.57 (-3.66, 2.52)	0.717	-1.26 (-4.32, 1.80)	0.420	2.36 (-1.46, 6.17)	0.226	0.41 (-3.92, 4.74)	0.853
MCS * South Asian * sexual minority * Less deprived	-0.18 (-4.62, 4.26)	0.937	-1.55 (-4.90, 1.81)	0.367	3.28 (-2.20, 8.76)	0.241	7.51 (3.73, 11.29)	<0.001
MCS * Black * sexual minority * Less deprived					-6.17 (-14.05, 1.70)	0.124	-7.71 (-11.73, -3.70)	<0.001
MCS * Other * sexual minority * Less deprived	-2.64 (-7.71, 2.44)	0.308	-2.73 (-6.05, 0.58)	0.106	2.55 (-3.72, 8.81)	0.426	2.25 (-0.72, 5.22)	0.138
**Birth sex * ethnic group * sexual orientation * residential IMD rank**								
Female * Mixed * sexual minority * Less deprived	0.80 (-2.63, 4.24)	0.647	0.03 (-3.43, 3.49)	0.988	-2.15 (-6.40, 2.09)	0.320	-4.50 (-9.05, 0.06)	0.053
Female * South Asian * sexual minority * Less deprived	0.47 (-3.80, 4.74)	0.828	0.67 (-2.96, 4.29)	0.718	-2.73 (-8.00, 2.54)	0.310	-4.90 (-8.92, -0.87)	0.017
Female * Black * sexual minority * Less deprived								
Female * Other * sexual minority * Less deprived	1.18 (-4.00, 6.36)	0.654	-1.28 (-6.55, 4.00)	0.635	-2.47 (-8.87, 3.92)	0.448	-0.28 (-6.02, 5.46)	0.924
**Intercept**	4.65 (4.49, 4.80)	<0.001	4.55 (4.31, 4.79)	<0.001	6.59 (6.39, 6.78)	<0.001	6.75 (6.46, 7.04)	<0.001

Note. CI: confidence interval; GAD-2: 2-item Generalised Anxiety Disorder questionnaire; MCS: Millennium Cohort Study (born in 2000-2002); NS: Next Steps study (born in 1990); ONS: Office for National Statistics; PHQ-2: 2-item depression Patient Health Questionnaire; UCLA-3: 3-item University of California Los Angeles loneliness scale.

Weighted results are adjusted for survey design characteristics, including survey and non-response weights. White includes all White groups; South Asian includes Bangladeshi, Indian, and Pakistani groups; Black includes Black African, Black Caribbean, and Black British groups; Other includes all other ethnic group not included in the other categories.

Table S8. Results from the fixed-effects multiple regression approach using stratification 80b (cohort * birth sex * ethnic group * sexual orientation * housing tenure).

	GAD-2				PHQ-2			
	Unweighted		Weighted		Unweighted		Weighted	
	Coefficient (95% CI)	*p*	Coefficient (95% CI)	*p*	Coefficient (95% CI)	*p*	Coefficient (95% CI)	*p*
**Cohort (MCS)**	0.07 (-0.15, 0.28)	0.536	0.14 (-0.25, 0.54)	0.473	0.04 (-0.17, 0.24)	0.707	0.12 (-0.26, 0.49)	0.541
**Birth sex (female)**	0.73 (0.51, 0.94)	<0.001	0.78 (0.36, 1.20)	<0.001	0.33 (0.13, 0.53)	0.002	0.48 (0.11, 0.84)	0.011
**Ethnic group**								
Mixed	0.12 (-0.60, 0.83)	0.747	-0.20 (-1.13, 0.73)	0.676	-0.14 (-0.80, 0.52)	0.681	-0.09 (-0.88, 0.69)	0.815
South Asian	-0.03 (-0.45, 0.39)	0.890	-0.04 (-0.61, 0.54)	0.905	-0.20 (-0.60, 0.20)	0.323	-0.38 (-0.90, 0.13)	0.143
Black	-0.74 (-1.44, -0.05)	0.037	-0.03 (-1.01, 0.94)	0.945	-0.50 (-1.16, 0.16)	0.141	-0.06 (-0.82, 0.70)	0.881
Other	-0.34 (-1.33, 0.65)	0.501	0.38 (-1.28, 2.05)	0.652	-0.04 (-0.98, 0.91)	0.935	0.75 (-1.19, 2.69)	0.447
**Sexual orientation (sexual minority)**	0.43 (-0.08, 0.95)	0.098	-0.23 (-1.02, 0.57)	0.573	0.56 (0.08, 1.05)	0.024	0.87 (0.22, 1.52)	0.009
**Housing tenure (homeowner/part owner)**	-0.41 (-0.63, -0.19)	<0.001	-0.43 (-0.83, -0.02)	0.040	-0.49 (-0.70, -0.28)	<0.001	-0.57 (-0.90, -0.25)	0.001
**Cohort * birth sex**								
MCS * female	0.31 (0.03, 0.59)	0.029	0.27 (-0.25, 0.79)	0.310	0.25 (-0.02, 0.51)	0.069	0.07 (-0.43, 0.56)	0.795
**Cohort * ethnic group**								
MCS * Mixed	-0.23 (-1.21, 0.76)	0.654	0.67 (-0.71, 2.04)	0.341	-0.09 (-1.02, 0.83)	0.844	0.38 (-1.12, 1.88)	0.616
MCS * South Asian	-0.37 (-1.01, 0.28)	0.262	-0.72 (-1.40, -0.04)	0.037	-0.04 (-0.65, 0.57)	0.905	-0.09 (-0.89, 0.72)	0.835
MCS * Black	0.51 (-0.46, 1.48)	0.300	-0.36 (-1.50, 0.78)	0.536	0.51 (-0.41, 1.43)	0.274	0.49 (-0.72, 1.71)	0.427
MCS * Other	1.03 (-0.40, 2.46)	0.159	1.42 (-0.63, 3.46)	0.174	0.64 (-0.72, 2.01)	0.355	-0.42 (-2.40, 1.55)	0.673
**Birth sex * ethnic group**								
Female * Mixed	-0.41 (-1.25, 0.43)	0.341	0.19 (-1.23, 1.61)	0.793	-0.01 (-0.80, 0.78)	0.981	0.11 (-1.22, 1.45)	0.866
Female * South Asian	-0.33 (-0.85, 0.20)	0.222	-0.28 (-1.00, 0.44)	0.448	-0.13 (-0.63, 0.36)	0.598	-0.24 (-0.87, 0.39)	0.463
Female * Black	-0.09 (-0.94, 0.76)	0.833	-0.58 (-1.77, 0.62)	0.345	-0.05 (-0.86, 0.75)	0.896	-0.69 (-1.62, 0.24)	0.147
Female * Other	-0.43 (-1.58, 0.72)	0.462	-0.77 (-2.76, 1.21)	0.443	-0.50 (-1.60, 0.59)	0.365	-1.22 (-3.26, 0.83)	0.244
**Cohort * birth sex * ethnic group**								
MCS * Female * Mixed	0.42 (-0.76, 1.60)	0.482	-0.67 (-2.57, 1.23)	0.489	0.27 (-0.84, 1.38)	0.637	-0.16 (-2.09, 1.78)	0.872
MCS * Female * South Asian	0.80 (-0.02, 1.62)	0.056	1.16 (0.03, 2.29)	0.045	0.69 (-0.09, 1.47)	0.082	0.85 (-0.18, 1.88)	0.107
MCS * Female * Black	0.18 (-1.02, 1.38)	0.768	0.75 (-0.88, 2.38)	0.366	-0.07 (-1.21, 1.08)	0.909	-0.17 (-1.94, 1.61)	0.855
MCS * Female * Other	-0.92 (-2.59, 0.75)	0.279	-1.39 (-3.90, 1.13)	0.279	0.21 (-1.37, 1.80)	0.791	1.06 (-1.18, 3.30)	0.354
**Cohort * sexual orientation**								
MCS * sexual minority	0.42 (-0.23, 1.08)	0.202	1.01 (-0.09, 2.11)	0.072	0.35 (-0.27, 0.97)	0.274	-0.08 (-0.93, 0.78)	0.862
**Birth sex * sexual orientation**								
Female * sexual minority	0.05 (-0.62, 0.73)	0.877	0.38 (-0.96, 1.71)	0.583	0.16 (-0.48, 0.80)	0.624	-0.44 (-1.51, 0.62)	0.415
**Cohort * birth sex * sexual orientation**								
MCS * Female * sexual minority	-0.36 (-1.19, 0.46)	0.390	-0.56 (-2.17, 1.05)	0.492	-0.30 (-1.09, 0.48)	0.448	0.43 (-0.85, 1.71)	0.512
**Ethnic group * sexual orientation**								
Mixed * sexual minority	0.03 (-3.48, 3.53)	0.989	1.07 (-0.11, 2.25)	0.076	1.04 (-2.29, 4.36)	0.541	0.71 (-0.27, 1.69)	0.154
South Asian * sexual minority	0.67 (-0.85, 2.20)	0.388	2.71 (0.11, 5.31)	0.041	-0.23 (-1.69, 1.22)	0.753	0.53 (-1.51, 2.57)	0.611
Black * sexual minority	-0.11 (-3.61, 3.39)	0.950	-0.10 (-1.32, 1.12)	0.877	2.40 (-0.93, 5.72)	0.158	1.68 (0.72, 2.64)	0.001
Other * sexual minority	2.23 (0.21, 4.26)	0.031	3.62 (1.51, 5.73)	0.001	0.69 (-1.24, 2.61)	0.484	0.49 (-3.17, 4.16)	0.792
**Cohort * ethnic group * sexual orientation**								
MCS * Mixed * sexual minority	-0.60 (-4.69, 3.49)	0.773	-0.97 (-3.40, 1.46)	0.432	-1.96 (-5.84, 1.93)	0.323	-2.23 (-3.98, -0.48)	0.012
MCS * South Asian * sexual minority	-0.62 (-3.53, 2.29)	0.674	-2.33 (-5.36, 0.71)	0.133	-1.51 (-4.28, 1.25)	0.283	-2.03 (-4.30, 0.24)	0.080
MCS * Black * sexual minority	0.00 (-4.94, 4.93)	0.999	0.21 (-1.33, 1.75)	0.791	-4.90 (-9.59, -0.21)	0.041	-4.53 (-5.97, -3.09)	<0.001
MCS * Other * sexual minority	-2.87 (-8.01, 2.28)	0.275	-6.11 (-9.60, -2.62)	0.001	-5.07 (-9.96, -0.18)	0.042	-5.32 (-9.61, -1.02)	0.015
**Birth sex * ethnic group * sexual orientation**								
Female * Mixed * sexual minority	-1.12 (-5.06, 2.82)	0.577	-2.13 (-4.21, -0.05)	0.044	-2.23 (-5.97, 1.51)	0.243	-1.96 (-4.05, 0.13)	0.066

Female * South Asian * sexual minority	-0.62 (-3.16, 1.92)	0.632	-3.63 (-7.01, -0.24)	0.036	-0.77 (-3.00, 1.45)	0.497	-1.96 (-4.29, 0.37)	0.100
Female * Black * sexual minority	1.56 (-2.38, 5.50)	0.437	1.58 (-1.33, 4.48)	0.287	-1.94 (-5.68, 1.81)	0.311	-1.02 (-3.59, 1.56)	0.439
Female * Other * sexual minority	-2.60 (-5.33, 0.14)	0.063	-4.32 (-7.30, -1.34)	0.004	-0.98 (-3.59, 1.62)	0.458	-1.28 (-5.32, 2.76)	0.535
**Cohort * birth sex * ethnic group * sexual orientation**								
MCS * Female * Mixed * sexual minority	1.71 (-2.90, 6.32)	0.467	2.67 (-0.81, 6.14)	0.132	3.66 (-0.72, 8.05)	0.102	4.15 (1.08, 7.22)	0.008
MCS * Female * South Asian * sexual minority	-0.21 (-3.98, 3.57)	0.915	3.00 (-0.89, 6.90)	0.131	2.66 (-0.81, 6.12)	0.133	3.82 (0.74, 6.91)	0.015
MCS * Female * Black * sexual minority	-0.88 (-6.69, 4.93)	0.767	-0.95 (-4.18, 2.27)	0.562	4.13 (-1.40, 9.65)	0.143	3.94 (0.89, 6.99)	0.011
MCS * Female * Other * sexual minority	4.02 (-1.19, 9.22)	0.130	6.94 (3.09, 10.79)	<0.001	4.14 (-0.81, 9.09)	0.101	4.66 (0.20, 9.11)	0.040
**Cohort * housing tenure**								
MCS * homeowner/part owner	0.41 (0.11, 0.71)	0.008	0.27 (-0.24, 0.78)	0.295	0.43 (0.14, 0.72)	0.003	0.33 (-0.13, 0.80)	0.162
**Birth sex * housing tenure**								
Female * homeowner/part owner	-0.03 (-0.31, 0.25)	0.852	-0.02 (-0.54, 0.50)	0.943	-0.02 (-0.29, 0.25)	0.876	-0.05 (-0.46, 0.36)	0.820
**Cohort * birth sex * housing tenure**								
MCS * female * homeowner/part owner	-0.18 (-0.57, 0.22)	0.380	-0.16 (-0.82, 0.49)	0.625	-0.08 (-0.46, 0.29)	0.673	0.00 (-0.61, 0.62)	0.993
**Ethnic group * housing tenure**								
Mixed * homeowner/part owner	-0.26 (-1.28, 0.75)	0.610	-0.05 (-1.15, 1.06)	0.936	0.27 (-0.69, 1.22)	0.587	0.13 (-0.99, 1.25)	0.823
South Asian * homeowner/part owner	0.07 (-0.47, 0.60)	0.808	0.24 (-0.56, 1.04)	0.560	0.19 (-0.32, 0.70)	0.460	0.36 (-0.29, 1.00)	0.277
Black * homeowner/part owner	0.18 (-0.86, 1.22)	0.737	-0.68 (-1.71, 0.35)	0.195	0.35 (-0.64, 1.34)	0.487	0.04 (-1.19, 1.27)	0.948
Other * homeowner/part owner	0.14 (-1.29, 1.58)	0.845	0.91 (-1.59, 3.42)	0.473	-0.01 (-1.37, 1.35)	0.990	0.78 (-1.75, 3.30)	0.546
**Cohort * ethnic group * housing tenure**								
MCS * Mixed * homeowner/part owner	0.57 (-0.83, 1.97)	0.424	-0.15 (-1.79, 1.50)	0.862	0.68 (-0.65, 2.00)	0.316	1.25 (-0.80, 3.30)	0.232
MCS * South Asian * homeowner/part owner	0.10 (-0.75, 0.95)	0.824	0.44 (-0.61, 1.50)	0.407	-0.15 (-0.96, 0.65)	0.707	-0.01 (-1.16, 1.13)	0.983
MCS * Black * homeowner/part owner	-0.27 (-1.86, 1.31)	0.737	0.82 (-0.54, 2.19)	0.236	-0.22 (-1.73, 1.29)	0.776	-0.22 (-2.30, 1.85)	0.832
MCS * Other * homeowner/part owner	-1.14 (-3.19, 0.90)	0.273	-2.41 (-5.38, 0.57)	0.113	-1.11 (-3.06, 0.83)	0.262	-1.63 (-4.22, 0.96)	0.216
**Birth sex * ethnic group * housing tenure**								
Female * Mixed * homeowner/part owner	0.67 (-0.55, 1.89)	0.285	0.25 (-1.53, 2.03)	0.780	0.22 (-0.93, 1.38)	0.703	0.26 (-1.53, 2.04)	0.777
Female * South Asian * homeowner/part owner	0.11 (-0.57, 0.78)	0.756	-0.06 (-1.05, 0.94)	0.907	0.17 (-0.47, 0.81)	0.601	0.28 (-0.52, 1.08)	0.491
Female * Black * homeowner/part owner	0.12 (-1.15, 1.39)	0.857	1.81 (0.01, 3.60)	0.049	-0.08 (-1.29, 1.13)	0.899	1.35 (-0.57, 3.27)	0.169
Female * Other * homeowner/part owner	0.60 (-1.11, 2.30)	0.495	-0.06 (-2.86, 2.73)	0.965	0.83 (-0.79, 2.46)	0.315	0.27 (-2.39, 2.92)	0.842
**Cohort * birth sex * ethnic group * housing tenure**								
MCS * Female * Mixed * homeowner/part owner	-1.25 (-3.05, 0.54)	0.170	0.33 (-2.22, 2.89)	0.797	-1.00 (-2.69, 0.70)	0.251	-1.18 (-3.96, 1.60)	0.405
MCS * Female * South Asian * homeowner/part owner	-0.59 (-1.69, 0.51)	0.292	-0.32 (-2.18, 1.53)	0.732	-0.42 (-1.46, 0.63)	0.435	0.64 (-1.80, 3.07)	0.608
MCS * Female * Black * homeowner/part owner	-0.71 (-3.20, 1.79)	0.578	-2.28 (-4.48, -0.08)	0.043	-0.69 (-3.06, 1.68)	0.569	-1.92 (-4.78, 0.94)	0.189
MCS * Female * Other * homeowner/part owner	1.07 (-1.42, 3.57)	0.399	3.48 (-0.31, 7.27)	0.072	0.32 (-2.06, 2.69)	0.794	1.62 (-1.55, 4.80)	0.315
**Sexual orientation * housing tenure**								
Sexual minority * homeowner/part owner	0.31 (-0.43, 1.06)	0.411	0.46 (-0.67, 1.58)	0.429	-0.16 (-0.87, 0.55)	0.662	-0.62 (-1.61, 0.36)	0.214
**Cohort * sexual orientation * housing tenure**								
MCS * sexual minority * homeowner/part owner	-0.40 (-1.44, 0.64)	0.452	-0.31 (-1.85, 1.22)	0.690	0.41 (-0.58, 1.40)	0.422	0.63 (-0.65, 1.90)	0.336
**Birth sex * sexual orientation * housing tenure**								
Female * sexual minority * homeowner/part owner	-0.07 (-1.07, 0.94)	0.895	-0.08 (-1.75, 1.59)	0.925	0.12 (-0.83, 1.08)	0.801	0.69 (-0.74, 2.11)	0.344
**Cohort * birth sex * sexual orientation * housing tenure**								
MCS * Female * sexual minority * homeowner/part owner	0.31 (-1.01, 1.62)	0.646	-0.61 (-2.90, 1.68)	0.601	-0.46 (-1.72, 0.79)	0.467	-1.38 (-3.34, 0.58)	0.169
**Ethnic group * sexual orientation * housing tenure**								
Mixed * sexual minority * homeowner/part owner	-0.64 (-5.60, 4.32)	0.800	-0.98 (-2.51, 0.55)	0.207	-1.62 (-6.33, 3.10)	0.502	-0.93 (-2.37, 0.51)	0.205
South Asian * sexual minority * homeowner/part owner	-1.47 (-3.51, 0.57)	0.157	-2.48 (-5.31, 0.34)	0.085	0.04 (-1.90, 1.98)	0.967	0.00 (-2.25, 2.24)	0.997
Black * sexual minority * homeowner/part owner	-0.08 (-5.04, 4.88)	0.974	0.65 (-0.82, 2.13)	0.385	-0.70 (-5.42, 4.02)	0.771	0.16 (-1.38, 1.69)	0.843
Other * sexual minority * homeowner/part owner	-0.30 (-3.64, 3.05)	0.861	-1.60 (-4.75, 1.55)	0.320	1.91 (-1.27, 5.09)	0.240	0.79 (-3.27, 4.85)	0.703
**Cohort * ethnic group * sexual orientation * housing tenure**								
MCS * Mixed * sexual minority * homeowner/part owner	2.75 (-3.05, 8.55)	0.352	2.45 (-0.96, 5.86)	0.159	2.48 (-3.03, 8.00)	0.377	1.59 (-1.24, 4.43)	0.271
MCS * South Asian * sexual minority * homeowner/part owner	1.39 (-2.44, 5.22)	0.476	2.72 (-0.55, 5.99)	0.102	1.57 (-2.08, 5.21)	0.399	3.70 (0.72, 6.67)	0.015

MCS * Black * sexual minority * homeowner/part owner								
MCS * Other * sexual minority * homeowner/part owner	0.48 (-4.66, 5.61)	0.856	1.64 (-2.33, 5.62)	0.417	1.32 (-3.56, 6.20)	0.597	2.46 (-2.09, 7.01)	0.289
**Birth sex * ethnic group * sexual orientation * housing tenure**								
Female * Mixed * sexual minority * homeowner/part owner	1.93 (-3.66, 7.51)	0.498	2.34 (-0.57, 5.24)	0.115	2.42 (-2.89, 7.74)	0.371	1.87 (-1.11, 4.84)	0.218
Female * South Asian * sexual minority * homeowner/part owner	1.15 (-2.37, 4.68)	0.521	1.81 (-1.96, 5.58)	0.345	0.23 (-2.99, 3.45)	0.890	0.63 (-1.96, 3.23)	0.632
Female * Black * sexual minority * homeowner/part owner	-1.27 (-7.59, 5.05)	0.693	-2.87 (-6.19, 0.46)	0.091	0.48 (-5.54, 6.49)	0.876	-1.32 (-4.51, 1.88)	0.419
Female * Other * sexual minority * homeowner/part owner								
**Cohort * birth sex * ethnic group * sexual orientation * housing tenure**								
MCS * Female * Mixed * sexual minority * homeowner/part owner	-3.16 (-9.76, 3.45)	0.349	-2.91 (-8.19, 2.37)	0.280	-2.78 (-9.05, 3.50)	0.386	-1.21 (-6.05, 3.64)	0.626
MCS * Female * South Asian * sexual minority * homeowner/part owner	-0.75 (-5.86, 4.36)	0.774	-1.29 (-6.17, 3.58)	0.603	-2.64 (-7.41, 2.14)	0.279	-5.94 (-10.26, -1.62)	0.007
**Intercept**	1.42 (1.26, 1.59)	<0.001	1.36 (1.04, 1.68)	<0.001	1.54 (1.38, 1.70)	<0.001	1.52 (1.23, 1.80)	<0.001

	**UCLA-3**				**ONS life satisfaction**			
	**Unweighted**		**Weighted**		**Unweighted**		**Weighted**	
	**Coefficient (95% CI)**	***p***	**Coefficient (95% CI)**	***p***	**Coefficient (95% CI)**	***p***	**Coefficient (95% CI)**	***p***
**Cohort (MCS)**	0.13 (-0.08, 0.35)	0.233	0.08 (-0.29, 0.46)	0.661	-0.17 (-0.43, 0.09)	0.207	-0.14 (-0.68, 0.40)	0.612
**Birth sex (female)**	0.27 (0.06, 0.49)	0.011	0.32 (-0.04, 0.68)	0.084	-0.13 (-0.39, 0.13)	0.329	0.01 (-0.49, 0.50)	0.973
**Ethnic group**								
Mixed	0.50 (-0.20, 1.19)	0.161	0.54 (-0.17, 1.25)	0.139	-0.25 (-1.11, 0.60)	0.564	-1.04 (-2.44, 0.36)	0.145
South Asian	-0.06 (-0.48, 0.36)	0.780	0.14 (-0.48, 0.75)	0.667	-0.08 (-0.59, 0.43)	0.759	-0.03 (-0.87, 0.82)	0.952
Black	0.01 (-0.70, 0.72)	0.972	0.74 (-0.53, 2.01)	0.253	0.15 (-0.71, 1.00)	0.735	-1.40 (-3.60, 0.80)	0.212
Other	-0.57 (-1.56, 0.42)	0.257	-0.42 (-1.47, 0.63)	0.432	-0.29 (-1.50, 0.93)	0.646	-0.74 (-2.67, 1.19)	0.452
**Sexual orientation (sexual minority)**	0.85 (0.34, 1.36)	0.001	1.20 (0.50, 1.90)	0.001	-1.25 (-1.88, -0.62)	<0.001	-1.63 (-2.88, -0.39)	0.010
**Housing tenure (homeowner/part owner)**	-0.63 (-0.85, -0.41)	<0.001	-0.60 (-0.97, -0.23)	0.002	0.57 (0.30, 0.84)	<0.001	0.68 (0.19, 1.17)	0.007
**Cohort * birth sex**								
MCS * female	0.03 (-0.24, 0.31)	0.811	-0.06 (-0.54, 0.43)	0.814	-0.28 (-0.63, 0.06)	0.103	-0.40 (-1.09, 0.29)	0.258
**Cohort * ethnic group**								
MCS * Mixed	-0.64 (-1.60, 0.32)	0.190	-0.02 (-1.55, 1.50)	0.977	-0.22 (-1.40, 0.97)	0.722	-0.77 (-3.39, 1.85)	0.565
MCS * South Asian	-0.19 (-0.83, 0.44)	0.550	-0.39 (-1.28, 0.50)	0.390	-0.15 (-0.93, 0.63)	0.711	-0.33 (-1.44, 0.79)	0.565
MCS * Black	-0.38 (-1.35, 0.59)	0.442	-0.91 (-2.61, 0.79)	0.292	-0.84 (-2.02, 0.35)	0.166	-0.69 (-3.46, 2.09)	0.628
MCS * Other	1.84 (0.38, 3.30)	0.014	2.11 (0.84, 3.38)	0.001	-1.00 (-2.75, 0.76)	0.266	-1.83 (-4.12, 0.45)	0.116
**Birth sex * ethnic group**								
Female * Mixed	-0.52 (-1.34, 0.30)	0.217	-0.29 (-1.52, 0.93)	0.637	0.34 (-0.68, 1.35)	0.516	0.68 (-1.18, 2.54)	0.474
Female * South Asian	-0.10 (-0.62, 0.42)	0.708	-0.14 (-0.99, 0.71)	0.746	0.10 (-0.53, 0.74)	0.751	0.29 (-0.69, 1.26)	0.560
Female * Black	-0.28 (-1.14, 0.57)	0.515	-1.04 (-2.53, 0.45)	0.171	-0.45 (-1.49, 0.59)	0.395	0.39 (-1.93, 2.72)	0.740
Female * Other	0.77 (-0.37, 1.91)	0.185	0.96 (-0.39, 2.31)	0.165	0.02 (-1.39, 1.42)	0.980	0.25 (-1.89, 2.39)	0.816
**Cohort * birth sex * ethnic group**								
MCS * Female * Mixed	0.77 (-0.39, 1.92)	0.193	-0.19 (-2.09, 1.71)	0.846	-0.13 (-1.55, 1.30)	0.859	0.39 (-2.59, 3.37)	0.800
MCS * Female * South Asian	0.49 (-0.32, 1.31)	0.233	0.79 (-0.44, 2.02)	0.210	-0.11 (-1.11, 0.89)	0.827	-0.40 (-1.77, 0.96)	0.564
MCS * Female * Black	0.74 (-0.46, 1.94)	0.227	1.14 (-0.91, 3.18)	0.275	1.32 (-0.15, 2.79)	0.079	2.11 (-0.83, 5.04)	0.159
MCS * Female * Other	-1.95 (-3.63, -0.26)	0.024	-2.66 (-4.36, -0.96)	0.002	0.80 (-1.25, 2.84)	0.445	2.22 (-0.36, 4.79)	0.091
**Cohort * sexual orientation**								
MCS * sexual minority	0.08 (-0.57, 0.72)	0.817	-0.28 (-1.40, 0.84)	0.625	0.59 (-0.21, 1.39)	0.148	0.39 (-1.04, 1.82)	0.590
**Birth sex * sexual orientation**								
Female * sexual minority	-0.18 (-0.85, 0.49)	0.596	-0.31 (-1.33, 0.72)	0.557	0.38 (-0.45, 1.21)	0.367	0.90 (-0.81, 2.61)	0.301
**Cohort * birth sex * sexual orientation**								
MCS * Female * sexual minority	-0.01 (-0.83, 0.81)	0.977	0.43 (-0.99, 1.84)	0.553	-0.55 (-1.56, 0.46)	0.288	-0.61 (-2.53, 1.31)	0.533
**Ethnic group * sexual orientation**								

Mixed * sexual minority	-0.25 (-3.73, 3.23)	0.887	-0.61 (-1.56, 0.34)	0.207	1.05 (-3.24, 5.34)	0.632	2.25 (0.43, 4.08)	0.016
South Asian * sexual minority	-0.20 (-1.71, 1.32)	0.801	0.51 (-2.06, 3.08)	0.696	0.21 (-1.66, 2.08)	0.826	-0.20 (-2.97, 2.56)	0.885
Black * sexual minority	-2.88 (-8.12, 2.35)	0.280	-1.26 (-3.61, 1.10)	0.295	-1.35 (-5.64, 2.94)	0.537	0.61 (-1.89, 3.11)	0.631
Other * sexual minority	0.82 (-1.20, 2.83)	0.427	0.72 (-3.10, 4.55)	0.712	0.33 (-2.15, 2.82)	0.794	-0.55 (-5.44, 4.34)	0.825
**Cohort * ethnic group * sexual orientation**								
MCS * Mixed * sexual minority	-1.23 (-5.29, 2.82)	0.552	-1.07 (-3.07, 0.92)	0.292	0.79 (-4.21, 5.80)	0.756	1.89 (-1.54, 5.33)	0.280
MCS * South Asian * sexual minority	-0.01 (-2.90, 2.88)	0.992	-0.77 (-4.12, 2.58)	0.651	-2.61 (-6.17, 0.96)	0.152	-1.22 (-6.14, 3.71)	0.628
MCS * Black * sexual minority	-1.59 (-4.63, 1.45)	0.306	-1.43 (-3.06, 0.21)	0.087	6.42 (0.37, 12.47)	0.038	6.43 (3.35, 9.52)	<0.001
MCS * Other * sexual minority	-0.72 (-5.84, 4.39)	0.781	-3.47 (-8.27, 1.33)	0.157	2.13 (-4.17, 8.44)	0.507	1.63 (-3.78, 7.05)	0.555
**Birth sex * ethnic group * sexual orientation**								
Female * Mixed * sexual minority	-0.08 (-3.99, 3.84)	0.970	-0.27 (-2.46, 1.91)	0.807	-1.34 (-6.16, 3.49)	0.587	-2.17 (-5.79, 1.44)	0.238
Female * South Asian * sexual minority	0.84 (-1.68, 3.36)	0.514	0.21 (-2.63, 3.06)	0.883	-0.69 (-3.56, 2.18)	0.639	-1.03 (-6.34, 4.27)	0.703
Female * Black * sexual minority	2.31 (-2.61, 7.22)	0.358	0.50 (-1.41, 2.41)	0.609	2.70 (-2.13, 7.53)	0.273	1.40 (-2.46, 5.26)	0.477
Female * Other * sexual minority	-0.86 (-3.58, 1.85)	0.533	-1.50 (-5.58, 2.57)	0.470	0.23 (-3.12, 3.59)	0.892	1.44 (-3.88, 6.76)	0.596
**Cohort * birth sex * ethnic group * sexual orientation**								
MCS * Female * Mixed * sexual minority	1.38 (-3.20, 5.96)	0.555	2.11 (-1.44, 5.66)	0.244	0.08 (-5.57, 5.73)	0.979	-1.66 (-7.49, 4.17)	0.576
MCS * Female * South Asian * sexual minority	-0.35 (-4.10, 3.40)	0.855	-0.76 (-4.53, 3.02)	0.694	3.40 (-1.06, 7.87)	0.135	4.17 (-2.82, 11.16)	0.242
MCS * Female * Black * sexual minority					-8.98 (-16.11, -1.86)	0.013	-9.87 (-14.18, -5.55)	<0.001
MCS * Female * Other * sexual minority	-0.19 (-5.37, 4.98)	0.941	3.09 (-1.67, 7.85)	0.203	-0.87 (-7.25, 5.51)	0.789	-0.59 (-6.35, 5.17)	0.840
**Cohort * housing tenure**								
MCS * homeowner/part owner	0.74 (0.44, 1.04)	<0.001	0.87 (0.36, 1.37)	0.001	-0.78 (-1.15, -0.41)	<0.001	-1.09 (-1.79, -0.38)	0.003
**Birth sex * housing tenure**								
Female * homeowner/part owner	0.08 (-0.20, 0.36)	0.581	0.10 (-0.34, 0.54)	0.661	-0.10 (-0.44, 0.24)	0.569	-0.39 (-0.99, 0.21)	0.201
**Cohort * birth sex * housing tenure**								
MCS * female * homeowner/part owner	-0.31 (-0.70, 0.08)	0.122	-0.32 (-0.97, 0.33)	0.331	0.50 (0.02, 0.98)	0.043	0.99 (0.10, 1.89)	0.029
**Ethnic group * housing tenure**								
Mixed * homeowner/part owner	-0.25 (-1.25, 0.75)	0.623	-0.52 (-1.48, 0.44)	0.284	-0.68 (-1.91, 0.55)	0.279	0.63 (-1.21, 2.47)	0.501
South Asian * homeowner/part owner	0.28 (-0.25, 0.81)	0.301	0.14 (-0.85, 1.14)	0.776	0.07 (-0.58, 0.72)	0.837	0.08 (-0.97, 1.12)	0.885
Black * homeowner/part owner	0.18 (-0.87, 1.24)	0.732	-0.82 (-2.44, 0.79)	0.319	0.13 (-1.14, 1.41)	0.839	1.30 (-1.40, 3.99)	0.346
Other * homeowner/part owner	1.75 (0.32, 3.17)	0.016	3.10 (0.62, 5.58)	0.014	-1.10 (-2.86, 0.66)	0.221	-1.57 (-3.68, 0.54)	0.145
**Cohort * ethnic group * housing tenure**								
MCS * Mixed * homeowner/part owner	0.09 (-1.28, 1.47)	0.894	0.23 (-1.71, 2.16)	0.820	1.50 (-0.20, 3.20)	0.084	1.51 (-1.43, 4.45)	0.315
MCS * South Asian * homeowner/part owner	-0.62 (-1.46, 0.22)	0.147	-0.58 (-1.77, 0.61)	0.337	0.38 (-0.66, 1.41)	0.474	0.58 (-1.03, 2.19)	0.481
MCS * Black * homeowner/part owner	-0.30 (-1.89, 1.29)	0.712	0.70 (-1.43, 2.83)	0.520	0.99 (-0.96, 2.93)	0.320	1.07 (-2.41, 4.56)	0.546
MCS * Other * homeowner/part owner	-3.06 (-5.15, -0.98)	0.004	-3.84 (-7.06, -0.61)	0.020	2.49 (-0.02, 5.00)	0.052	4.65 (1.90, 7.40)	0.001
**Birth sex * ethnic group * housing tenure**								
Female * Mixed * homeowner/part owner	0.45 (-0.76, 1.65)	0.466	0.14 (-1.34, 1.61)	0.857	0.49 (-1.00, 1.97)	0.520	-0.20 (-2.49, 2.08)	0.862
Female * South Asian * homeowner/part owner	0.00 (-0.67, 0.67)	0.993	-0.21 (-1.40, 0.97)	0.726	-0.36 (-1.18, 0.47)	0.397	-0.45 (-1.69, 0.79)	0.477
Female * Black * homeowner/part owner	0.03 (-1.25, 1.31)	0.961	1.79 (-0.54, 4.12)	0.133	-0.04 (-1.60, 1.52)	0.956	-0.71 (-3.74, 2.32)	0.645
Female * Other * homeowner/part owner	-1.62 (-3.32, 0.07)	0.061	-4.09 (-6.99, -1.19)	0.006	0.94 (-1.16, 3.04)	0.379	2.34 (-0.18, 4.87)	0.069
**Cohort * birth sex * ethnic group * housing tenure**								
MCS * Female * Mixed * homeowner/part owner	0.09 (-1.68, 1.86)	0.918	1.24 (-1.44, 3.92)	0.366	-1.53 (-3.71, 0.65)	0.170	-2.56 (-6.02, 0.91)	0.148
MCS * Female * South Asian * homeowner/part owner	0.42 (-0.67, 1.51)	0.451	1.62 (-0.68, 3.93)	0.167	-0.17 (-1.51, 1.17)	0.799	-1.00 (-3.34, 1.34)	0.401
MCS * Female * Black * homeowner/part owner	0.52 (-1.97, 3.00)	0.684	-1.56 (-4.94, 1.82)	0.366	-1.06 (-4.11, 2.00)	0.498	-1.25 (-5.38, 2.88)	0.553
MCS * Female * Other * homeowner/part owner	3.09 (0.56, 5.61)	0.017	5.24 (1.56, 8.92)	0.005	-3.79 (-6.85, -0.72)	0.015	-7.95 (-11.30, -4.60)	<0.001
**Sexual orientation * housing tenure**								
Sexual minority * homeowner/part owner	0.18 (-0.56, 0.92)	0.631	-1.13 (-2.11, -0.14)	0.026	0.32 (-0.59, 1.24)	0.488	1.55 (-0.21, 3.31)	0.084
**Cohort * sexual orientation * housing tenure**								
MCS * sexual minority * homeowner/part owner	-0.40 (-1.43, 0.63)	0.444	0.65 (-0.97, 2.27)	0.432	-0.51 (-1.78, 0.75)	0.425	-0.74 (-2.98, 1.51)	0.518
**Birth sex * sexual orientation * housing tenure**								

Female * sexual minority * homeowner/part owner	-0.23 (-1.22, 0.77)	0.657	0.64 (-0.73, 2.01)	0.358	-0.12 (-1.35, 1.11)	0.851	-1.46 (-3.70, 0.79)	0.204
**Cohort * birth sex * sexual orientation * housing tenure**								
MCS * Female * sexual minority * homeowner/part owner	0.61 (-0.69, 1.92)	0.358	-0.97 (-3.28, 1.34)	0.411	0.47 (-1.13, 2.08)	0.562	1.20 (-1.54, 3.94)	0.391
**Ethnic group * sexual orientation * housing tenure**								
Mixed * sexual minority * homeowner/part owner	2.70 (-2.23, 7.62)	0.283	4.25 (2.90, 5.59)	<0.001	-0.21 (-6.29, 5.87)	0.946	-2.86 (-5.35, -0.37)	0.024
South Asian * sexual minority * homeowner/part owner	0.29 (-1.73, 2.32)	0.778	0.29 (-2.68, 3.26)	0.849	-1.04 (-3.54, 1.46)	0.413	-3.47 (-7.23, 0.28)	0.070
Black * sexual minority * homeowner/part owner	2.38 (-1.52, 6.27)	0.231	1.99 (-0.01, 3.98)	0.051	-0.02 (-6.11, 6.06)	0.994	-2.53 (-5.70, 0.65)	0.119
Other * sexual minority * homeowner/part owner	-1.80 (-5.13, 1.52)	0.288	-1.92 (-6.42, 2.58)	0.402	-0.54 (-4.65, 3.56)	0.795	1.33 (-3.91, 6.57)	0.620
**Cohort * ethnic group * sexual orientation * housing tenure**								
MCS * Mixed * sexual minority * homeowner/part owner	-1.10 (-6.86, 4.66)	0.708	-2.52 (-5.79, 0.75)	0.130	-2.21 (-9.32, 4.90)	0.543	-2.54 (-7.26, 2.18)	0.292
MCS * South Asian * sexual minority * homeowner/part owner	0.32 (-3.48, 4.13)	0.868	1.02 (-2.79, 4.83)	0.600	1.33 (-3.36, 6.03)	0.577	3.20 (-2.39, 8.79)	0.261
MCS * Black * sexual minority * homeowner/part owner								
MCS * Other * sexual minority * homeowner/part owner	0.40 (-4.70, 5.51)	0.877	1.18 (-3.73, 6.09)	0.636	0.24 (-6.05, 6.54)	0.939	-1.31 (-6.82, 4.20)	0.640
**Birth sex * ethnic group * sexual orientation * housing tenure**								
Female * Mixed * sexual minority * homeowner/part owner	-2.55 (-8.10, 3.00)	0.368	-3.07 (-5.57, -0.56)	0.016	-0.77 (-7.62, 6.08)	0.826	1.38 (-3.27, 6.03)	0.561
Female * South Asian * sexual minority * homeowner/part owner	-0.64 (-4.14, 2.86)	0.720	0.05 (-3.27, 3.37)	0.975	0.66 (-3.49, 4.81)	0.756	3.96 (-2.92, 10.83)	0.259
Female * Black * sexual minority * homeowner/part owner					-1.24 (-8.99, 6.52)	0.754	0.86 (-3.72, 5.43)	0.714
Female * Other * sexual minority * homeowner/part owner								
**Cohort * birth sex * ethnic group * sexual orientation * housing tenure**								
MCS * Female * Mixed * sexual minority * homeowner/part owner	1.63 (-4.92, 8.19)	0.626	2.36 (-2.74, 7.46)	0.364	1.92 (-6.17, 10.01)	0.643	3.02 (-4.49, 10.53)	0.431
MCS * Female * South Asian * sexual minority * homeowner/part owner	-0.93 (-6.01, 4.15)	0.719	-2.12 (-7.04, 2.81)	0.399	-1.01 (-7.16, 5.14)	0.748	-5.13 (-13.70, 3.44)	0.240
**Intercept**	4.90 (4.74, 5.07)	<0.001	4.88 (4.57, 5.18)	<0.001	6.45 (6.25, 6.66)	<0.001	6.42 (6.00, 6.83)	<0.001

Note. CI: confidence interval; GAD-2: 2-item Generalised Anxiety Disorder questionnaire; MCS: Millennium Cohort Study (born in 2000-2002); NS: Next Steps study (born in 1990); ONS: Office for National Statistics; PHQ-2: 2-item depression Patient Health Questionnaire; UCLA-3: 3-item University of California Los Angeles loneliness scale.

Weighted results are adjusted for survey design characteristics, including survey and non-response weights. White includes all White groups; South Asian includes Bangladeshi, Indian, and Pakistani groups; Black includes Black African, Black Caribbean, and Black British groups; Other includes all other ethnic group not included in the other categories.

Table S9. Results from the fixed-effects multiple regression approach using stratification 80c (cohort * birth sex * ethnic group * sexual orientation * childhood social class).

	GAD-2				PHQ-2			
	Unweighted		Weighted		Unweighted		Weighted	
	Coefficient (95% CI)	*p*	Coefficient (95% CI)	*p*	Coefficient (95% CI)	*p*	Coefficient (95% CI)	*p*
**Cohort (MCS)**	0.33 (0.01, 0.65)	0.043	0.30 (-0.14, 0.74)	0.177	0.36 (0.06, 0.67)	0.019	0.32 (-0.18, 0.81)	0.211
**Birth sex (female)**	0.88 (0.64, 1.13)	<0.001	1.02 (0.58, 1.46)	<0.001	0.51 (0.27, 0.74)	<0.001	0.79 (0.42, 1.17)	<0.001
**Ethnic group**								
Mixed	-0.09 (-0.96, 0.78)	0.837	-0.65 (-1.23, -0.07)	0.029	0.07 (-0.76, 0.90)	0.864	0.14 (-0.45, 0.73)	0.638
South Asian	-0.05 (-0.42, 0.32)	0.799	-0.03 (-0.58, 0.52)	0.915	-0.17 (-0.52, 0.18)	0.343	-0.28 (-0.71, 0.14)	0.194
Black	-0.59 (-1.59, 0.41)	0.246	-0.28 (-1.11, 0.56)	0.519	-0.57 (-1.52, 0.38)	0.237	0.09 (-1.24, 1.41)	0.900
Other	-0.45 (-1.50, 0.59)	0.392	0.11 (-1.59, 1.81)	0.900	-0.24 (-1.23, 0.75)	0.634	0.40 (-1.58, 2.38)	0.694
**Sexual orientation (sexual minority)**	0.62 (-0.05, 1.29)	0.068	-0.32 (-1.03, 0.38)	0.372	0.62 (-0.02, 1.25)	0.058	0.49 (-0.62, 1.61)	0.386
**Childhood social class (advantaged)**	0.17 (-0.06, 0.41)	0.147	0.11 (-0.28, 0.50)	0.573	0.05 (-0.17, 0.27)	0.652	0.12 (-0.23, 0.47)	0.506
**Cohort * birth sex**								
MCS * female	0.34 (-0.07, 0.75)	0.103	0.21 (-0.44, 0.85)	0.527	0.26 (-0.13, 0.65)	0.193	-0.09 (-0.74, 0.57)	0.798
**Cohort * ethnic group**								
MCS * Mixed	0.00 (-1.65, 1.66)	0.997	0.46 (-0.84, 1.75)	0.490	-0.01 (-1.58, 1.57)	0.991	-0.33 (-1.64, 0.98)	0.624
MCS * South Asian	-0.05 (-0.75, 0.65)	0.892	-0.23 (-1.01, 0.56)	0.569	-0.06 (-0.72, 0.61)	0.865	0.21 (-1.00, 1.41)	0.737
MCS * Black	1.29 (-0.29, 2.88)	0.109	0.97 (-0.36, 2.31)	0.153	0.47 (-1.03, 1.97)	0.540	0.67 (-1.44, 2.77)	0.533
MCS * Other	-0.25 (-1.92, 1.42)	0.767	-0.73 (-2.66, 1.19)	0.455	-0.65 (-2.24, 0.94)	0.424	-1.15 (-3.34, 1.04)	0.302
**Birth sex * ethnic group**								
Female * Mixed	-0.06 (-1.12, 1.00)	0.910	0.74 (-0.83, 2.30)	0.357	0.12 (-0.89, 1.13)	0.814	0.09 (-1.47, 1.64)	0.910
Female * South Asian	-0.24 (-0.72, 0.24)	0.334	-0.20 (-0.92, 0.53)	0.600	-0.03 (-0.49, 0.43)	0.889	-0.26 (-0.81, 0.30)	0.365
Female * Black	-0.28 (-1.47, 0.90)	0.638	0.25 (-1.45, 1.95)	0.772	-0.14 (-1.27, 0.99)	0.806	-0.64 (-2.34, 1.07)	0.463
Female * Other	-0.06 (-1.32, 1.20)	0.922	-0.54 (-2.48, 1.41)	0.588	-0.22 (-1.41, 0.98)	0.723	-0.84 (-3.01, 1.33)	0.450
**Cohort * birth sex * ethnic group**								
MCS * Female * Mixed	-0.02 (-1.94, 1.90)	0.982	-0.14 (-2.26, 1.98)	0.898	-0.84 (-2.67, 0.99)	0.368	-0.01 (-2.07, 2.05)	0.992
MCS * Female * South Asian	0.03 (-0.88, 0.93)	0.955	-0.05 (-1.18, 1.08)	0.929	0.16 (-0.69, 1.02)	0.707	0.23 (-1.10, 1.57)	0.734
MCS * Female * Black	-0.97 (-2.99, 1.06)	0.350	-1.12 (-3.36, 1.13)	0.330	-0.23 (-2.15, 1.70)	0.818	-0.06 (-2.50, 2.37)	0.958
MCS * Female * Other	0.36 (-1.65, 2.36)	0.726	1.19 (-1.55, 3.92)	0.394	1.38 (-0.52, 3.29)	0.155	2.37 (-0.24, 4.97)	0.075
**Cohort * sexual orientation**								
MCS * sexual minority	0.60 (-0.49, 1.69)	0.279	1.60 (0.26, 2.94)	0.020	0.25 (-0.79, 1.29)	0.636	-0.21 (-1.63, 1.21)	0.773
**Birth sex * sexual orientation**								
Female * sexual minority	-0.13 (-0.99, 0.72)	0.761	0.04 (-1.42, 1.50)	0.960	-0.05 (-0.87, 0.76)	0.896	-0.39 (-1.90, 1.12)	0.612
**Cohort * birth sex * sexual orientation**								
MCS * Female * sexual minority	-0.07 (-1.39, 1.24)	0.913	-1.32 (-3.77, 1.13)	0.290	0.45 (-0.80, 1.70)	0.484	0.54 (-1.80, 2.89)	0.650
**Ethnic group * sexual orientation**								
Mixed * sexual minority	-0.19 (-6.77, 6.40)	0.956	-1.58 (-6.47, 3.31)	0.527	-5.25 (-11.51, 1.01)	0.100	-3.40 (-7.65, 0.84)	0.116
South Asian * sexual minority	0.00 (-1.35, 1.35)	0.999	1.44 (0.27, 2.60)	0.016	-0.35 (-1.64, 0.93)	0.590	0.59 (-0.66, 1.84)	0.353
Black * sexual minority	-0.12 (-3.72, 3.47)	0.947	0.56 (-0.48, 1.61)	0.290	2.72 (-0.70, 6.14)	0.119	2.27 (0.56, 3.98)	0.009
Other * sexual minority	3.74 (1.05, 6.43)	0.007	4.96 (3.08, 6.85)	<0.001	3.38 (0.82, 5.94)	0.010	3.74 (1.43, 6.06)	0.002
**Cohort * ethnic group * sexual orientation**								
MCS * Mixed * sexual minority	1.63 (-3.77, 7.03)	0.555	3.16 (-1.44, 7.76)	0.178	3.72 (-1.42, 8.85)	0.156	2.84 (-1.10, 6.78)	0.157
MCS * South Asian * sexual minority	0.45 (-2.49, 3.38)	0.764	-0.79 (-2.49, 0.91)	0.362	1.11 (-1.68, 3.90)	0.435	1.94 (-0.30, 4.18)	0.090
MCS * Black * sexual minority	-1.23 (-6.39, 3.93)	0.641	-1.87 (-3.71, -0.03)	0.046	-5.08 (-9.99, -0.18)	0.042	-4.77 (-7.26, -2.29)	<0.001
MCS * Other * sexual minority	-7.00 (-13.97, -0.03)	0.049	-6.75 (-11.19, -2.31)	0.003	-11.17 (-17.80, -4.54)	0.001	-7.64 (-12.07, -3.21)	0.001
**Birth sex * ethnic group * sexual orientation**								
Female * Mixed * sexual minority	-1.13 (-6.72, 4.46)	0.692	0.72 (-3.77, 5.22)	0.753	2.75 (-2.56, 8.06)	0.310	1.13 (-2.75, 5.00)	0.568

Female * South Asian * sexual minority	-2.18 (-5.88, 1.52)	0.248	-2.98 (-4.64, -1.32)	<0.001	-1.75 (-5.27, 1.77)	0.329	-2.10 (-3.67, -0.53)	0.009
Female * Black * sexual minority	3.53 (-1.48, 8.54)	0.167	2.69 (0.49, 4.89)	0.017	-3.31 (-8.08, 1.45)	0.173	-2.77 (-5.02, -0.52)	0.016
Female * Other * sexual minority	-3.69 (-8.11, 0.73)	0.102	-4.30 (-6.60, -2.01)	<0.001	-1.24 (-5.44, 2.97)	0.564	-1.35 (-3.93, 1.23)	0.305
**Cohort * birth sex * ethnic group * sexual orientation**								
MCS * Female * Mixed * sexual minority	-0.47 (-3.99, 3.05)	0.792	-1.46 (-4.25, 1.33)	0.306	0.81 (-2.53, 4.16)	0.635	1.97 (-0.95, 4.90)	0.185
MCS * Female * South Asian * sexual minority	0.17 (-4.65, 4.99)	0.944	2.08 (-0.53, 4.69)	0.119	-0.34 (-4.92, 4.25)	0.886	-0.26 (-3.39, 2.87)	0.872
MCS * Female * Black * sexual minority	3.40 (-3.30, 10.10)	0.320	4.16 (-0.69, 9.02)	0.093	5.54 (-0.83, 11.91)	0.088	6.03 (0.83, 11.24)	0.023
MCS * Female * Other * sexual minority	6.69 (1.25, 12.14)	0.016	6.85 (3.58, 10.11)	<0.001	6.12 (0.94, 11.30)	0.021	3.20 (-0.43, 6.82)	0.084
**Cohort * childhood social class**								
MCS * advantaged	-0.10 (-0.46, 0.26)	0.588	0.01 (-0.50, 0.52)	0.965	-0.09 (-0.43, 0.26)	0.616	-0.03 (-0.58, 0.53)	0.919
**Birth sex * childhood social class**								
Female * advantaged	-0.28 (-0.58, 0.01)	0.062	-0.39 (-0.89, 0.11)	0.129	-0.30 (-0.59, -0.02)	0.034	-0.61 (-1.05, -0.17)	0.007
**Cohort * birth sex * childhood social class**								
MCS * female * advantaged	-0.06 (-0.53, 0.41)	0.801	-0.01 (-0.74, 0.72)	0.979	-0.06 (-0.50, 0.38)	0.789	0.27 (-0.46, 1.00)	0.468
**Ethnic group * childhood social class**								
Mixed * advantaged	0.05 (-1.02, 1.12)	0.922	0.58 (-0.42, 1.59)	0.252	-0.02 (-1.03, 0.99)	0.970	-0.16 (-1.24, 0.92)	0.773
South Asian * advantaged	0.17 (-0.38, 0.73)	0.545	0.29 (-0.49, 1.07)	0.461	0.12 (-0.41, 0.65)	0.657	0.14 (-0.55, 0.83)	0.691
Black * advantaged	0.03 (-1.17, 1.23)	0.959	0.21 (-1.08, 1.51)	0.746	0.50 (-0.64, 1.65)	0.386	-0.02 (-1.52, 1.48)	0.980
Other * advantaged	0.44 (-1.15, 2.04)	0.587	0.95 (-1.42, 3.33)	0.433	0.32 (-1.19, 1.84)	0.675	0.99 (-1.97, 3.96)	0.512
**Cohort * ethnic group * childhood social class**								
MCS * Mixed * advantaged	-0.01 (-1.86, 1.83)	0.988	-0.10 (-1.75, 1.54)	0.900	0.17 (-1.58, 1.92)	0.851	1.64 (-0.25, 3.53)	0.088
MCS * South Asian * advantaged	-0.55 (-1.47, 0.36)	0.237	-0.50 (-1.55, 0.54)	0.344	-0.17 (-1.04, 0.70)	0.707	-0.17 (-1.67, 1.33)	0.825
MCS * Black * advantaged	-0.84 (-2.74, 1.06)	0.386	-1.30 (-3.15, 0.54)	0.165	-0.58 (-2.38, 1.23)	0.530	-1.34 (-3.70, 1.02)	0.265
MCS * Other * advantaged	0.24 (-2.00, 2.48)	0.834	0.19 (-2.59, 2.97)	0.892	0.67 (-1.47, 2.80)	0.541	-0.64 (-3.79, 2.50)	0.687
**Birth sex * ethnic group * childhood social class**								
Female * Mixed * advantaged	0.06 (-1.24, 1.36)	0.929	-0.68 (-2.51, 1.16)	0.470	-0.15 (-1.38, 1.08)	0.816	-0.11 (-1.98, 1.76)	0.909
Female * South Asian * advantaged	0.03 (-0.71, 0.76)	0.939	-0.42 (-1.44, 0.61)	0.424	-0.01 (-0.71, 0.69)	0.981	0.23 (-0.61, 1.08)	0.587
Female * Black * advantaged	0.16 (-1.27, 1.60)	0.822	-0.35 (-2.51, 1.81)	0.752	-0.13 (-1.49, 1.23)	0.850	0.80 (-1.34, 2.94)	0.462
Female * Other * advantaged	0.00 (-1.85, 1.86)	0.997	-0.06 (-2.76, 2.64)	0.963	0.53 (-1.23, 2.30)	0.553	-0.04 (-3.18, 3.09)	0.978
**Cohort * birth sex * ethnic group * childhood social class**								
MCS * Female * Mixed * advantaged	-0.18 (-2.36, 2.01)	0.874	-0.08 (-2.59, 2.44)	0.951	0.64 (-1.43, 2.72)	0.543	-1.31 (-3.96, 1.33)	0.330
MCS * Female * South Asian * advantaged	0.39 (-0.81, 1.60)	0.521	0.93 (-0.57, 2.43)	0.223	0.21 (-0.94, 1.36)	0.721	-0.26 (-1.93, 1.41)	0.763
MCS * Female * Black * advantaged	1.09 (-1.35, 3.52)	0.382	1.93 (-0.91, 4.76)	0.183	0.71 (-1.61, 3.03)	0.549	0.34 (-2.41, 3.10)	0.808
MCS * Female * Other * advantaged	-0.56 (-3.24, 2.13)	0.684	-0.18 (-4.04, 3.67)	0.925	-1.36 (-3.91, 1.19)	0.296	-0.33 (-4.03, 3.37)	0.861
**Sexual orientation * childhood social class**								
Sexual minority * advantaged	-0.20 (-1.01, 0.60)	0.620	0.48 (-0.51, 1.47)	0.345	-0.29 (-1.05, 0.48)	0.460	-0.10 (-1.43, 1.23)	0.882
**Cohort * sexual orientation * childhood social class**								
MCS * sexual minority * advantaged	-0.09 (-1.33, 1.15)	0.890	-0.93 (-2.62, 0.75)	0.278	0.54 (-0.64, 1.72)	0.371	0.68 (-1.03, 2.39)	0.435
**Birth sex * sexual orientation * childhood social class**								
Female * sexual minority * advantaged	0.50 (-0.55, 1.55)	0.352	0.48 (-1.26, 2.23)	0.587	0.61 (-0.39, 1.61)	0.233	0.76 (-1.04, 2.56)	0.410
**Cohort * birth sex * sexual orientation * childhood social class**								
MCS * Female * sexual minority * advantaged	-0.70 (-2.22, 0.82)	0.368	0.65 (-2.13, 3.44)	0.645	-1.53 (-2.98, -0.09)	0.038	-1.07 (-3.72, 1.58)	0.428
**Ethnic group * sexual orientation * childhood social class**								
Mixed * sexual minority * advantaged	0.04 (-6.04, 6.12)	0.990	1.53 (-3.24, 6.29)	0.530	5.58 (-0.20, 11.37)	0.059	3.13 (-0.87, 7.13)	0.125
South Asian * sexual minority * advantaged	-1.14 (-3.59, 1.31)	0.363	-2.12 (-3.61, -0.64)	0.005	-0.55 (-2.88, 1.79)	0.645	-1.23 (-2.79, 0.32)	0.119
Black * sexual minority * advantaged	0.00 (-5.01, 5.01)	1.000	-0.80 (-2.39, 0.79)	0.322	-1.27 (-6.03, 3.50)	0.602	-1.00 (-2.97, 0.97)	0.320
Other * sexual minority * advantaged	-2.41 (-5.84, 1.02)	0.169	-2.93 (-5.72, -0.13)	0.040	-2.84 (-6.10, 0.43)	0.089	-4.30 (-8.01, -0.58)	0.023
**Cohort * ethnic group * sexual orientation * childhood social class**								
MCS * Mixed * sexual minority * advantaged	-1.26 (-5.75, 3.23)	0.582	-2.69 (-6.71, 1.32)	0.188	-4.34 (-8.61, -0.08)	0.046	-3.74 (-7.14, -0.33)	0.031

MCS * South Asian * sexual minority * advantaged	0.08 (-4.04, 4.20)	0.971	1.20 (-1.17, 3.57)	0.321	-1.95 (-5.87, 1.97)	0.329	-2.94 (-5.70, -0.19)	0.036
MCS * Black * sexual minority * advantaged								
MCS * Other * sexual minority * advantaged	3.27 (-2.36, 8.89)	0.255	1.92 (-1.76, 5.60)	0.305	7.84 (2.49, 13.19)	0.004	6.18 (2.94, 9.42)	<0.001
**Birth sex * ethnic group * sexual orientation * childhood social class**								
Female * Mixed * sexual minority * advantaged	1.09 (-3.69, 5.88)	0.655	-0.87 (-5.09, 3.34)	0.685	-3.62 (-8.17, 0.93)	0.118	-1.91 (-5.16, 1.33)	0.247
Female * South Asian * sexual minority * advantaged	0.75 (-4.70, 6.21)	0.786	1.56 (-0.46, 3.59)	0.130	1.37 (-3.81, 6.56)	0.604	1.70 (-0.25, 3.65)	0.088
Female * Black * sexual minority * advantaged	-2.38 (-8.97, 4.21)	0.478	-2.02 (-6.96, 2.91)	0.421	2.83 (-3.44, 9.09)	0.376	1.77 (-3.16, 6.71)	0.480
Female * Other * sexual minority * advantaged	0.28 (-5.23, 5.79)	0.921	-0.43 (-3.76, 2.90)	0.800	-1.59 (-6.83, 3.65)	0.553	-0.60 (-4.56, 3.36)	0.766
**Cohort * birth sex * ethnic group * sexual orientation * childhood social class**								
MCS * Female * South Asian * sexual minority * advantaged	1.23 (-5.47, 7.93)	0.719	-1.08 (-4.52, 2.35)	0.537	2.19 (-4.19, 8.56)	0.501	3.57 (-0.55, 7.69)	0.089
**Intercept**	1.09 (0.90, 1.28)	<0.001	1.03 (0.70, 1.36)	<0.001	1.24 (1.06, 1.42)	<0.001	1.15 (0.85, 1.45)	<0.001

	**UCLA-3**				**ONS life satisfaction**			
	**Unweighted**		**Weighted**		**Unweighted**		**Weighted**	
	**Coefficient (95% CI)**	***p***	**Coefficient (95% CI)**	***p***	**Coefficient (95% CI)**	***p***	**Coefficient (95% CI)**	***p***
**Cohort (MCS)**	0.57 (0.25, 0.88)	<0.001	0.53 (-0.04, 1.09)	0.069	-0.71 (-1.10, -0.33)	<0.001	-0.85 (-1.57, -0.14)	0.020
**Birth sex (female)**	0.47 (0.22, 0.71)	<0.001	0.40 (-0.01, 0.82)	0.055	-0.25 (-0.55, 0.05)	0.106	-0.63 (-1.07, -0.19)	0.006
**Ethnic group**								
Mixed	0.49 (-0.37, 1.35)	0.264	0.40 (-0.45, 1.25)	0.358	-0.83 (-1.89, 0.24)	0.128	-1.50 (-3.06, 0.07)	0.061
South Asian	0.06 (-0.31, 0.42)	0.757	-0.06 (-0.65, 0.54)	0.850	0.11 (-0.34, 0.56)	0.635	0.24 (-0.44, 0.93)	0.487
Black	-0.39 (-1.42, 0.64)	0.459	-0.43 (-1.47, 0.60)	0.410	1.07 (-0.15, 2.29)	0.085	0.81 (-0.34, 1.97)	0.169
Other	0.25 (-0.79, 1.28)	0.639	-0.21 (-1.32, 0.90)	0.708	-0.31 (-1.58, 0.96)	0.634	-0.53 (-2.32, 1.27)	0.564
**Sexual orientation (sexual minority)**	0.96 (0.30, 1.63)	0.004	0.67 (-0.71, 2.04)	0.344	-1.58 (-2.40, -0.77)	<0.001	-1.63 (-3.63, 0.37)	0.110
**Childhood social class (advantaged)**	-0.03 (-0.26, 0.20)	0.771	-0.18 (-0.55, 0.20)	0.362	0.04 (-0.25, 0.32)	0.791	-0.14 (-0.62, 0.34)	0.557
**Cohort * birth sex**								
MCS * female	0.02 (-0.38, 0.43)	0.905	0.00 (-0.72, 0.72)	0.991	-0.02 (-0.52, 0.48)	0.945	0.54 (-0.35, 1.43)	0.236
**Cohort * ethnic group**								
MCS * Mixed	0.20 (-1.44, 1.84)	0.808	0.03 (-1.13, 1.19)	0.956	0.61 (-1.41, 2.63)	0.555	1.13 (-0.66, 2.92)	0.214
MCS * South Asian	-0.67 (-1.37, 0.03)	0.062	-0.48 (-1.32, 0.35)	0.256	0.27 (-0.59, 1.12)	0.540	0.09 (-1.32, 1.49)	0.902
MCS * Black	0.00 (-1.59, 1.60)	0.999	-0.05 (-1.76, 1.67)	0.959	-1.25 (-3.17, 0.68)	0.206	-1.81 (-3.41, -0.21)	0.027
MCS * Other	-0.96 (-2.61, 0.70)	0.257	-0.76 (-2.42, 0.90)	0.372	1.26 (-0.78, 3.30)	0.226	1.64 (-1.39, 4.67)	0.288
**Birth sex * ethnic group**								
Female * Mixed	-0.83 (-1.87, 0.22)	0.123	-0.19 (-1.82, 1.45)	0.821	0.66 (-0.63, 1.95)	0.314	1.18 (-1.12, 3.48)	0.313
Female * South Asian	-0.16 (-0.63, 0.32)	0.524	-0.09 (-0.97, 0.79)	0.838	-0.19 (-0.78, 0.39)	0.519	0.15 (-0.67, 0.97)	0.718
Female * Black	-0.15 (-1.36, 1.06)	0.807	0.27 (-1.56, 2.10)	0.769	-0.82 (-2.26, 0.63)	0.267	-0.56 (-2.09, 0.96)	0.469
Female * Other	0.05 (-1.20, 1.30)	0.942	0.40 (-1.09, 1.89)	0.596	0.00 (-1.54, 1.54)	0.998	0.94 (-1.09, 2.98)	0.363
**Cohort * birth sex * ethnic group**								
MCS * Female * Mixed	0.17 (-1.74, 2.07)	0.865	-0.27 (-2.43, 1.90)	0.810	-0.85 (-3.20, 1.50)	0.477	-2.56 (-5.32, 0.20)	0.069
MCS * Female * South Asian	0.60 (-0.30, 1.51)	0.190	0.74 (-0.64, 2.12)	0.295	-0.16 (-1.25, 0.94)	0.780	-0.90 (-2.79, 0.98)	0.348
MCS * Female * Black	-0.19 (-2.22, 1.84)	0.852	-0.41 (-2.83, 2.00)	0.738	1.71 (-0.76, 4.18)	0.175	2.37 (0.20, 4.55)	0.032
MCS * Female * Other	0.21 (-1.78, 2.19)	0.838	0.07 (-2.27, 2.41)	0.956	-1.91 (-4.36, 0.53)	0.125	-2.85 (-6.16, 0.46)	0.092
**Cohort * sexual orientation**								
MCS * sexual minority	-0.16 (-1.24, 0.92)	0.769	-0.27 (-2.03, 1.49)	0.763	0.53 (-0.80, 1.87)	0.431	1.60 (-0.64, 3.84)	0.161
**Birth sex * sexual orientation**								
Female * sexual minority	-0.32 (-1.16, 0.53)	0.464	0.30 (-1.30, 1.89)	0.716	1.09 (0.05, 2.13)	0.040	2.14 (-0.06, 4.35)	0.057
**Cohort * birth sex * sexual orientation**								
MCS * Female * sexual minority	0.23 (-1.07, 1.53)	0.726	-0.84 (-3.30, 1.61)	0.501	-1.29 (-2.89, 0.31)	0.115	-3.22 (-5.82, -0.61)	0.016
**Ethnic group * sexual orientation**								

Mixed * sexual minority	-1.90 (-8.43, 4.62)	0.568	-3.35 (-7.51, 0.80)	0.114	7.66 (-0.37, 15.69)	0.062	3.49 (-1.36, 8.33)	0.158
South Asian * sexual minority	0.30 (-1.04, 1.64)	0.666	0.62 (-1.40, 2.64)	0.548	-0.18 (-1.83, 1.47)	0.834	-2.78 (-5.21, -0.35)	0.025
Black * sexual minority	0.71 (-4.76, 6.18)	0.799	-0.19 (-2.69, 2.32)	0.883	-2.25 (-6.64, 2.14)	0.315	-2.14 (-4.42, 0.15)	0.067
Other * sexual minority	3.22 (0.55, 5.89)	0.018	3.84 (2.10, 5.58)	<0.001	-1.87 (-5.16, 1.42)	0.265	-4.14 (-7.25, -1.04)	0.009
**Cohort * ethnic group * sexual orientation**								
MCS * Mixed * sexual minority	0.27 (-5.08, 5.62)	0.922	2.30 (-1.76, 6.36)	0.267	-4.44 (-11.03, 2.14)	0.186	-1.20 (-5.89, 3.49)	0.617
MCS * South Asian * sexual minority	0.88 (-2.04, 3.79)	0.555	0.50 (-1.86, 2.86)	0.679	-3.20 (-6.78, 0.38)	0.080	-0.42 (-3.89, 3.04)	0.810
MCS * Black * sexual minority	-1.84 (-6.07, 2.39)	0.395	-2.28 (-3.69, -0.86)	0.002	7.42 (1.13, 13.72)	0.021	7.06 (4.40, 9.72)	<0.001
MCS * Other * sexual minority	-5.63 (-12.55, 1.28)	0.110	-5.22 (-9.41, -1.04)	0.014	4.95 (-3.56, 13.46)	0.254	3.72 (-0.37, 7.80)	0.075
**Birth sex * ethnic group * sexual orientation**								
Female * Mixed * sexual minority	-0.45 (-5.99, 5.08)	0.873	0.07 (-3.75, 3.90)	0.970	-5.52 (-12.34, 1.29)	0.112	-2.01 (-6.48, 2.47)	0.379
Female * South Asian * sexual minority	1.32 (-4.21, 6.86)	0.640	2.29 (-1.61, 6.20)	0.249	-5.76 (-10.28, -1.24)	0.012	-4.45 (-7.08, -1.83)	0.001
Female * Black * sexual minority	0.14 (-4.10, 4.38)	0.948	0.28 (-1.51, 2.07)	0.760	3.98 (-2.14, 10.09)	0.203	3.05 (0.41, 5.70)	0.024
Female * Other * sexual minority	-1.20 (-5.58, 3.18)	0.591	-2.10 (-4.18, -0.03)	0.046	-0.85 (-6.24, 4.55)	0.759	-0.11 (-3.34, 3.13)	0.948
**Cohort * birth sex * ethnic group * sexual orientation**								
MCS * Female * Mixed * sexual minority	2.04 (-1.45, 5.52)	0.252	2.87 (0.25, 5.48)	0.032	2.17 (-2.12, 6.46)	0.321	-0.12 (-3.45, 3.22)	0.945
MCS * Female * South Asian * sexual minority	-2.68 (-7.29, 1.93)	0.255	-2.55 (-5.02, -0.07)	0.044	10.37 (4.49, 16.26)	0.001	8.10 (3.37, 12.83)	0.001
MCS * Female * Black * sexual minority					-10.95 (-19.12, -2.77)	0.009	-12.35 (-18.05, -6.65)	<0.001
MCS * Female * Other * sexual minority	1.72 (-3.68, 7.12)	0.532	2.83 (-0.48, 6.14)	0.093	0.19 (-6.45, 6.83)	0.956	2.09 (-1.45, 5.62)	0.247
**Cohort * childhood social class**								
MCS * advantaged	-0.03 (-0.39, 0.33)	0.872	-0.07 (-0.68, 0.54)	0.818	0.10 (-0.34, 0.55)	0.644	0.22 (-0.58, 1.02)	0.591
**Birth sex * childhood social class**								
Female * advantaged	-0.22 (-0.52, 0.07)	0.138	-0.12 (-0.59, 0.35)	0.611	0.06 (-0.30, 0.42)	0.747	0.51 (-0.08, 1.09)	0.090
**Cohort * birth sex * childhood social class**								
MCS * female * advantaged	-0.21 (-0.68, 0.25)	0.365	-0.27 (-1.03, 0.50)	0.499	0.06 (-0.50, 0.63)	0.827	-0.52 (-1.47, 0.42)	0.279
**Ethnic group * childhood social class**								
Mixed * advantaged	-0.09 (-1.15, 0.96)	0.866	-0.11 (-1.19, 0.97)	0.842	0.65 (-0.65, 1.95)	0.328	1.55 (-0.27, 3.36)	0.095
South Asian * advantaged	0.12 (-0.42, 0.67)	0.656	0.03 (-0.72, 0.78)	0.937	-0.41 (-1.08, 0.26)	0.232	-0.74 (-1.73, 0.25)	0.141
Black * advantaged	0.63 (-0.59, 1.86)	0.313	1.10 (-0.50, 2.71)	0.177	-1.28 (-2.74, 0.19)	0.087	-2.71 (-5.11, -0.31)	0.027
Other * advantaged	-0.03 (-1.61, 1.55)	0.966	0.76 (-0.73, 2.25)	0.315	-1.62 (-3.56, 0.33)	0.103	-2.13 (-4.24, -0.02)	0.048
**Cohort * ethnic group * childhood social class**								
MCS * Mixed * advantaged	-0.98 (-2.81, 0.84)	0.290	0.10 (-1.54, 1.74)	0.907	-0.54 (-2.78, 1.71)	0.638	-1.86 (-4.19, 0.47)	0.117
MCS * South Asian * advantaged	-0.03 (-0.95, 0.88)	0.948	0.24 (-0.85, 1.32)	0.669	0.43 (-0.68, 1.54)	0.452	0.42 (-1.31, 2.14)	0.634
MCS * Black * advantaged	-0.93 (-2.83, 0.98)	0.339	-1.34 (-3.57, 0.88)	0.235	1.76 (-0.55, 4.08)	0.136	3.94 (0.98, 6.89)	0.009
MCS * Other * advantaged	1.53 (-0.71, 3.76)	0.181	2.06 (-0.34, 4.46)	0.093	0.21 (-2.53, 2.95)	0.881	1.08 (-2.34, 4.50)	0.536
**Birth sex * ethnic group * childhood social class**								
Female * Mixed * advantaged	0.67 (-0.61, 1.95)	0.306	0.12 (-1.73, 1.97)	0.901	-0.52 (-2.10, 1.06)	0.518	-1.37 (-3.91, 1.18)	0.293
Female * South Asian * advantaged	-0.19 (-0.91, 0.54)	0.619	-0.03 (-1.13, 1.06)	0.952	0.62 (-0.27, 1.51)	0.173	0.53 (-0.74, 1.79)	0.413
Female * Black * advantaged	-0.15 (-1.60, 1.30)	0.844	-0.84 (-3.23, 1.55)	0.490	0.60 (-1.15, 2.35)	0.502	1.30 (-1.39, 3.98)	0.343
Female * Other * advantaged	0.16 (-1.68, 2.01)	0.862	-0.81 (-3.16, 1.54)	0.499	1.42 (-0.85, 3.68)	0.221	1.29 (-1.38, 3.95)	0.345
**Cohort * birth sex * ethnic group * childhood social class**								
MCS * Female * Mixed * advantaged	0.53 (-1.63, 2.69)	0.632	0.26 (-2.38, 2.91)	0.845	0.62 (-2.05, 3.28)	0.650	3.21 (-0.12, 6.53)	0.058
MCS * Female * South Asian * advantaged	0.36 (-0.84, 1.56)	0.560	-0.17 (-1.95, 1.62)	0.855	-0.91 (-2.37, 0.56)	0.224	-0.01 (-2.42, 2.40)	0.994
MCS * Female * Black * advantaged	1.63 (-0.80, 4.07)	0.188	2.33 (-0.48, 5.14)	0.104	-2.03 (-5.00, 0.95)	0.181	-3.28 (-6.50, -0.06)	0.046
MCS * Female * Other * advantaged	-0.79 (-3.46, 1.88)	0.563	-0.95 (-4.28, 2.38)	0.576	-0.50 (-3.78, 2.77)	0.763	-1.50 (-5.60, 2.61)	0.474
**Sexual orientation * childhood social class**								
Sexual minority * advantaged	-0.12 (-0.92, 0.68)	0.767	-0.34 (-1.88, 1.19)	0.660	0.64 (-0.34, 1.62)	0.202	1.23 (-1.06, 3.52)	0.292
**Cohort * sexual orientation * childhood social class**								
MCS * sexual minority * advantaged	0.34 (-0.89, 1.57)	0.589	1.23 (-0.66, 3.13)	0.201	-0.33 (-1.84, 1.18)	0.669	-2.44 (-5.04, 0.17)	0.066
**Birth sex * sexual orientation * childhood social class**								

Female * sexual minority * advantaged	0.22 (-0.82, 1.27)	0.673	-0.29 (-2.17, 1.59)	0.763	-1.20 (-2.48, 0.09)	0.068	-3.24 (-5.83, -0.66)	0.014
**Cohort * birth sex * sexual orientation * childhood social class**								
MCS * Female * sexual minority * advantaged	-0.43 (-1.94, 1.07)	0.572	0.44 (-2.22, 3.09)	0.747	1.47 (-0.38, 3.33)	0.120	4.92 (1.90, 7.93)	0.001
**Ethnic group * sexual orientation * childhood social class**								
Mixed * sexual minority * advantaged	3.12 (-2.91, 9.15)	0.310	5.85 (1.89, 9.80)	0.004	-7.34 (-14.76, 0.08)	0.053	-3.95 (-8.58, 0.69)	0.095
South Asian * sexual minority * advantaged	-1.86 (-4.29, 0.57)	0.134	-1.40 (-3.65, 0.84)	0.220	2.29 (-0.71, 5.28)	0.134	4.67 (1.84, 7.51)	0.001
Black * sexual minority * advantaged	-1.33 (-5.57, 2.91)	0.538	-0.33 (-2.37, 1.70)	0.749	1.60 (-4.51, 7.71)	0.608	2.62 (-0.67, 5.91)	0.118
Other * sexual minority * advantaged	-4.56 (-7.96, -1.16)	0.009	-4.77 (-7.52, -2.02)	0.001	3.94 (-0.25, 8.13)	0.065	6.67 (3.09, 10.24)	<0.001
**Cohort * ethnic group * sexual orientation * childhood social class**								
MCS * Mixed * sexual minority * advantaged	-2.20 (-6.65, 2.24)	0.331	-6.12 (-9.53, -2.70)	<0.001	4.38 (-1.09, 9.85)	0.117	2.43 (-1.48, 6.34)	0.223
MCS * South Asian * sexual minority * advantaged	0.11 (-3.98, 4.19)	0.958	-0.70 (-3.78, 2.38)	0.655	-0.76 (-5.78, 4.27)	0.768	-1.23 (-5.52, 3.05)	0.573
MCS * Black * sexual minority * advantaged								
MCS * Other * sexual minority * advantaged	3.10 (-2.48, 8.68)	0.276	1.03 (-2.28, 4.34)	0.541	-5.01 (-11.88, 1.85)	0.152	-4.24 (-7.65, -0.84)	0.015
**Birth sex * ethnic group * sexual orientation * childhood social class**								
Female * Mixed * sexual minority * advantaged	-0.98 (-5.72, 3.76)	0.686	-2.19 (-5.65, 1.28)	0.216	3.81 (-2.02, 9.65)	0.200	1.91 (-2.10, 5.91)	0.350
Female * South Asian * sexual minority * advantaged	0.86 (-3.00, 4.73)	0.661	-0.49 (-4.17, 3.18)	0.793	4.96 (-1.69, 11.61)	0.144	4.19 (1.00, 7.38)	0.010
Female * Black * sexual minority * advantaged					-1.96 (-10.00, 6.08)	0.632	-0.74 (-6.82, 5.34)	0.812
Female * Other * sexual minority * advantaged	2.08 (-3.38, 7.55)	0.455	3.53 (0.07, 7.00)	0.046	1.22 (-5.51, 7.95)	0.722	0.04 (-4.02, 4.11)	0.984
**Cohort * birth sex * ethnic group * sexual orientation * childhood social class**								
MCS * Female * South Asian * sexual minority * advantaged					-6.54 (-14.71, 1.64)	0.117	-6.09 (-11.52, -0.67)	0.028
**Intercept**	4.57 (4.38, 4.76)	<0.001	4.70 (4.36, 5.04)	<0.001	6.76 (6.53, 7.00)	<0.001	6.95 (6.60, 7.30)	<0.001

Note. CI: confidence interval; GAD-2: 2-item Generalised Anxiety Disorder questionnaire; MCS: Millennium Cohort Study (born in 2000-2002); NS: Next Steps study (born in 1990); ONS: Office for National Statistics; PHQ-2: 2-item depression Patient Health Questionnaire; UCLA-3: 3-item University of California Los Angeles loneliness scale.
Weighted results are adjusted for survey design characteristics, including survey and non-response weights. White includes all White groups; South Asian includes Bangladeshi, Indian, and Pakistani groups; Black includes Black African, Black Caribbean, and Black British groups; Other includes all other ethnic group not included in the other categories.

Figure S1. Stratum-specific residual values and 95% credible intervals for each outcome using 40 strata. Markov Chain Monte Carlo (MCMC) estimation, unweighted results.

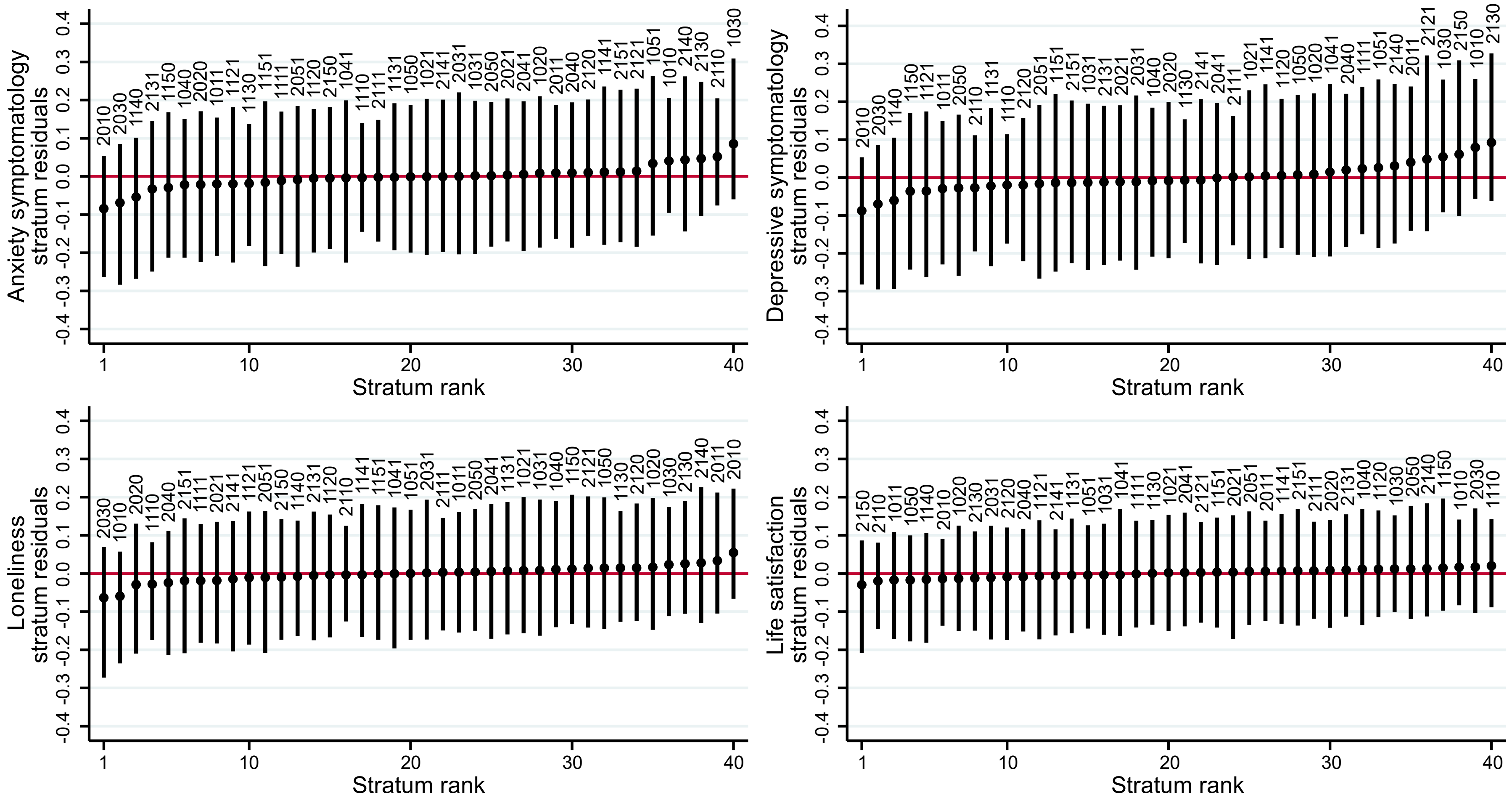

Strata defined by generation/cohort (first digit: 1 Next Steps/1990, 2 Millennium Cohort Study /2000-2002), birth sex (second digit: 0 Male, 1 Female), ethnicity (third digit: 1 White, 2 Mixed, 3 South Asian, 4 Black, 5 Other), sexual orientation (fourth digit: 0 Heterosexual, 1 Sexual minority).

Figure S2. Stratum-specific predicted values and 95% credible intervals for each outcome using 40 strata. Markov Chain Monte Carlo (MCMC) estimation, unweighted results.

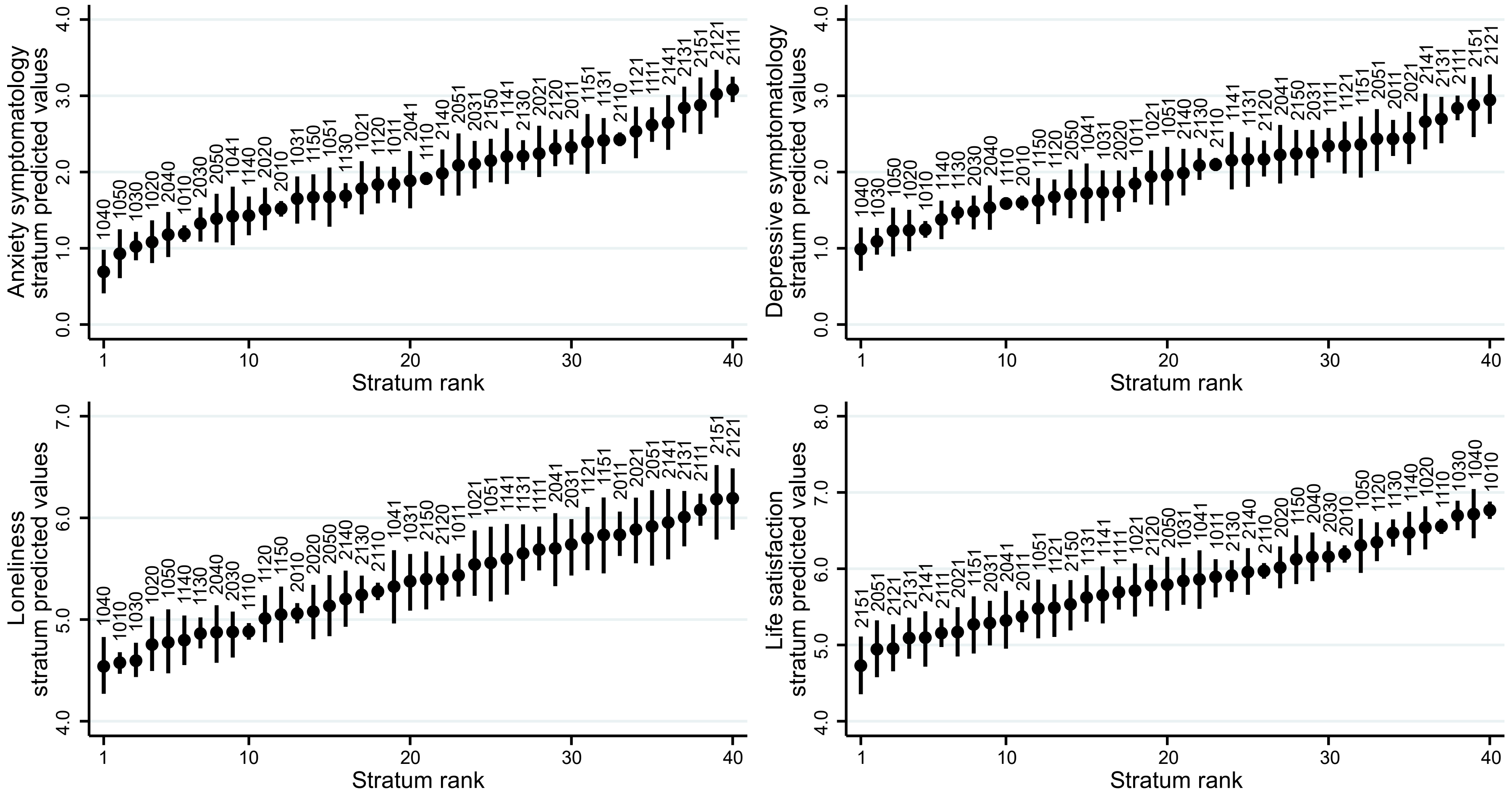

Strata defined by generation/cohort (first digit: 1 Next Steps/1990, 2 Millennium Cohort Study /2000-2002), birth sex (second digit: 0 Male, 1 Female), ethnicity (third digit: 1 White, 2 Mixed, 3 South Asian, 4 Black, 5 Other), sexual orientation (fourth digit: 0 Heterosexual, 1 Sexual minority).

Figure S3. Stratum-specific residual values for each outcome using 40 strata. Maximum likelihood estimation, weighted (survey design and non-response) results.

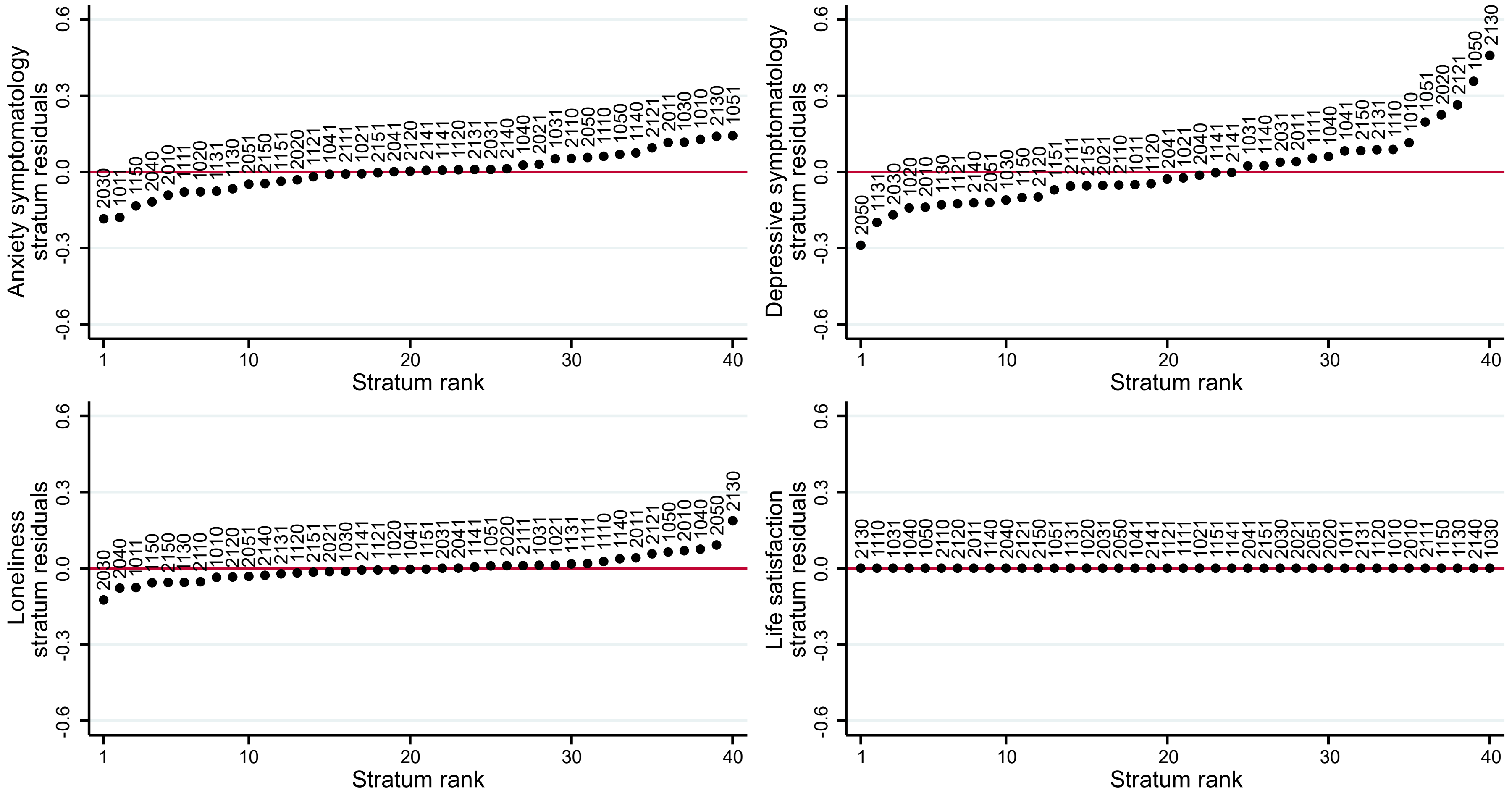

Strata defined by generation/cohort (first digit: 1 Next Steps/1990, 2 Millennium Cohort Study /2000-2002), birth sex (second digit: 0 Male, 1 Female), ethnicity (third digit: 1 White, 2 Mixed, 3 South Asian, 4 Black, 5 Other), sexual orientation (fourth digit: 0 Heterosexual, 1 Sexual minority).

Figure S4. Stratum-specific predicted values for each outcome using 40 strata. Maximum likelihood estimation, weighted (survey design and non-response) results.

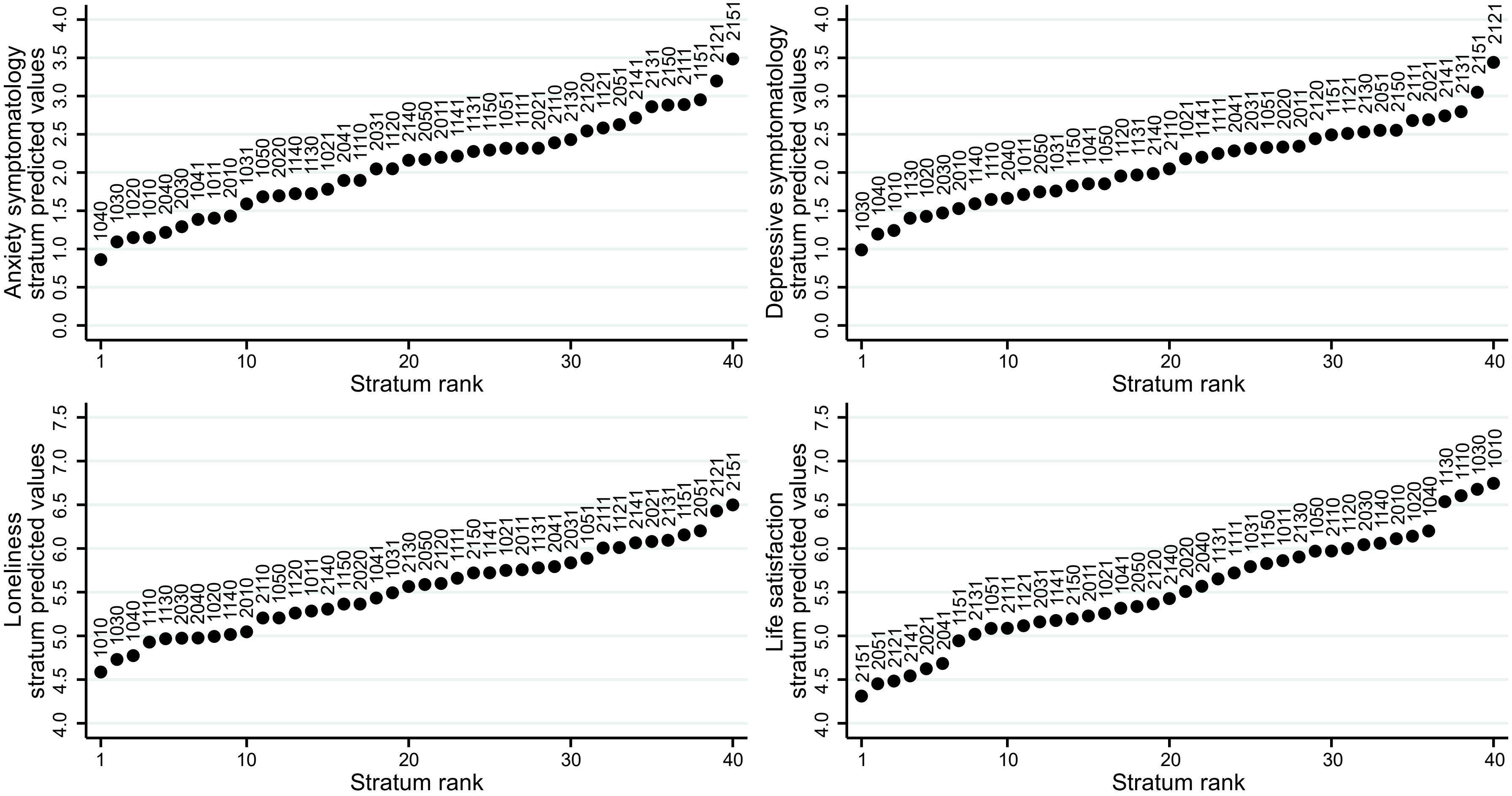

Strata defined by generation/cohort (first digit: 1 Next Steps/1990, 2 Millennium Cohort Study /2000-2002), birth sex (second digit: 0 Male, 1 Female), ethnicity (third digit: 1 White, 2 Mixed, 3 South Asian, 4 Black, 5 Other), sexual orientation (fourth digit: 0 Heterosexual, 1 Sexual minority).

Figure S5. Anxiety and depressive symptomatology predicted values of each intersectional strata. Markov Chain Monte Carlo (MCMC) estimation, unweighted results.

Each of the three parallel grey-shaded lines represents the results using each of the indicators of the socioeconomic position: residential Index of Multiple Deprivation (IMD) rank (leftmost line), housing tenure (centre line), or parental social class during childhood (rightmost line). M: male; F: female. White includes all White groups; South Asian includes Bangladeshi, Indian, and Pakistani groups; Black includes Black African, Black Caribbean, and Black British groups; Other includes all other ethnic group not included in the other categories.

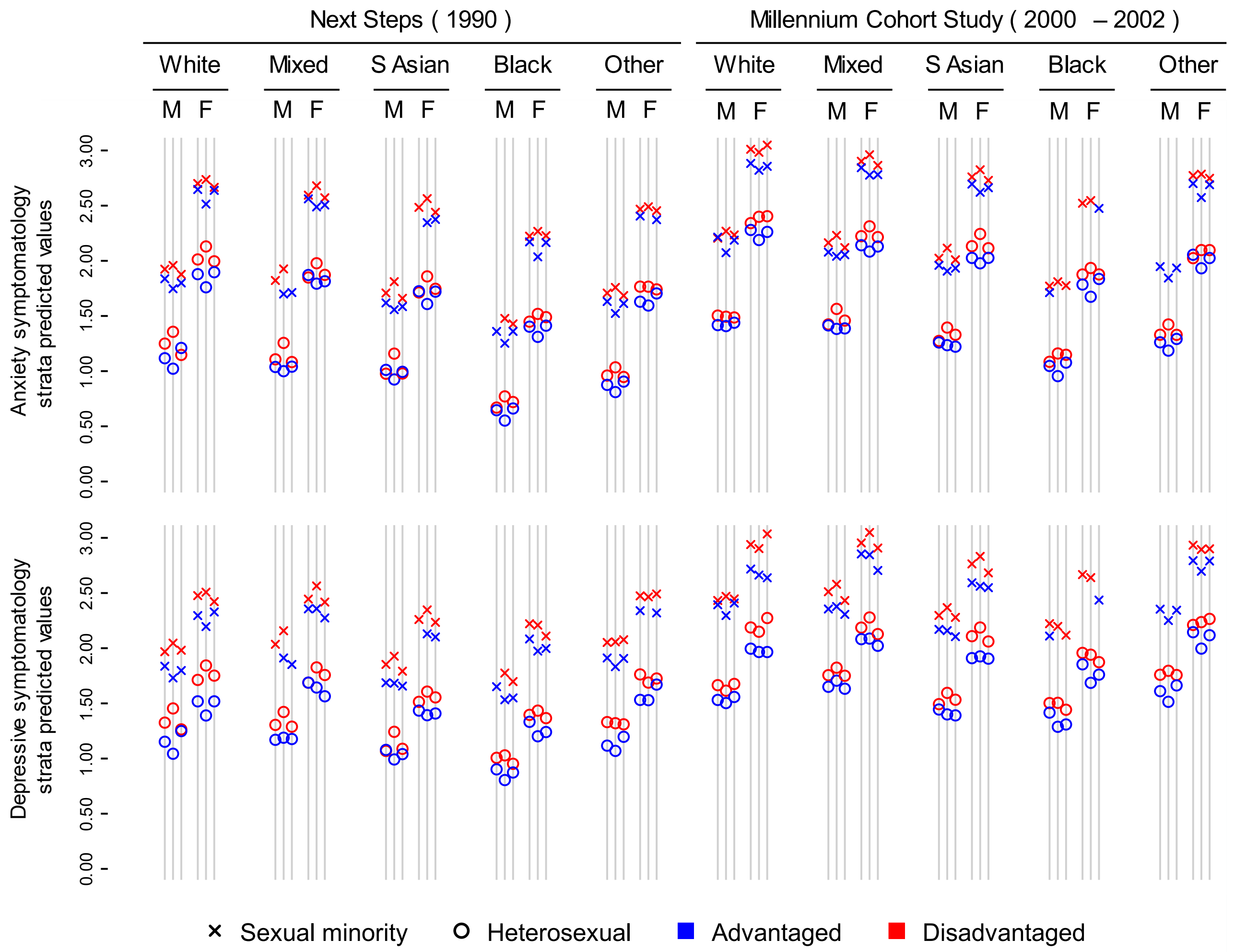

Figure S6. Loneliness and life satisfaction predicted values of each intersectional strata. Markov Chain Monte Carlo (MCMC) estimation, unweighted results.

Each of the three parallel grey-shaded lines represents the results using each of the indicators of the socioeconomic position: residential Index of Multiple Deprivation (IMD) rank (leftmost line), housing tenure (centre line), or parental social class during childhood (rightmost line). M: male; F: female. White includes all White groups; South Asian includes Bangladeshi, Indian, and Pakistani groups; Black includes Black African, Black Caribbean, and Black British groups; Other includes all other ethnic group not included in the other categories.

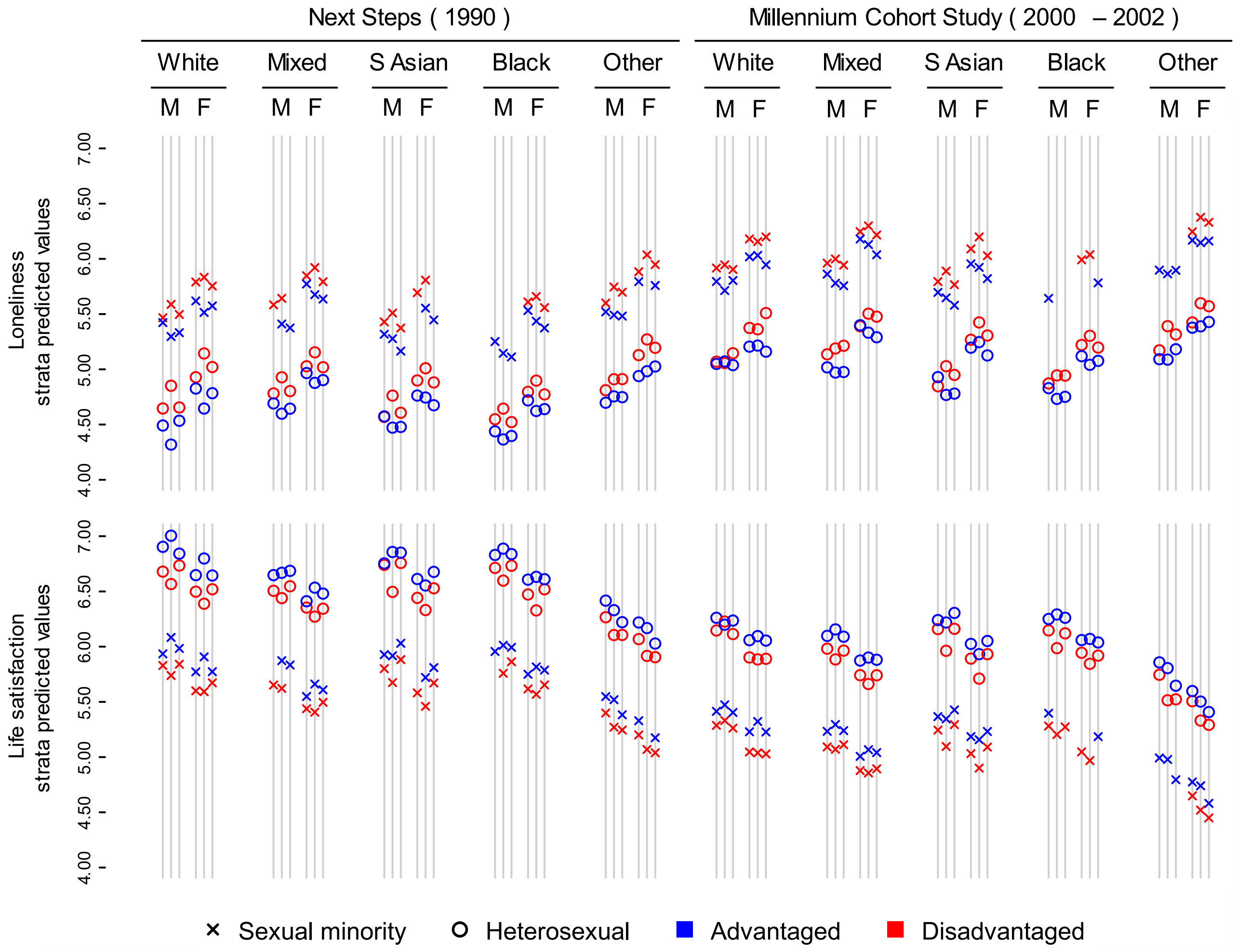

Figure S7. Anxiety and depressive symptomatology predicted values of each intersectional strata. Maximum likelihood estimation, weighted (survey design and non-response) results.

Each of the three parallel grey-shaded lines represents the results using each of the indicators of the socioeconomic position: residential Index of Multiple Deprivation (IMD) rank (leftmost line), housing tenure (centre line), or parental social class during childhood (rightmost line). M: male; F: female. White includes all White groups; South Asian includes Bangladeshi, Indian, and Pakistani groups; Black includes Black African, Black Caribbean, and Black British groups; Other includes all other ethnic group not included in the other categories.

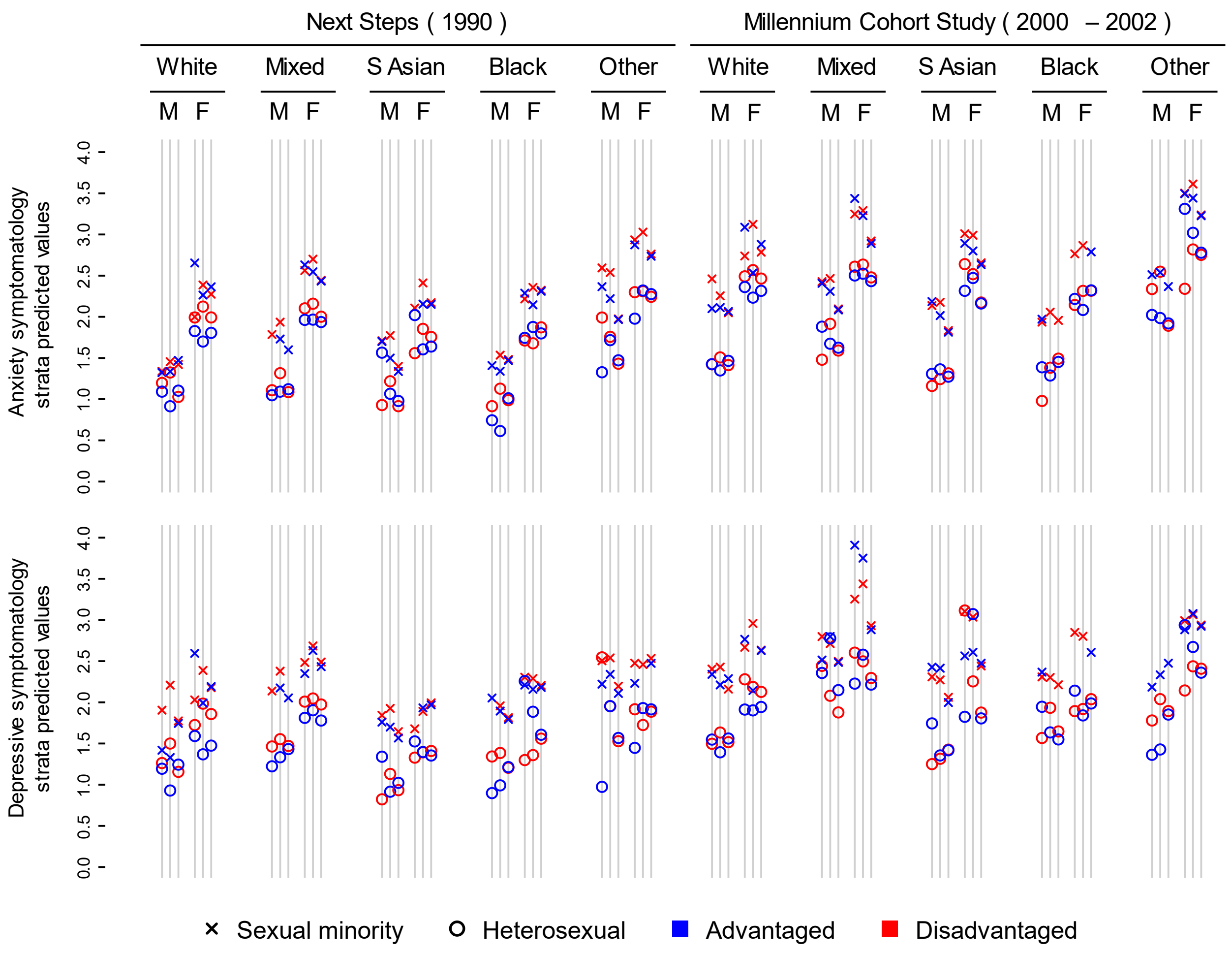

Figure S8. Loneliness and life satisfaction predicted values of each intersectional strata. Maximum likelihood estimation, weighted (survey design and non-response) results.

Each of the three parallel grey-shaded lines represents the results using each of the indicators of the socioeconomic position: residential Index of Multiple Deprivation (IMD) rank (leftmost line), housing tenure (centre line), or parental social class during childhood (rightmost line). M: male; F: female. White includes all White groups; South Asian includes Bangladeshi, Indian, and Pakistani groups; Black includes Black African, Black Caribbean, and Black British groups; Other includes all other ethnic group not included in the other categories.

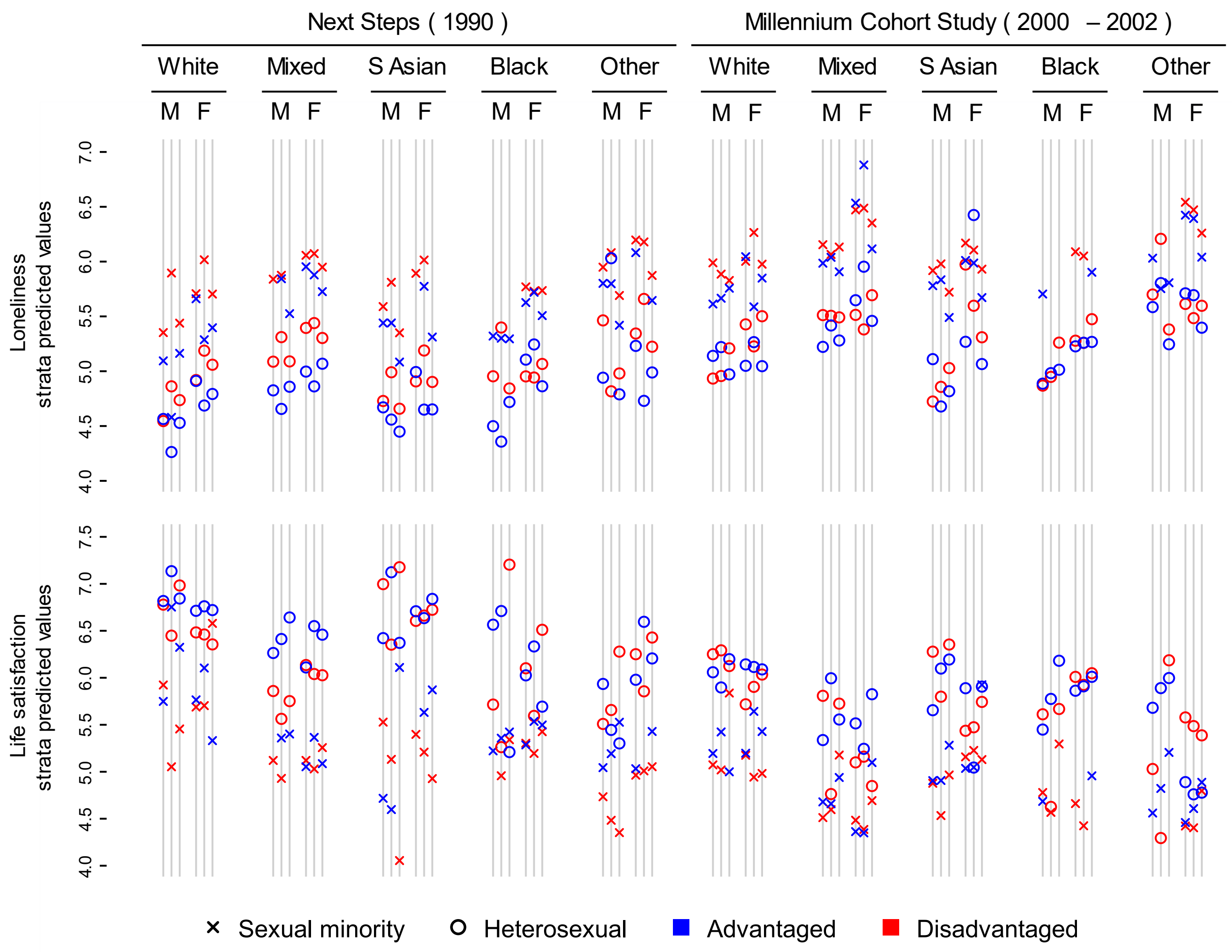

Figure S9. Anxiety and depressive symptomatology residual values (intersectional effects) and 95% credible intervals of each intersectional stratum using housing tenure as the indicator of socioeconomic position. Markov Chain Monte Carlo (MCMC) estimation, unweighted results.

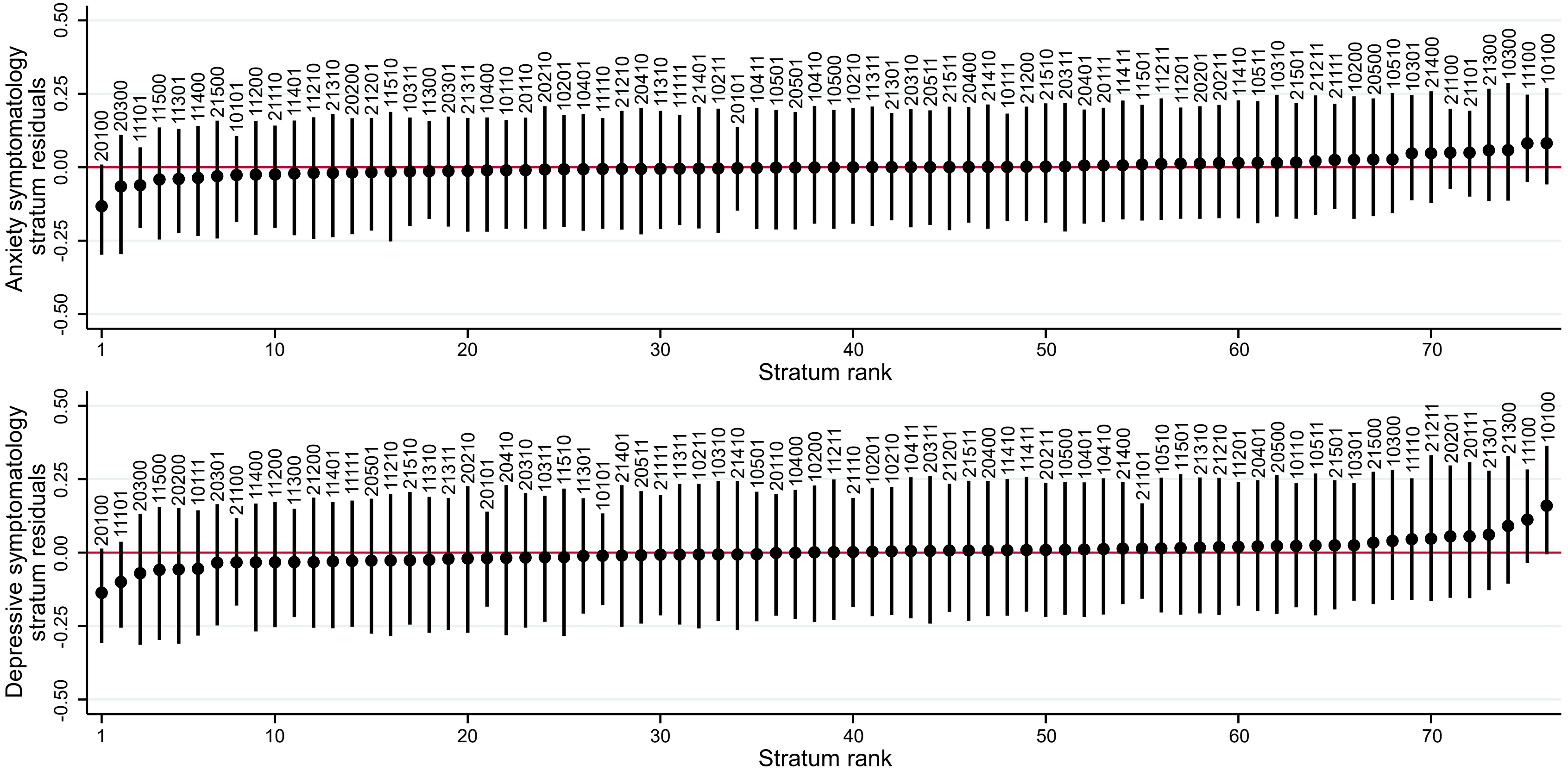

Strata defined by generation/cohort (first digit: 1 Next Steps/1990, 2 Millennium Cohort Study/2000-2002), birth sex (second digit: 0 Male, 1 Female), ethnicity (third digit: 1 White, 2 Mixed, 3 South Asian, 4 Black, 5 Other), sexual orientation (fourth digit: 0 Heterosexual, 1 Sexual minority), housing tenure (fifth digit: 0 Not homeowner, 1 Homeowner/part owner).

Figure S10. Loneliness and life satisfaction residual values (intersectional effects) and 95% credible intervals of each intersectional stratum using housing tenure as the indicator of socioeconomic position. Markov Chain Monte Carlo (MCMC) estimation, unweighted results.

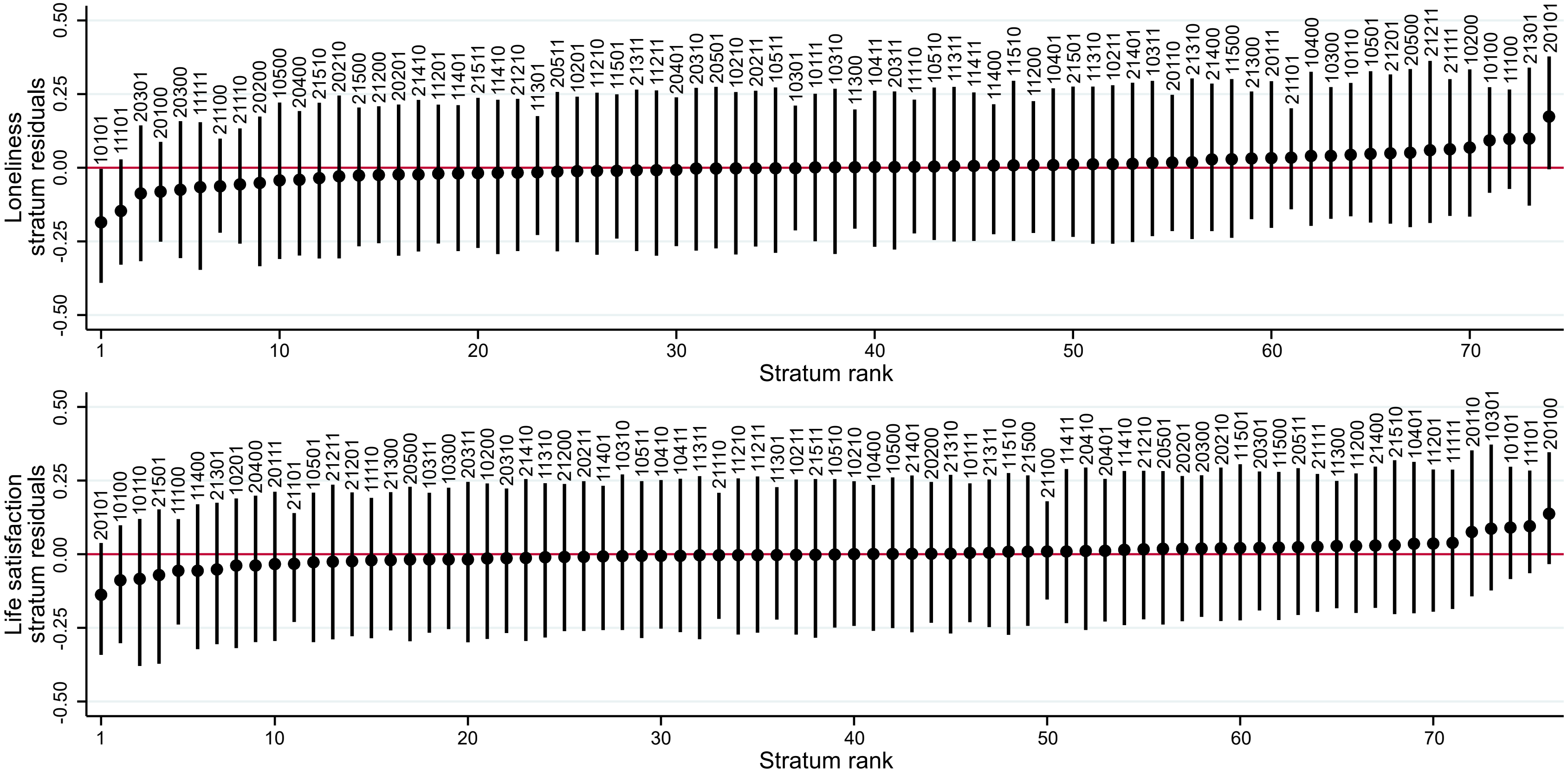

Strata defined by generation/cohort (first digit: 1 Next Steps/1990, 2 Millennium Cohort Study/2000-2002), birth sex (second digit: 0 Male, 1 Female), ethnicity (third digit: 1 White, 2 Mixed, 3 South Asian, 4 Black, 5 Other), sexual orientation (fourth digit: 0 Heterosexual, 1 Sexual minority), housing tenure (fifth digit: 0 Not homeowner, 1 Homeowner/part owner).

Figure S11. Anxiety and depressive symptomatology residual values (intersectional effects) and 95% credible intervals of each intersectional stratum using parental social class during childhood as the indicator of socioeconomic position. Markov Chain Monte Carlo (MCMC) estimation, unweighted results.

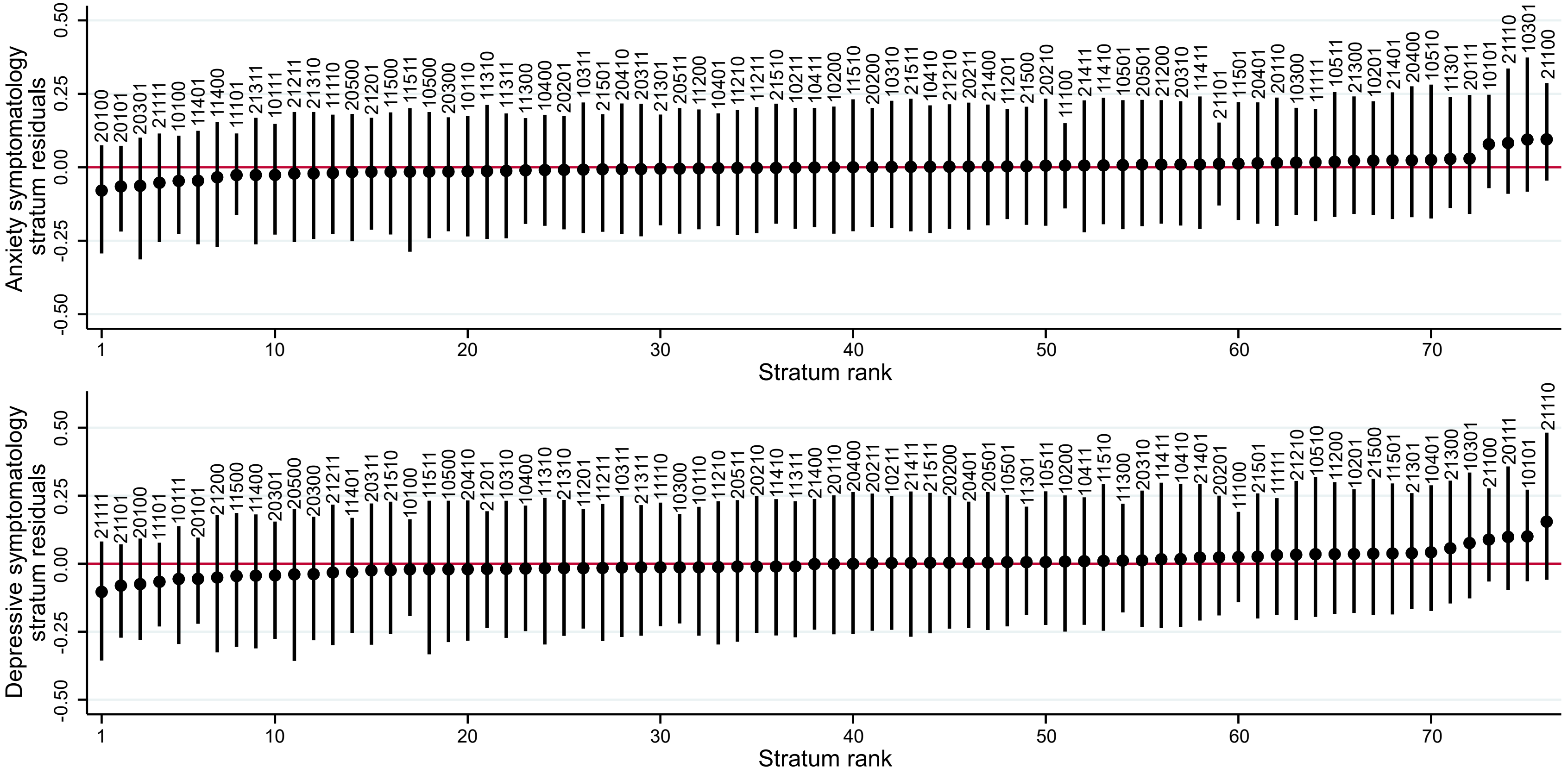

Strata defined by generation/cohort (first digit: 1 Next Steps/1990, 2 Millennium Cohort Study/2000-2002), birth sex (second digit: 0 Male, 1 Female), ethnicity (third digit: 1 White, 2 Mixed, 3 South Asian, 4 Black, 5 Other), sexual orientation (fourth digit: 0 Heterosexual, 1 Sexual minority), parental social class during childhood (fifth digit: 0 Disadvantaged, 1 Advantaged).

Figure S12. Loneliness and life satisfaction residual values (intersectional effects) and 95% credible intervals of each intersectional stratum using parental social class during childhood as the indicator of socioeconomic position. Markov Chain Monte Carlo (MCMC) estimation, unweighted results.

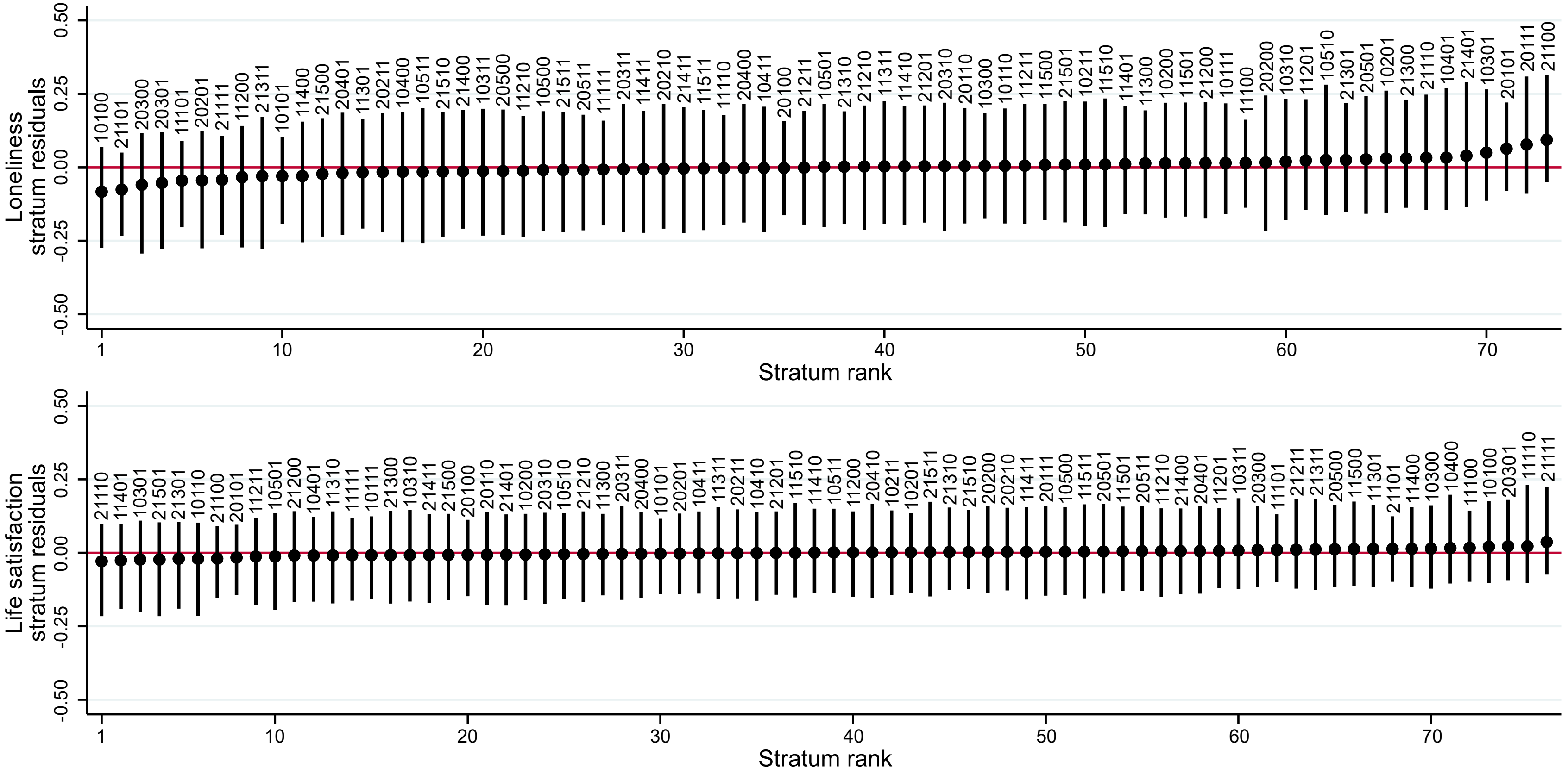

Strata defined by generation/cohort (first digit: 1 Next Steps/1990, 2 Millennium Cohort Study/2000-2002), birth sex (second digit: 0 Male, 1 Female), ethnicity (third digit: 1 White, 2 Mixed, 3 South Asian, 4 Black, 5 Other), sexual orientation (fourth digit: 0 Heterosexual, 1 Sexual minority), parental social class during childhood (fifth digit: 0 Disadvantaged, 1 Advantaged).

Figure S13. Anxiety and depressive symptomatology residual values (intersectional effects) using residential Index of Multiple Deprivation (IMD) rank as the indicator of socioeconomic position. Maximum likelihood estimation, weighted (survey design and non-response) results.

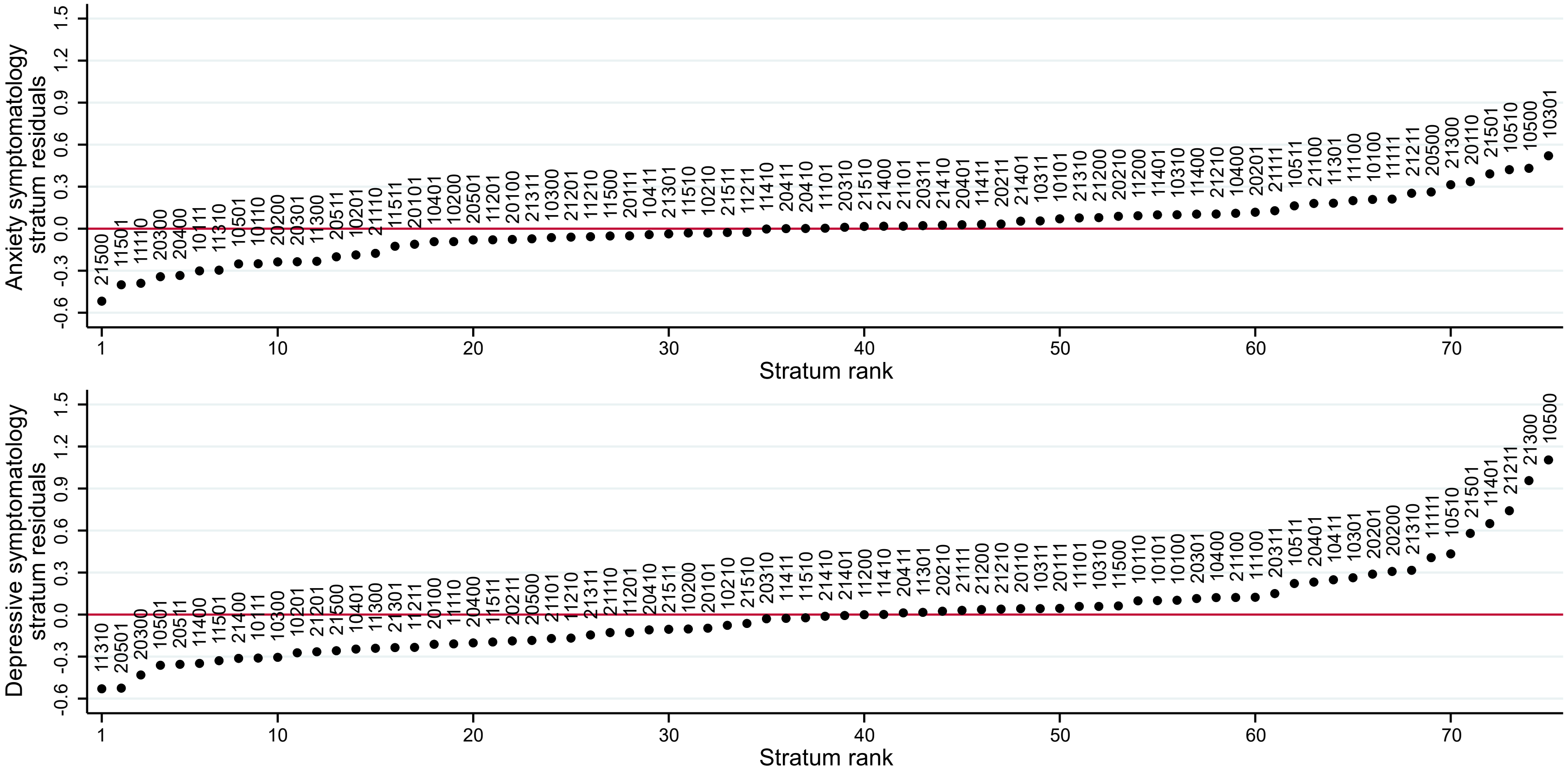

Strata defined by generation/cohort (first digit: 1 Next Steps/1990, 2 Millennium Cohort Study/2000-2002), birth sex (second digit: 0 Male, 1 Female), ethnicity (third digit: 1 White, 2 Mixed, 3 South Asian, 4 Black, 5 Other), sexual orientation (fourth digit: 0 Heterosexual, 1 Sexual minority), residential IMD rank (fifth digit: 0 More deprived, 1 Less deprived).

Figure S14. Loneliness and life satisfaction residual values (intersectional effects) using residential Index of Multiple Deprivation (IMD) rank as the indicator of socioeconomic position. Maximum likelihood estimation, weighted (survey design and non-response) results.

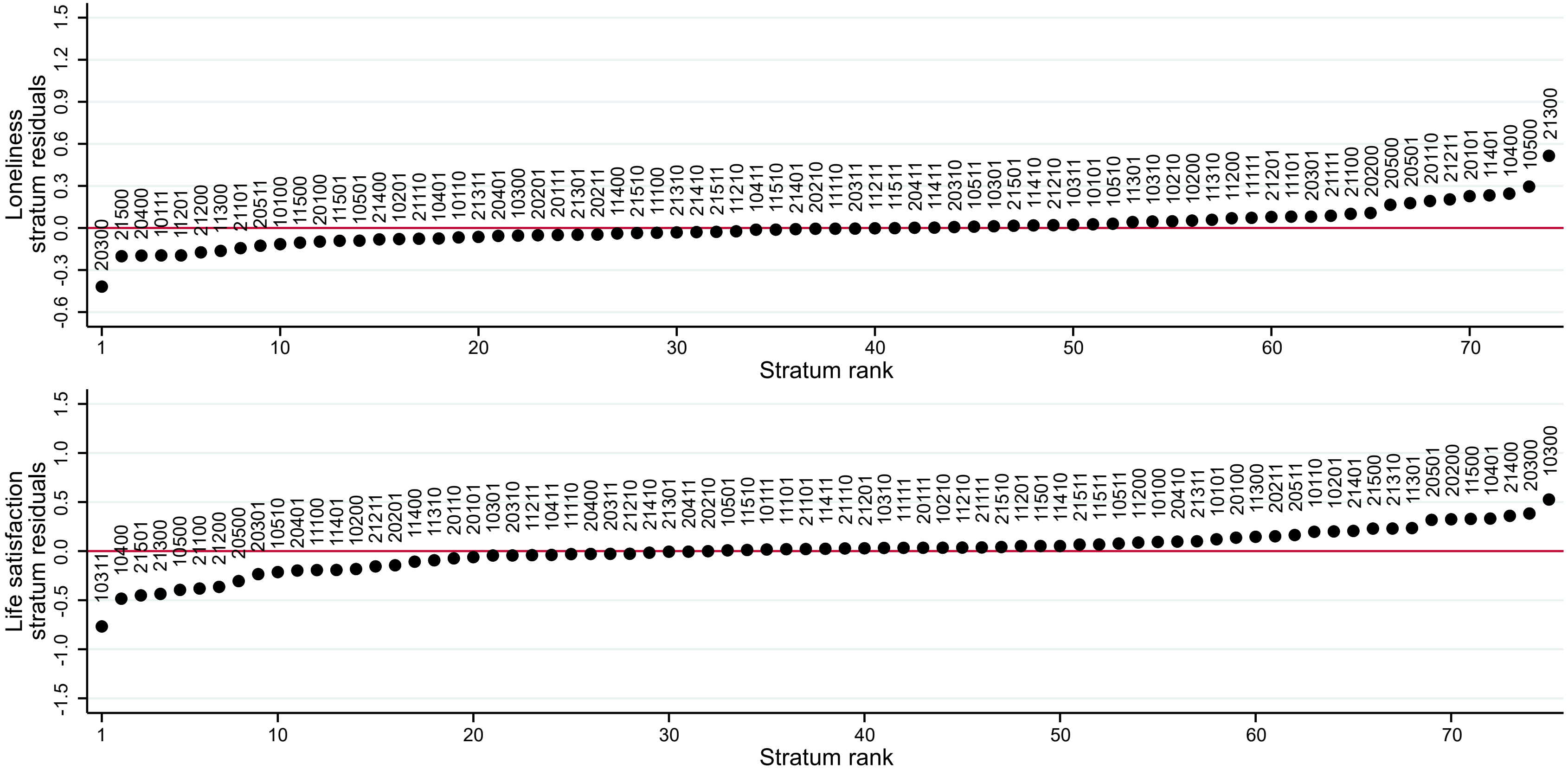

Strata defined by generation/cohort (first digit: 1 Next Steps/1990, 2 Millennium Cohort Study/2000-2002), birth sex (second digit: 0 Male, 1 Female), ethnicity (third digit: 1 White, 2 Mixed, 3 South Asian, 4 Black, 5 Other), sexual orientation (fourth digit: 0 Heterosexual, 1 Sexual minority), residential IMD rank (fifth digit: 0 More deprived, 1 Less deprived).

Figure S15. Anxiety and depressive symptomatology residual values (intersectional effects) using housing tenure as the indicator of socioeconomic position. Maximum likelihood estimation, weighted (survey design and non-response) results.

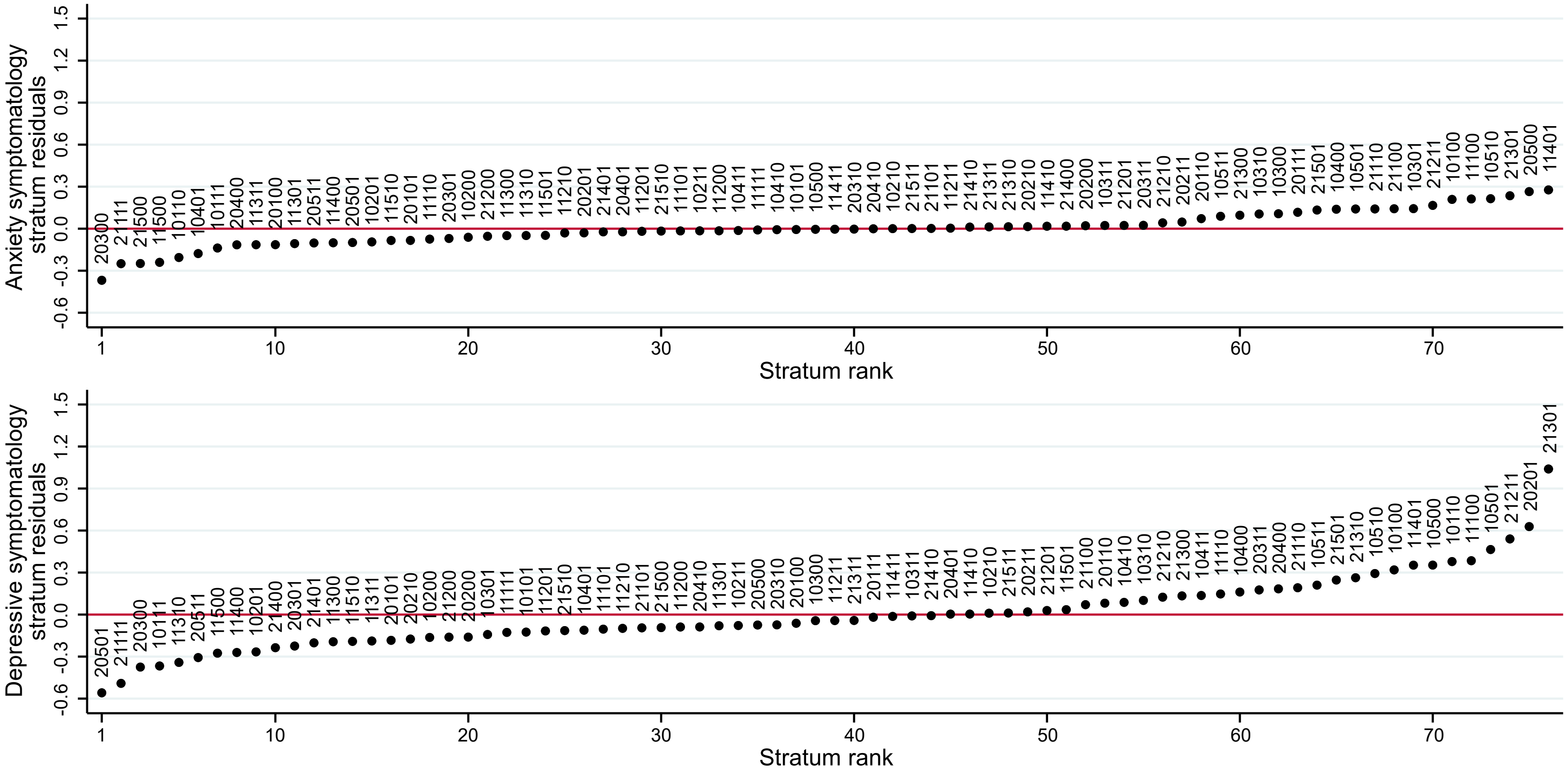

Strata defined by generation/cohort (first digit: 1 Next Steps/1990, 2 Millennium Cohort Study/2000-2002), birth sex (second digit: 0 Male, 1 Female), ethnicity (third digit: 1 White, 2 Mixed, 3 South Asian, 4 Black, 5 Other), sexual orientation (fourth digit: 0 Heterosexual, 1 Sexual minority), housing tenure (fifth digit: 0 Not homeowner, 1 Homeowner/part owner).

Figure S16. Loneliness and life satisfaction residual values (intersectional effects) using housing tenure as the indicator of socioeconomic position. Maximum likelihood estimation, weighted (survey design and non-response) results.

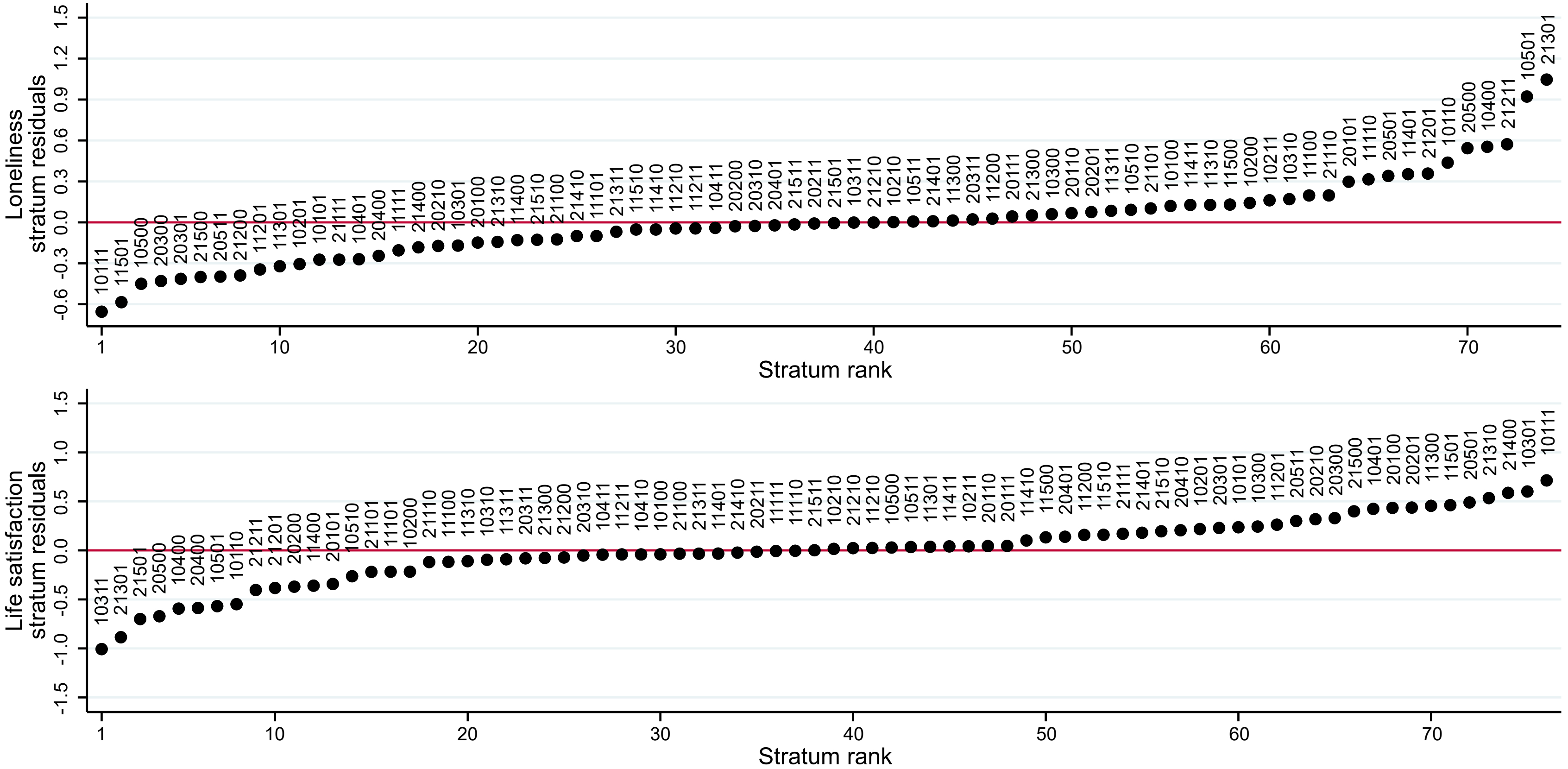

Strata defined by generation/cohort (first digit: 1 Next Steps/1990, 2 Millennium Cohort Study/2000-2002), birth sex (second digit: 0 Male, 1 Female), ethnicity (third digit: 1 White, 2 Mixed, 3 South Asian, 4 Black, 5 Other), sexual orientation (fourth digit: 0 Heterosexual, 1 Sexual minority), housing tenure (fifth digit: 0 Not homeowner, 1 Homeowner/part owner).

Figure S17. Anxiety and depressive symptomatology residual values (intersectional effects) using parental social class during childhood as the indicator of socioeconomic position. Maximum likelihood estimation, weighted (survey design and non-response) results.

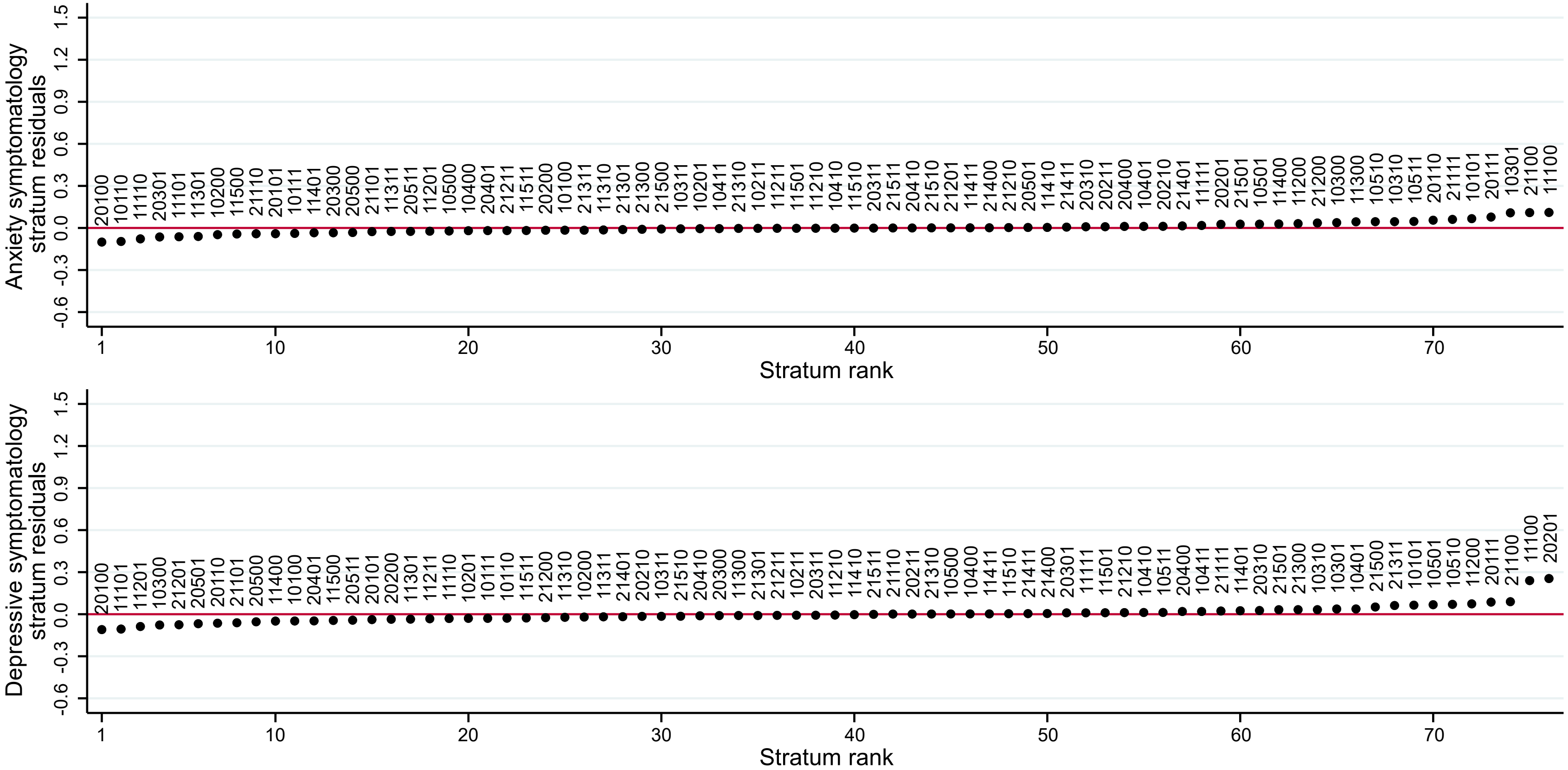

Strata defined by generation/cohort (first digit: 1 Next Steps/1990, 2 Millennium Cohort Study/2000-2002), birth sex (second digit: 0 Male, 1 Female), ethnicity (third digit: 1 White, 2 Mixed, 3 South Asian, 4 Black, 5 Other), sexual orientation (fourth digit: 0 Heterosexual, 1 Sexual minority), parental social class during childhood (fifth digit: 0 Disadvantaged, 1 Advantaged).

Figure S18. Loneliness and life satisfaction residual values (intersectional effects) using parental social class during childhood as the indicator of socioeconomic position. Maximum likelihood estimation, weighted (survey design and non-response) results.

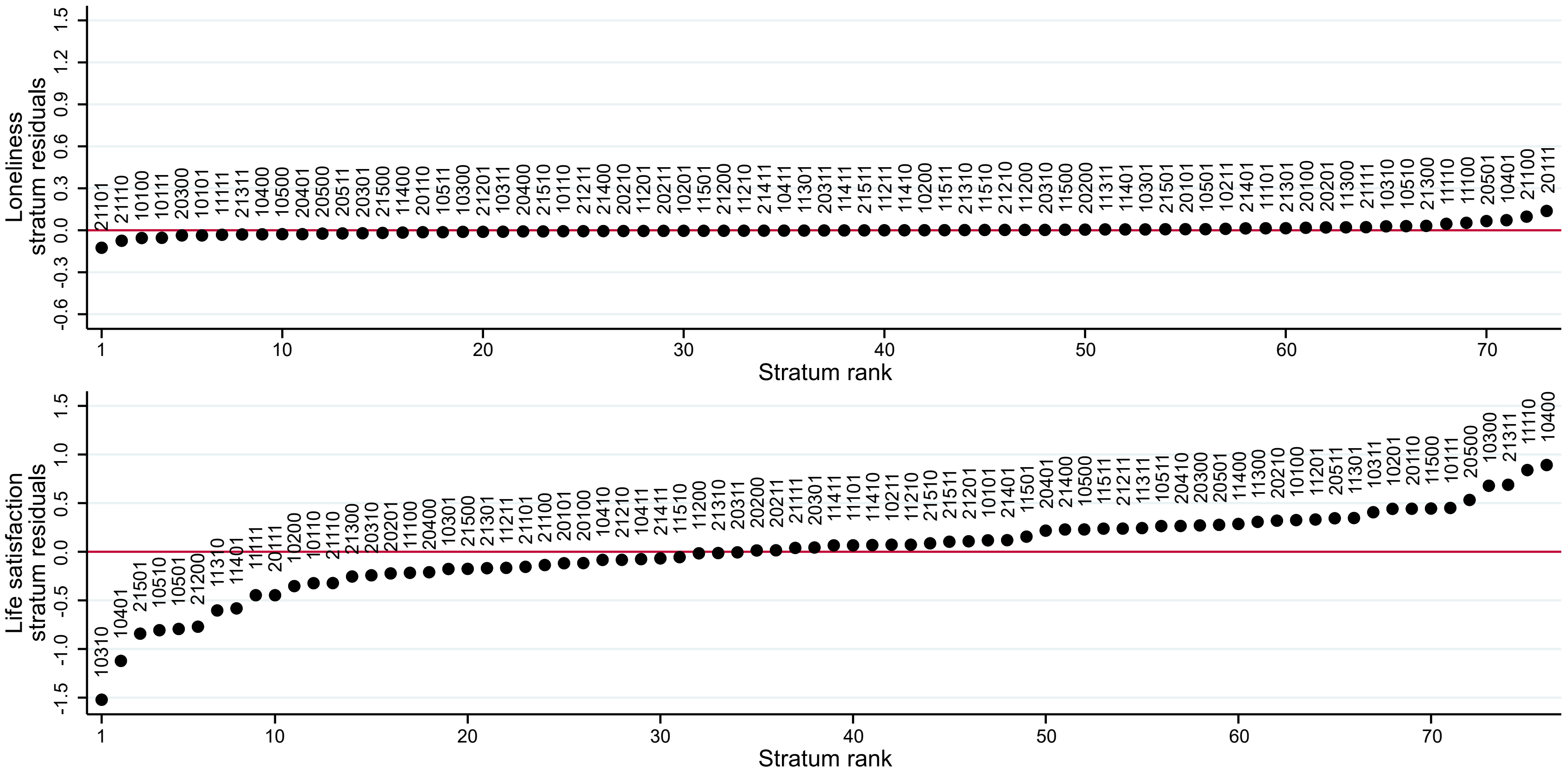

Strata defined by generation/cohort (first digit: 1 Next Steps/1990, 2 Millennium Cohort Study/2000-2002), birth sex (second digit: 0 Male, 1 Female), ethnicity (third digit: 1 White, 2 Mixed, 3 South Asian, 4 Black, 5 Other), sexual orientation (fourth digit: 0 Heterosexual, 1 Sexual minority), parental social class during childhood (fifth digit: 0 Disadvantaged, 1 Advantaged).

Supplementary references

1. Brown M, Goodman A, Peters A, Ploubidis GB, Sanchez A, Silverwood R, Smith K (2021) COVID-19 Survey in Five National Longitudinal Studies: Waves 1, 2 and 3 User Guide (Version 3). UCL Centre for Longitudinal Studies and MRC Unit for Lifelong Health and Ageing, London

2. Mostafa T, Narayanan M, Pongiglione B, Dodgeon B, Goodman A, Silverwood RJ, Ploubidis GB (2021) Missing at random assumption made more plausible: evidence from the 1958 British birth cohort. J Clin Epidemiol 136:44-54. doi:10.1016/j.jclinepi.2021.02.019

3. Silverwood R, Calderwood L, Sakshaug JW, Ploubidis GB (2020) A data driven approach to understanding and handling non-response in the Next Steps cohort. CLS Working Paper 2020/5

4. Evans CR (2019) Adding interactions to models of intersectional health inequalities: Comparing multilevel and conventional methods. Soc Sci Med 221:95-105. doi:10.1016/j.socscimed.2018.11.036

5. Evans CR, Erickson N (2019) Intersectionality and depression in adolescence and early adulthood: A MAIHDA analysis of the national longitudinal study of adolescent to adult health, 1995-2008. Soc Sci Med 220:1-11. doi:10.1016/j.socscimed.2018.10.019

6. Axelsson Fisk S, Mulinari S, Wemrell M, Leckie G, Perez Vicente R, Merlo J (2018) Chronic Obstructive Pulmonary Disease in Sweden: An intersectional multilevel analysis of individual heterogeneity and discriminatory accuracy. SSM Popul Health 4:334-346. doi:10.1016/j.ssmph.2018.03.005

7. Persmark A, Wemrell M, Zettermark S, Leckie G, Subramanian SV, Merlo J (2019) Precision public health: Mapping socioeconomic disparities in opioid dispensations at Swedish pharmacies by Multilevel Analysis of Individual Heterogeneity and Discriminatory Accuracy (MAIHDA). PLoS One 14 (8):e0220322. doi:10.1371/journal.pone.0220322

8. Evans CR, Williams DR, Onnela JP, Subramanian SV (2018) A multilevel approach to modeling health inequalities at the intersection of multiple social identities. Soc Sci Med 203:64-73. doi:10.1016/j.socscimed.2017.11.011

9. Bauer GR, Churchill SM, Mahendran M, Walwyn C, Lizotte D, Villa-Rueda AA (2021) Intersectionality in quantitative research: A systematic review of its emergence and applications of theory and methods. SSM - Population Health 14:100798. doi:10.1016/j.ssmph.2021.100798
